# Theoretical study on preference of open polymer vs. cyclic products in CO_2_/epoxide copolymerization with cobalt(III)-salen bifunctional catalysts

**DOI:** 10.1007/s00894-020-04364-x

**Published:** 2020-05-06

**Authors:** Aleksandra Roznowska, Karol Dyduch, Bun Yeoul Lee, Artur Michalak

**Affiliations:** 1 Department of Theoretical Chemistry, Faculty of Chemistry, Jagiellonian University, Gronostajowa 2, 30-387 Krakow, Poland; 2 Department of Molecular Science and Technology, Ajou University, Suwon, South Korea

**Keywords:** CO2 / epoxide copolymerization, bifunctional Co(III) salen catalysts, copolymerization vs. cyclization, conformational space

## Abstract

The preference of open chain of growing macromolecule vs. possible cyclic form was examined for the bifunctional cobalt(III)-salen catalyst for the copolymerization of CO_2_ with epoxides. A variety of possible isomers was considered (resulting from t*rans*/*cis-β* salen arrangement, different mutual orientation of quaternary ammonium-chains, and possible binding modes). To explore the conformational space, a combined approach was applied, utilizing semiempirical (PM7) MD and the DFT calculations. The preference of the open and cyclic macromolecules attached to the metal center was compared with the corresponding results for isolated model macromolecules, and the systems built of the macromolecule interacting with the tetra-butyl ammonium cation. Result shows that the cyclic structures are strongly preferred for isolated ions, with relatively low cyclization barriers. In the field of positive point charge, the open structures are strongly preferred. For the ions interacting with tetrabutyl ammonium cation, the cyclic structures are preferred, due to delocalization of the positive charge in the cation. For the complexes involving model and “real” Co(III)-salen catalysts, the open structures are strongly preferred. The possible cyclization by dissociation of alkoxide and its transfer to the neighborhood of quaternary ammonium cation is characterized by high activation barriers. Further, the transfer of alkoxide from the metal center to the cation is less likely than the transfer of carbonate, since the metal-alkoxide bond-energy energy is much stronger than energy of metal-carbonate bonding, as shown by ETS-NOCV results. The conclusions are in qualitative agreement with experimental data showing high selectivity towards copolymer formation in the copolymerization processes catalyzed by bifunctional Co(III) salen-complexes.

**Electronic supplementary material:**

The online version of this article (10.1007/s00894-020-04364-x) contains supplementary material, which is available to authorized users.

**Figure:**
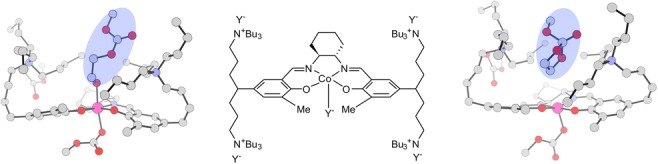
Graphical abstract

## Introduction

**Fig. 1:**
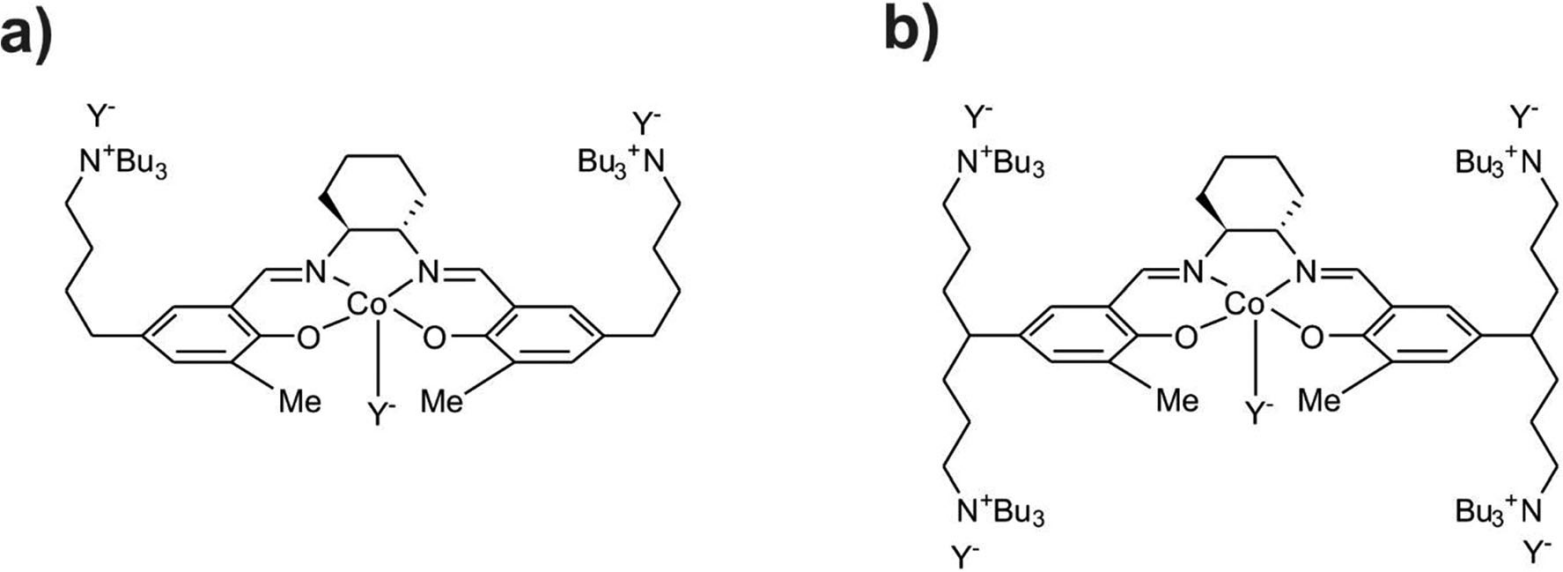
Examples of highly active, bifunctional catalysts for copolymerization of CO_2_ with epoxides. Y^−^ in the initial catalysts complex can be phenolate, acetate, nitrate, etc., and during the catalytic cycle—growing macromolecule (carbonate- or alkoxide-ended)

Copolymerization of CO_2_ with epoxides [1–18] has recently drawn much attention as an example of a possible route to useful products utilizing CO_2_ [19–21], giving rise to biodegradable, environment-friendly polycarbonate materials. Since the initial work by Inoue et al. in 1969 on the zinc-based heterogeneous catalyst [6] and Al-porphyrin single-site systems [7], a significant amount of research has been directed toward a design of other catalysts [8–16]. The family of Co(III) complexes with salen-based ligands is of particular importance. In the binary catalysts, the metal-salen complex is accompanied by the co-catalyst, e.g., onium salt. Remarkable increase in the catalyst activity has been accomplished by designing the bifunctional catalysts in which the salen core is tethering the co-catalyst salts (N^+^-chains) [15–18]; this allows to keep the growing macromolecules (anionic) in the vicinity of the metal center of the complex. Examples of highly-active cobalt(III)-salen bifunctional catalysts are shown in Fig. 1.

**Fig. 2:**
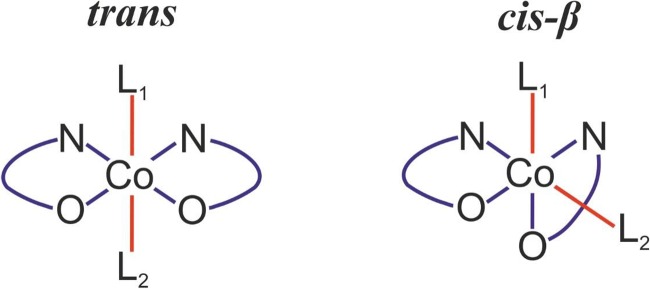
*Trans* and *cis-β* isomers, resulting from different arrangements of salen-type ligand in Co(III) complexes

Among the factors of possible importance for high catalytic activity of the bifunctional Co-salen catalysts, a capability to adopt alternative configurations at the metal center, *trans*, *cis-β* (Fig. 2) has been considered [18]. These two arrangements of the salen ligand in various complexes were experimentally reported [22–24].

In our recent article, the effect of the quaternary ammonium salts on the stability of these alternative isomers of bifunctional catalysts was studied based on the complex computational protocol, involving molecular dynamics on the semi-empirical (PM7) level and the static DFT calculations [25]. The results indicate that a preference of the *trans* or *cis-β* isomers is strongly influenced by the presence and the composition of quaternary ammonium salt attached to the salen-core; also, the *trans/cis-β* isomerization can proceed with relatively low barriers [25]. Thus, in the studies on various mechanistic aspects of the copolymerization process, the possible presence of both, *trans*, and *cis-β* isomers should be considered.

**Fig. 3:**
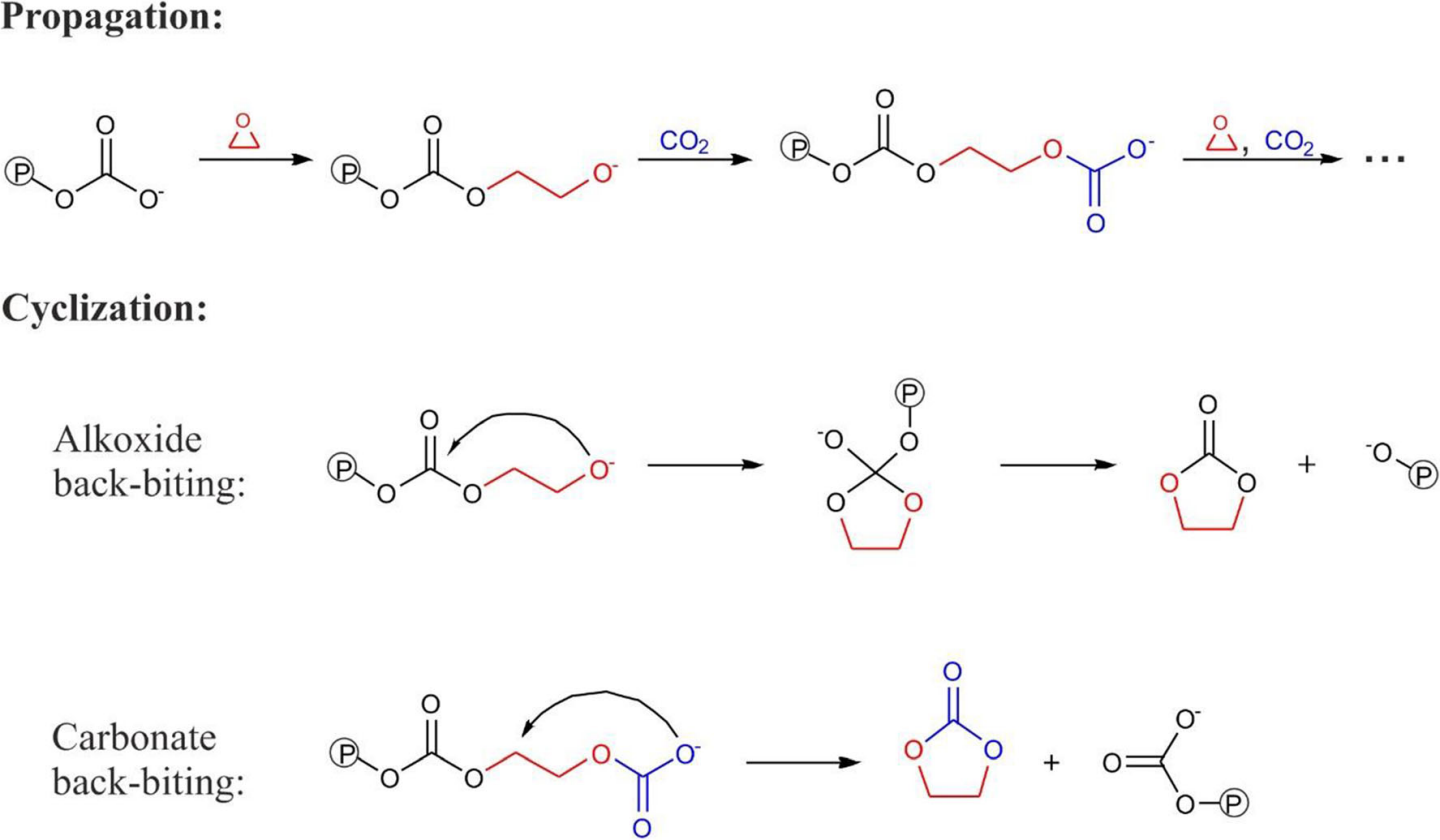
Two main propagation steps in the copolymerization of CO_2_ with epoxides (epoxide ring-opening and CO_2_ insertion), and two possible cyclization mechanisms (alkoxide- and carbonate back-biting) for the process involving ethylene oxide, as an example. The symbol Ⓟ stands for growing-macromolecule chain. The elementary reactions occur at the catalyst, omitted in figure

A general mechanism of CO_2_/epoxide copolymerization [1] is shown in Fig. 3. It includes two main propagation steps: (i) epoxide opening by nucleophilic attack of the carbonate-headed growing macromolecule attached to the metal center, to give alkoxide-ended system; (ii) CO_2_ attachment leading to carbonate species, extended by CH_2_-CHR-O-COO^−^ (or CHR-CH_2_-O-COO^−^) group compared to initial carbonate system. A process of polymer-growth can be terminated by a cyclization reaction. In principle, formation of the cyclic product can occur via carbonate back-biting or alkoxide back-biting mechanism, also depicted in Fig. 3. It was shown [26–28] that the former mechanism has usually much higher activation barriers than the latter.

The main goal of the present account is to compare the energetic preference of open -chain of growing macromolecule vs. possible cyclic form, resulting from the alkoxide back-biting in the process of CO_2_ copolymerization with oxiranes (ethylene oxide and propylene oxide) catalyzed by the bifunctional, cobalt(III) complexes with salen-type ligands tethered by two quaternary ammonium salts (Fig. 1a). We will consider here both, *trans*, and *cis-β* isomers of the salen-core. Further, the preference of the open and cyclic macromolecules attached to the metal center will be compared with the corresponding results for isolated model macromolecules, and the systems built of the macromolecule interacting with the tetra-butyl ammonium cation. It should be emphasized here that preference of open vs. cyclic species in the metal-free process, as well as for other Co(III), and Cr(III)-salen catalyst were studied in details for various epoxides by Darensbourg and Yeung [26–28]. We have performed here similar calculations for isolated open, and cyclic ions, to enable comparison of the results for models, with and without the catalyst, obtained with the same computational methodology.

## Models

**Fig. 4:**
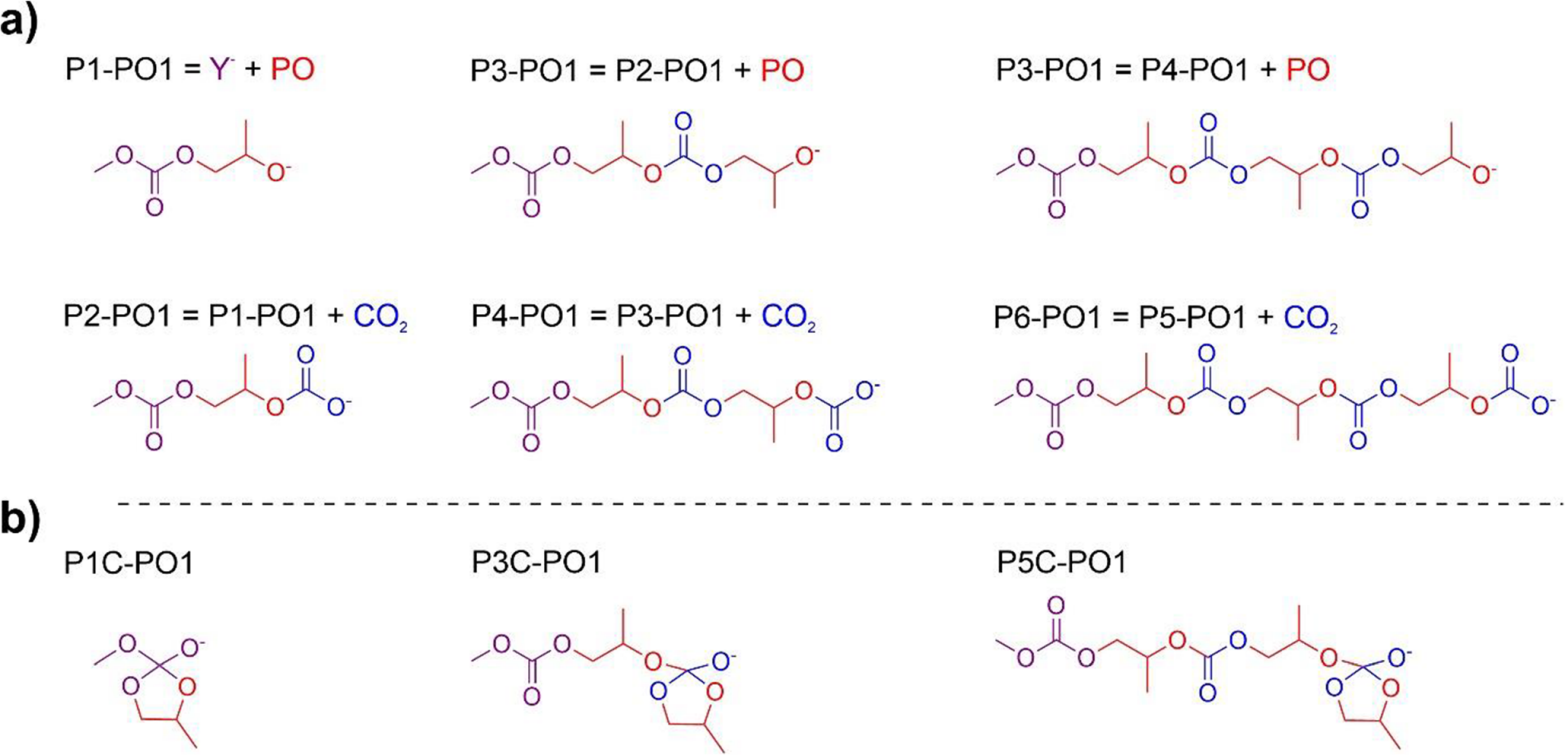
The open-chain structures (**P1-PO1**, …, **P6-PO1**) resulting from a sequence of the co-monomer insertion reactions in CO_2_/propylene oxide copolymerization considered in the present work (part **a**), and the corresponding cyclic structures (**P1C-PO1**, **P3C-PO1**, **P5C-PO1**) (**b**) resulting from the alkoxide back-biting (see Fig. 2)

**Fig. 5:**
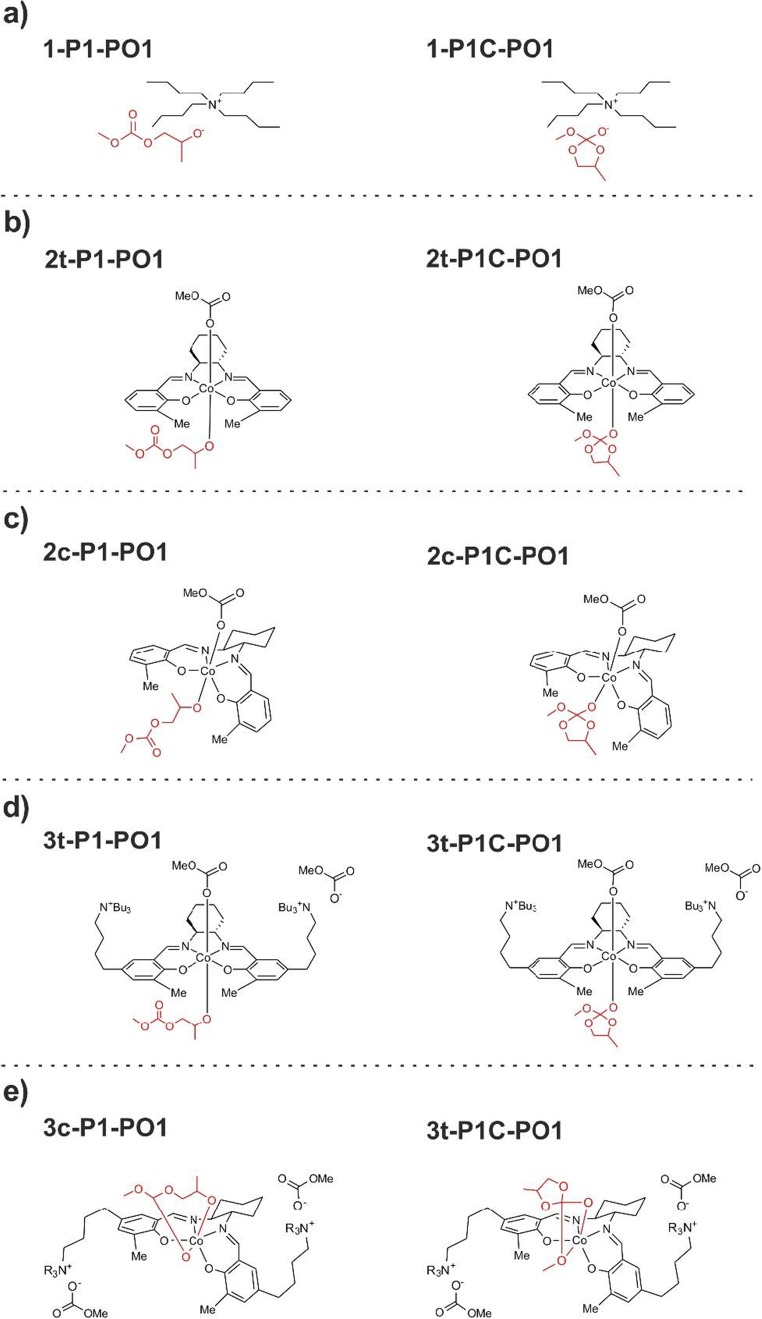
The model systems considered in the present work, involving open and cyclic alkoxides, and the quaternary ammonium cation (part **a**), the *trans* and *cis-β* Co(III)-salen complexes (parts **b** and **c**, respectively), the *trans* and *cis-β* isomers of the “real” bifunctional. Co(III)-salen catalyst tethering two N^+^-chains (parts **d**, and **e**, respectively). As an example, the smallest ‘macromolecule’ (P1) is used, resulting from the methylene-ring opening of propylene oxide (see text)

The models considered in the present work are shown in Figs. 4 and 5. The sequence of the co-monomer insertion reactions in CO_2_/propylene oxide copolymerization, leading to the intermediates **Px-PO1**, *x* **= 1..6**, is presented in Fig. 4a; it should be pointed out that all these systems are anionic. The corresponding cyclic intermediates resulting from alkoxide back-biting (**P1C-PO1**, **P3C-PO1**, **P5C-PO1**) are shown in Fig. 4b. However, in the case of propylene oxide opening, the regioselectivity must be also considered, i.e., two pathways corresponding to the attack of carbonate on the methylene carbon (-CH_2_-), or on the methine carbon atom (methyl-substituted, -CH(CH_3_)-). In the examples in Fig. 4a, b the methylene-ring-opening is shown (-**PO1** suffix used in the labels**)**. For the species resulting from the alternative methine-ring-opening, the suffix –**PO2** will be used (systems **Px-PO2** vs. **PxC-PO2** for *x* = 1,3,5**)**. The corresponding systems for copolymerization of CO_2_ with ethylene oxide will be labeled using **–EO** suffix, i.e., as **Px-EO** vs. **PxC-EO**, *x* = 1,3,5.

The models of open and cyclic macromolecule interacting with tetra-butyl ammonium cation are shown in Fig. 5a using **1-** prefix (**1-Px-PO1** vs. **1-PxC-PO1** for *x* **=** 1,3**)** for the example of methylene-ring-opening of propylene oxide (**–PO1**). For the corresponding systems resulting from the methine-ring-opening of propylene oxide, and the ethylene-oxide opening, the suffixes **–PO2**, and **–EO** will be used, respectively, again in combination with the prefix **1-** (**1-Px-PO2** vs. **1-PxC-PO2**, and **1-Px-EO** vs. **1-PxC-EO** for *x* **=** 1,3).

For the systems that include models for the Co(III)-salen catalyst, the open and cyclic macromolecules with **P1**-length (**P1** and **P1C**) were only studied. Use of the smallest models is justified by the size of the system; also, it will be shown in “Results and discussion” section that the effect of the chain length is much smaller than the influence of the catalyst.

Also, for the systems involving the catalysts, we focus mainly on the species present in the ethylene-oxide copolymerization. For propylene oxide, a variety of possible structures that must be considered in complete, systematic analysis is much larger, due to stereochemistry of both, the catalytic center (due to chirality of two carbon atoms in the cyclohexane ring) and the epoxide. Therefore, the detailed analysis was performed for ethylene oxide. For propylene oxide we have only considered the systems derived from the catalyst with (*S*,*S*) configuration of cyclohexane-carbons, and the *R* configuration on oxirane (*R-*PO); further, only the species resulting from methylene-ring-opening (-**PO1**) were considered in this case. As we focus here on the preference of open vs. cyclic structures, we believe that considering this case is sufficient to demonstrate the qualitative similarity of the results for EO and PO, without necessity of performing detailed analysis for all possible intermediates and pathways resulting from stereochemistry.

For the systems with the model Co(III)-salen catalyst without N^+^-chains, the prefixes **2t-** and **2c-** will be used, for the *trans* and *cis-β* isomers, respectively; examples for propylene oxide (methylene-ring-opening, **-PO1**) are shown in Fig. 5b (**2t-P1-PO1** vs. **2t-P1C-PO1**), and 5**c** (**2c-P1-PO1** vs. **2c-P1C-PO1**).

Finally, for the Co(III)-salen catalyst tethering two-N^+^ chains, the prefixes **3t-** and **3c-** will be used, for the *trans* and *cis-b* isomers, respectively. Examples for propylene oxide are shown in Fig. 5d, (**3t-P1-PO1** vs. **3t-P1C-PO1)** and 5**e** (**3c-P1-PO1** vs. **3c-P1C-PO1)**.

In the case of systems including the catalyst models, **2t-/2c-**, and **3t-/3c-**, besides **P1**/**P1C** anions, one or three additional carbonate species must be present in the respective systems; the simple methyl carbonate anion(s) (CH_3_OCOO^−^) was (were) used here. Different binding modes of considered macromolecules (mono- and bi-dentate complexes) were also studied for *cis-β* isomers (in **2c-** and **3c-** systems); in addition, in the case of real catalysts, different isomers derived from mutual orientation of the two N^+^-chains in **3t-**/**3c-** were considered; we do not introduce additional labels to distinguish between these isomers.

## Computational details

Density Functional Theory (DFT) calculations were carried out using the Amsterdam Density Functional (ADF) package (version 2014.07, 2017.103, 2019.301) [29–31]. Becke-Perdew (BP) exchange-correlation functional [32, 33] was applied with the semiempirical, Grimme’s D3 dispersion correction with Becke-Johnson damping [34]. Use of BP-D3 approach was justified by benchmark calculations presented in our previous paper [25]. Scalar relativistic corrections were applied within the ZORA approximation [35–39]. The Slater-type all-electron TZP basis sets [40] were calculated and used in the calculations for models without catalyst, while the frozen-core (*fc*) approximation was applied in the case of models **2-** and **3-**: for cobalt atom the *fc*-TZP basis set was used (with *1s*-*3p* electron treated as a frozen core), and *fc*-DZP basis—for the remaining elements (with *1s* orbital frozen). Analytical frequencies [41–43] were applied in calculations of Gibbs free-energies, as described in the ADF manual. The reported energy differences correspond to the electronic part, and thus, they do not include differences in zero-point energies (included in the Gibbs free-energy differences). In the present work, we do not consider solvation effects; results of our previous paper [25] indicated that the solvation correction can be used only as a qualitative estimation, as different solvation models provide quantitatively different results. The ETS-NOCV analysis [44–46] was performed using the implementation in the ADF package; details concerning the fragments considered are presented together with the results (see Supporting Information).

To explore the conformational space, the semiempirical, Born-Oppenheimer molecular-dynamics (MD) simulations were performed by locally developed MD driver program, using potential energy and the forces acting on nuclei calculated by single-point PM7 [47] calculations with MOPAC 2016 program [48]. In consistency with our previous work [25], PM7 parametrization was chosen here, as one of the most recent semiempirical approaches, providing quite reasonable representation of non-covalent interactions, important in the systems investigated in the present work. The Verlet-velocity algorithm [49, 50] was used for propagation of nuclei with 1-fs timestep. The temperature of the simulation (*T* = 353 K) was controlled by velocity scaling, initially switched on at every 5 timesteps during the system warm-up, and afterwards—at every 150 timesteps. A similar approach was used in our previous paper [25].

In the case of macromolecules without catalysts **(Px-m**, *x* = 1-6; **P1C-m**, **P3C-m**, **P5C-m** for *m* **= EO**, **PO1**, **PO2**), a set of conformers was first generated by DFT potential-energy surface profiles, obtained by rotating around various single-bonds. The minima located from such potential-energy-surface scans were used as starting points for semiempirical MD simulations (100 ps). A set of geometries selected from each MD trajectory (every 100th geometry, i.e., 1001 geometries in total) was optimized with PM7 method. The resulting geometries were analyzed to select those corresponding to different conformations; this was done by comparison of torsion angles (with resolution of 10^o^). The selected set of geometries was finally optimized at DFT level.

To see the electrostatic effect of the cation in the vicinity of the anions considered above, for the minimum energy structures (open and cyclic), we have performed single-point DFT calculations in the presence of the positive point charge (+1) located in the vicinity of alkoxide oxygen atom, at the extension of CO bond, at the distance of 2 Å (see Fig. S1 in Supporting Information), roughly corresponding to the Co-O bond length.

For systems including quaternary ammonium cation (**1-P1-m**, **1-P1C-m**, **1-P3-m**, **1-P3C-m** for *m* **= EO**, **PO1**, **PO2**), a similar approach was used, utilizing semiempirical MD simulations to select the structures for DFT optimizations. Here, a set of MD simulations was performed (100 ps), starting from low-energy minima, located previously for isolated macromolecules. A set of geometries selected from each MD trajectory (every 100th geometry, i.e., 1001 geometries in total) was pre-optimized with PM7 method. In this case, all the geometries were finally optimized by DFT (since in this case not only the conformation within an anion is important, but as well, the mutual orientation of anion and cation).

A combined (semiempirical MD/DFT geometry optimizations) approach was also applied for the models including catalyst complex (**2t/c-P1-m**, **2t/c-P1C-m**, **3t/c-P1-m**, **3t/c-P1C-m**; for *m* = **EO**, **PO1)**.

Here, however, the complexity becomes larger since different binding modes are theoretically possible. All considered binding modes are depicted in Fig. S2 in Supporting Information. For the systems involving the model catalyst (**2t/2c**), we have considered mono-dentate bonding of the open and cyclic anions to the metal via alkoxide oxygen, for both *trans* and *cis-β* isomers. In addition, for *cis-β* isomer, the bi-dentate bonding was considered (impossible in *trans* coordination). These binding modes are depicted in Fig. S2 with shaded background, as the major structures considered, that are expected to be energetically preferred. Furthermore, the other binding modes shown in Fig. S2 were as well examined, to verify if they indeed represent the higher energy structures. For each of the binding mode, the semiempirical MD simulation (100 ps) was performed. A set of geometries selected from each MD trajectory (every 100th geometry, i.e., 1001 geometries in total) was optimized at PM7 level. Finally, in each case, for DFT optimizations, a set of 50 lowest-PM7-energy structures was selected, and extended by every 10th of the higher energy structures; this gives 145 structures in total, optimized at DFT level for each binding mode.

In the case of the “real” catalyst (**3t/3c**), a variety of structures emerges from the combination of the major (low-energy) binding modes described above (Fig. S2), and the possible mutual orientations of the two N + -chains (e.g., for *trans* isomers: “down-down,” “down-up,” “up-up,” with respect to the salen plane; see Fig. S3 in Supporting Information). For each specific isomer/binding-mode considered, a separate semiempirical MD simulation was run (250 ps). All the initial structures for MD were built by appropriate modification of the low-energy isomers obtained in our previous studies [25] (i.e., by replacement of a carbonate anion, considered in the models studied previously, by open or cyclic alkoxide). A set of geometries selected from each MD trajectory (every 100th geometry, i.e., 2501 geometries in total) was optimized at PM7 level. Finally, in each case, for DFT optimizations, a set of 50 lowest-PM7-energy structures was selected, and extended by every 10th of the higher energy structures; this gives 295 structures in total, optimized at DFT level for each class of structures.

## Results and discussion

In the following, first, the results for isolated ions will be presented, followed by the systems involving the quaternary ammonium cation. Finally, the results for systems involving the model and “real” catalysts will be discussed.

In the first part of Table 1, the energy/free energy differences are collected, each calculated as the difference of the corresponding values for the cyclic intermediate and the open macromolecule (both anionic); in each case, the corresponding Δ*E*/Δ*G* values were calculated based on the energy/free-energy values for the minimum-energy structures, selected from all optimized geometries. The results for the systems **P1C/P1**, **P3C/P3**, and **P5C/P5** show strong preference of cyclic system for ethylene oxide, as well as for both considered pathways of propylene oxide opening. A comparison of the results for different chain lengths (P1, P3, P5) indicates that elongation of the chain affects the observed preference. However, the qualitative picture is the same for P1, P2, P3, indicating strong preference of the cyclic form. The calculated energy- and free-energy differences agree quite well with the results of Darensbourg and Yeung [26–28]; the differences can be attributed to computational details (DFT functional, basis set, solvation, and in particular, dispersion correction).

**Table 1:** The energy/free energy differences for the cyclic intermediate and the open macromolecule; the values in kcal/mol

	EO		PO1		PO2	
	Δ*E*	Δ*G*	Δ*E*	Δ*G*	Δ*E*	Δ*G*
Isolated anions:						
P1C/P1	− 8.34	− 6.17	− 7.02	− 7.60	− 6.99	− 7.52
P3C/P3	− 9.07	− 7.46	− 8.44	− 8.18	− 7.72	− 8.06
P5C/P5	− 9.73	− 8.13	− 10.62	− 11.16	− 9.15	− 10.94
Anions + point charge:						
P1C/P1	5.22	5.66	5.53	6.39	5.69	6.64
P3C/P3	5.21	5.34	3.75	4.55	3.46	4.02
P5C/P5	5.21	5.27	3.04	4.20	3.10	3.90
Anions + TBA cation:						
1-P1C/1-P1	− 3.00	− 2.84	− 5.55	− 5.25	− 4.40	− 4.63
1-P3C-P3	− 6.78	− 5.14	− 9.60	− 11.03	− 9.31	− 10.29

In the first part of Table 2, the corresponding activation barriers are presented for the alkoxide backbiting reaction (open structure ➔ cyclic intermediate). The low barriers in the range of 4–5 kcal/mol at the free-energy level indicate that the cyclization reactions can easily occur. It can be further expected [26–28] that in solution, the cyclization barriers should be even lower. Taking into account the strong preference of the cyclic structures, the opposite reaction is unlikely, being characterized by relatively high barriers (12–16 kcal/mol).

**Table 2:** The activation energies/free energies for the cyclization reactions of isolated anions; the values in kcal/mol

	EO		PO1		PO2	
	Δ*E#*	Δ*G#*	Δ*E#*	Δ*G#*	Δ*E#*	Δ*G#*
P1*➔* P1C	4.61	3.21	4.56	3.49	5.21	4.01
P3*➔* P3C	3.85	2.92	6.52	5.48	7.54	6.26
P5*➔* P5C	5.42	4.21	5.24	4.01	6.89	5.36
1-P3*➔* 1-P3C	3.45	1.65	1.94	0.76	2.48	1.24

**Fig. 6:**
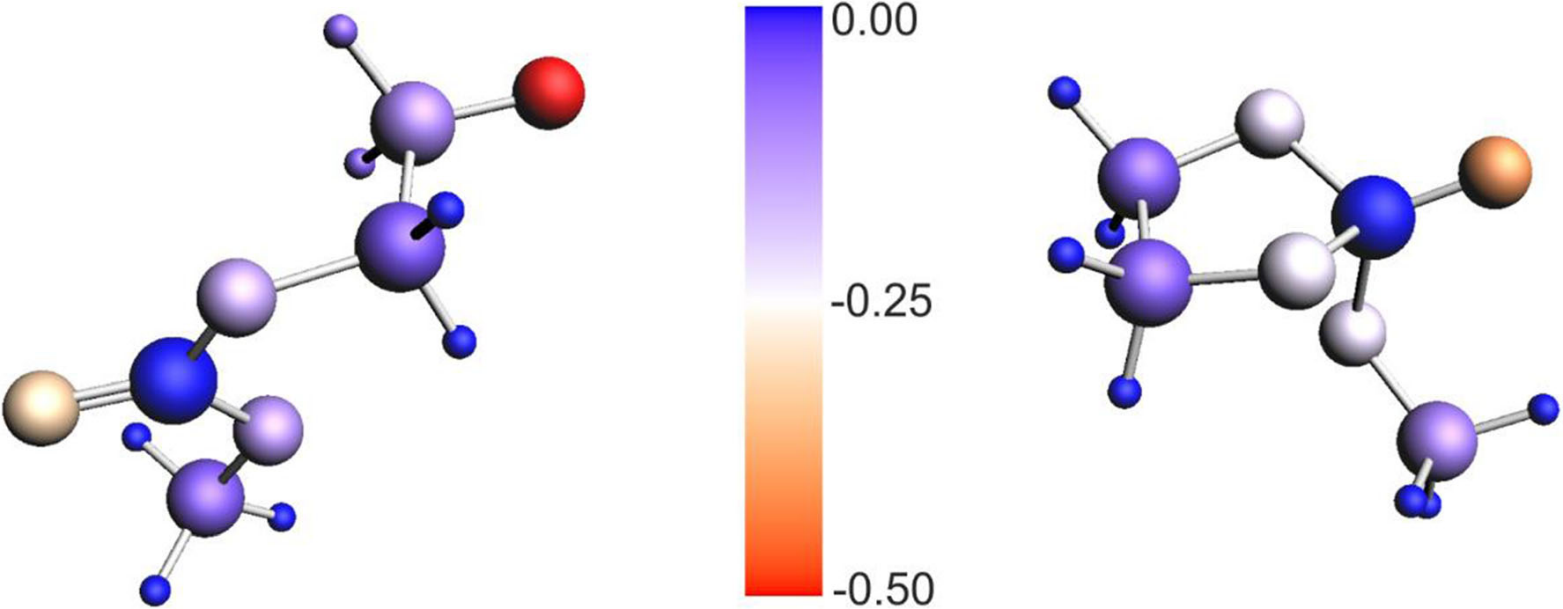
Color representation of Hirschfeld charges in open and cyclic structures, **P1-EO** and **P1C-EO**

To see the electrostatic effect of the cation in the vicinity of the macromolecular anion, we have calculated the corresponding energy/free-energy differences for the cyclic, and open structures in the presence of the positive point charge (+1) located in the vicinity of alkoxide oxygen atom, at the extension of CO bond, at the distance of 2 Å (see Fig. S1 in Supporting Information), roughly corresponding to the Co–O bond length. The results are presented in the second part of Table 1. Here, the preference of the open structures is clear (by 3–5 kcal/mol). The open alkoxide structure is more strongly stabilized by a positive point charge than the corresponding cyclic system, since the negative charge is more delocalized in the latter. This is illustrated by distribution of Hirschfeld charges shown for **P1-EO** and **P1C-EO** in Fig. 6. In the open **P1-EO**, the charge on the alkoxide oxygen is − 0.53, and on the remaining oxygen atoms are − 0.32, − 0.13, and − 0.10, while in the cyclic form, the corresponding values are − 0.42 vs. − 0.31, − 0.31, and − 0.31. The observed qualitative effect of the point charge can be intuitively expected. However, it is not possible to predict intuitively the magnitude of this effect. The results of Table 1 show that in the field of the positive point charge, the open structures are becoming visibly preferred.

In the last part of Table 1, the results obtained for the cyclic and open anions interacting with tetrabutyl ammonium cation are presented. The fact that the effect of ammonium cation is not as strong, as that of point charge is quite intuitive, since the positive charge in the ammonium cation is delocalized, and the distance between the most-negatively charged oxygen of the anion and the nitrogen atom of the cation is much larger, due to the size of the cation. Here, however, for all the systems, the cyclic form is preferred. In the case of ethylene oxide, the preference of the cyclic structure is visibly decreased, compared to that observed for isolated ions. The effect is larger for the short chain (P1; cf. Δ*E* = −8.8 kcal/mol for **P1C-EO** / **P1-EO**, and − 3.0 kcal/mol for **1-P1C-EO**/**1-P1-EO**), and lower for longer chain (P3; cf. Δ*E* = − 9.1 kcal/mol for **P3C-EO**/**P3-EO**, and − 6.8 kcal/mol for **1-P3C-EO**/**1-P3-EO**). In the case of propylene oxide, however, a preference of the cyclic intermediate is much stronger than for ethylene oxide, almost comparable with that observed for isolated ions. This may be explained by the effect of dispersion energy, that is partly counterbalancing the effect of the electrostatic field of cation (see Table S1 in Supporting Information).

Comparing the activation barriers for cyclization, they are slightly decreased in the systems containing the ammonium cation (see Table 2), compared to the isolated anions. It can therefore be concluded, that in the vicinity of the cation, the preference of the cyclic intermediate is somewhat lower, but on the other hand, the cyclization is becoming slightly faster.

**Fig. 7:**
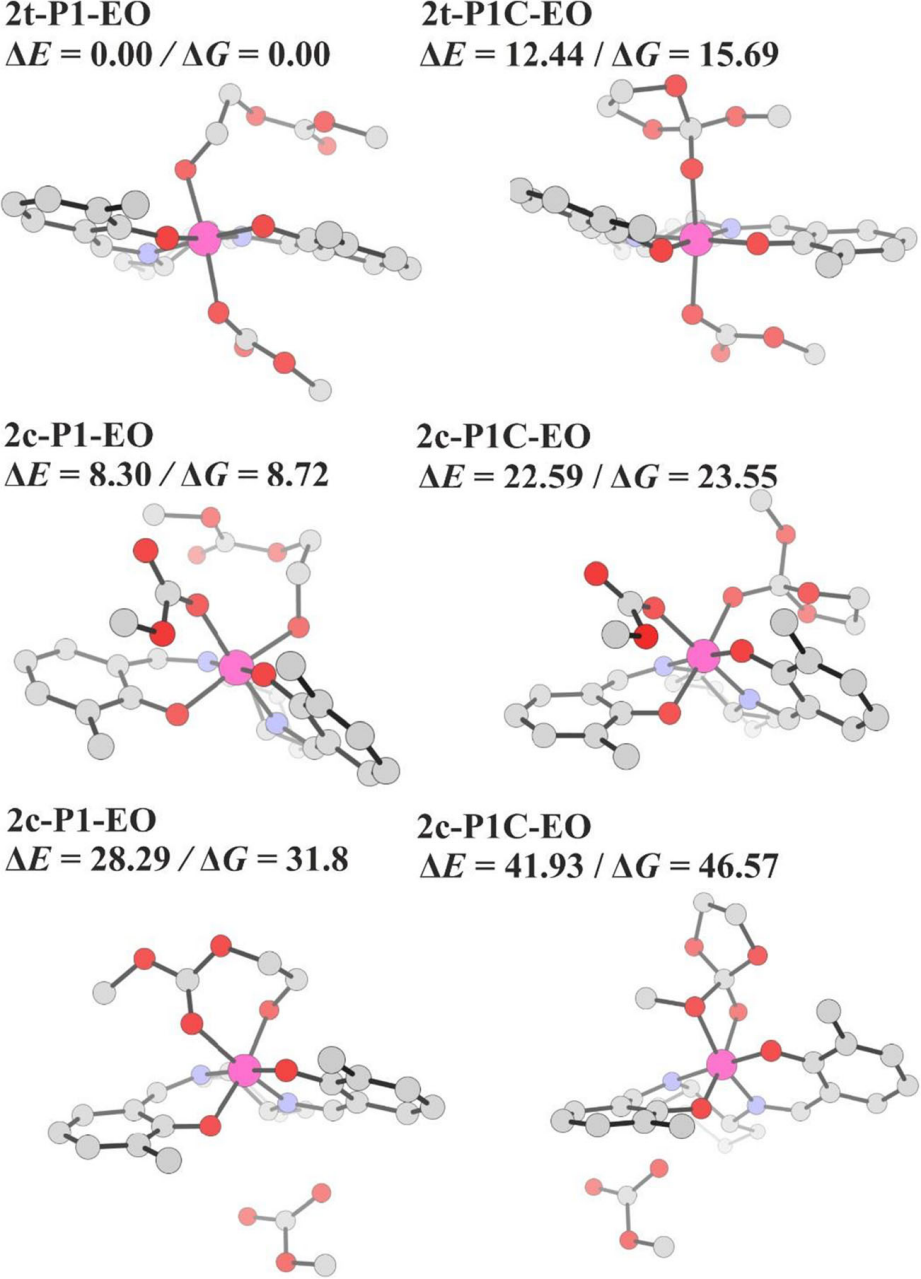
The lowest-energy structures together with the relative energies/free-energies (in kcal/mol) for the *trans* and *cis-β* complexes involving the open chain, and the cyclic intermediate in EO copolymerization. For *cis-β* complexes, the lowest-energy structures from two subsets, with monodentate (middle row) and bidentate bonding (bottom row) are shown. For clarity, the hydrogen atoms are not shown

Let us discuss now the system involving the model Co-salen complex (without N^+^-chains). In Fig. 7, the lowest-energy structures together with the relative energies/free-energies are shown for the *trans* and *cis-β* complexes involving the open chain and the cyclic intermediate in EO copolymerization. Two binding modes were considered for *cis-β* complexes: modentate binding (by alkoxide oxygen atom) and bidentate (by two oxygen atoms); in the figure, the lowest energy systems within both groups are shown. The results show that the most stable is the *trans* complex with open macromolecule bound by the alkoxide-oxygen atom (**2t-P1-EO**). Further, the strong preference of open macromolecules compared to cyclic intermediates is clearly shown for all considered isomers/binding modes. For the lowest-energy *trans* complexes, the energy/free-energy difference between the cyclic and open forms is 12.4/15.7 kcal/mol. Thus, the effect of the metal is much stronger than that of the point charge discussed above. For the corresponding *cis-β* complexes, the difference in the energies/free energies of open and cyclic forms is lower, but all *cis-β* complexes are much less stable than the *trans* isomers. The lowest-energy *cis-β* complex is higher in energy/free energy by 8.3/8.7 kcal/mol. In the case of propylene oxide, the results are qualitatively similar (see Fig. S4 in Supporting Information), indicating strong preference of open systems, compared to the cyclic ones, and the preference of the *trans* isomers, compared to *cis-β*.

**Fig. 8:**
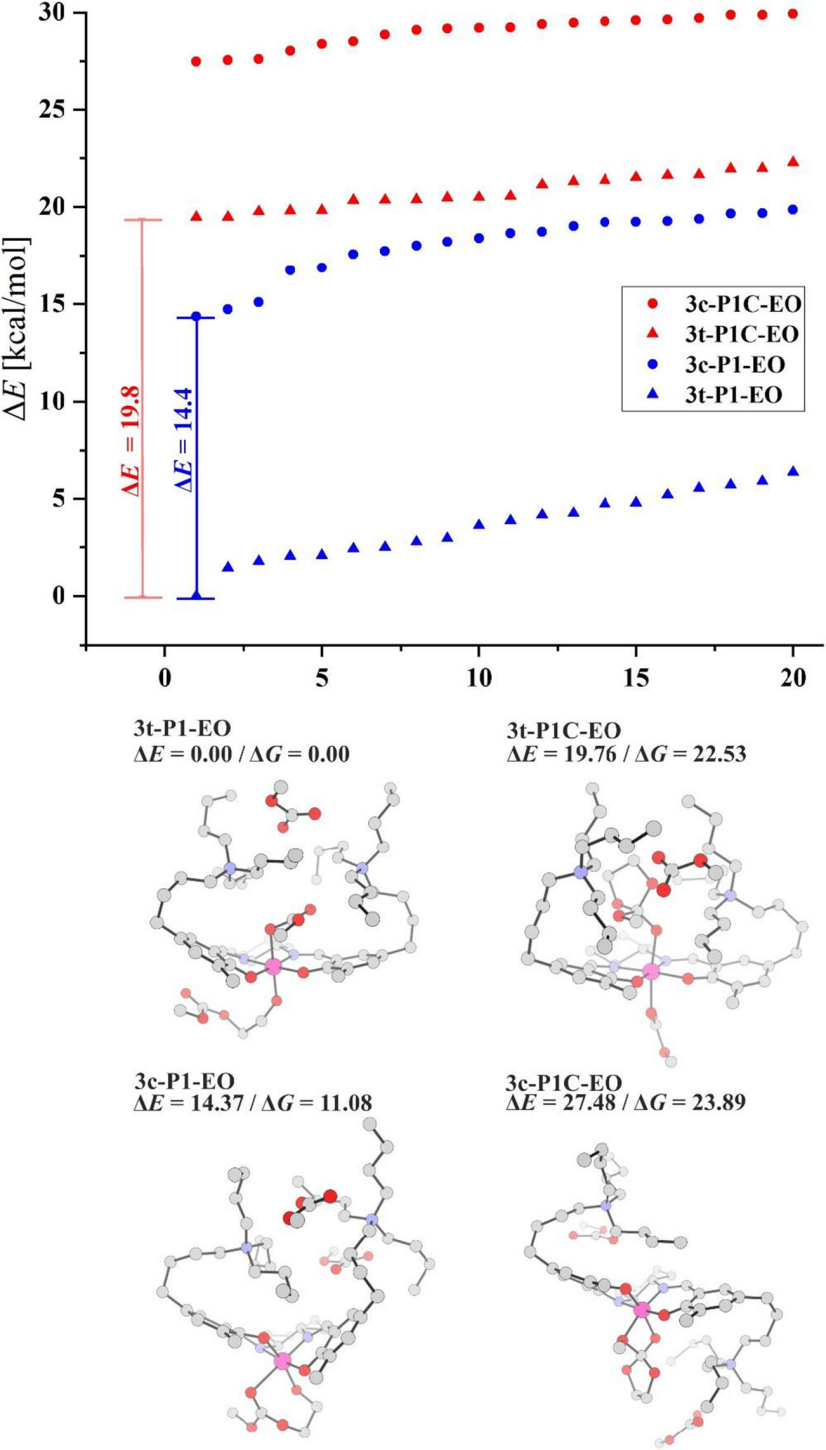
Relative energies of four groups of complexes for the “real” catalyst (*trans/cis-β*; *open/cyclic)* together with the lowest-energy structure within each group, for EO copolymerization. For clarity, the hydrogen atoms are not shown

Let us now briefly discuss the results obtained for the “real” catalyst involving two *N*^+^-chains. In Fig. 8, the energies of 20 lowest energy structures within each of four main categories are presented: open *trans* (**3t-P1-EO**), open *cis-b* (**3c-P1-EO**), cyclic *trans* (**3 t-P1C-EO**), and cyclic *cis-β* (**3c-P1C-EO**) complexes. Similarly to the model catalyst considered above, the results show strong preference of the complexes with open macromolecule. The most stable are the structures from the *trans* family. The *cis-β* complexes are higher in energy by 14–15 kcal/mol. The energies of the *trans* complexes with cyclic intermediate are higher by 18–19 kcal/mol than the *trans* systems with open chain. The highest-energy group is represented by the *cis*-beta complexes with cyclic intermediate. In the bottom part of Fig. 8, the structures are presented, corresponding to the lowest energy within each group. It is worth to point out that the lowest energy structure represents the group of isomers in which both N + -chains are located on the opposite side of the salen-plane than the alkoxide ligand. The lowest-energy structures within other isomers (concerning mutual orientation of N + -chains and the alkoxide) are presented in Supporting Information (Fig. S5). The results for PO are shown in Supporting Information (Fig. S6); the conclusions are qualitatively similar, indicating the preference of the open systems, and the *trans* isomers.

The results presented so far indicate the high instability of the cyclic intermediates coordinated to the metal, compared to the structures with open alkoxide. Also, in the systems with open alkoxide attached to the metal, the nucleophilic oxygen atom is strongly bound to the metal. Therefore, possible cyclization pathway should proceed with dissociation (at least partial) of alkoxide from the metal. On the other hand, it was also shown above that the cyclic forms are strongly preferred in the vicinity of the quaternary ammonium cation. Therefore, we consider here the dissociative pathway (alkoxide transfer to the N^+^-cation) as possible cyclization route.

**Fig. 9:**
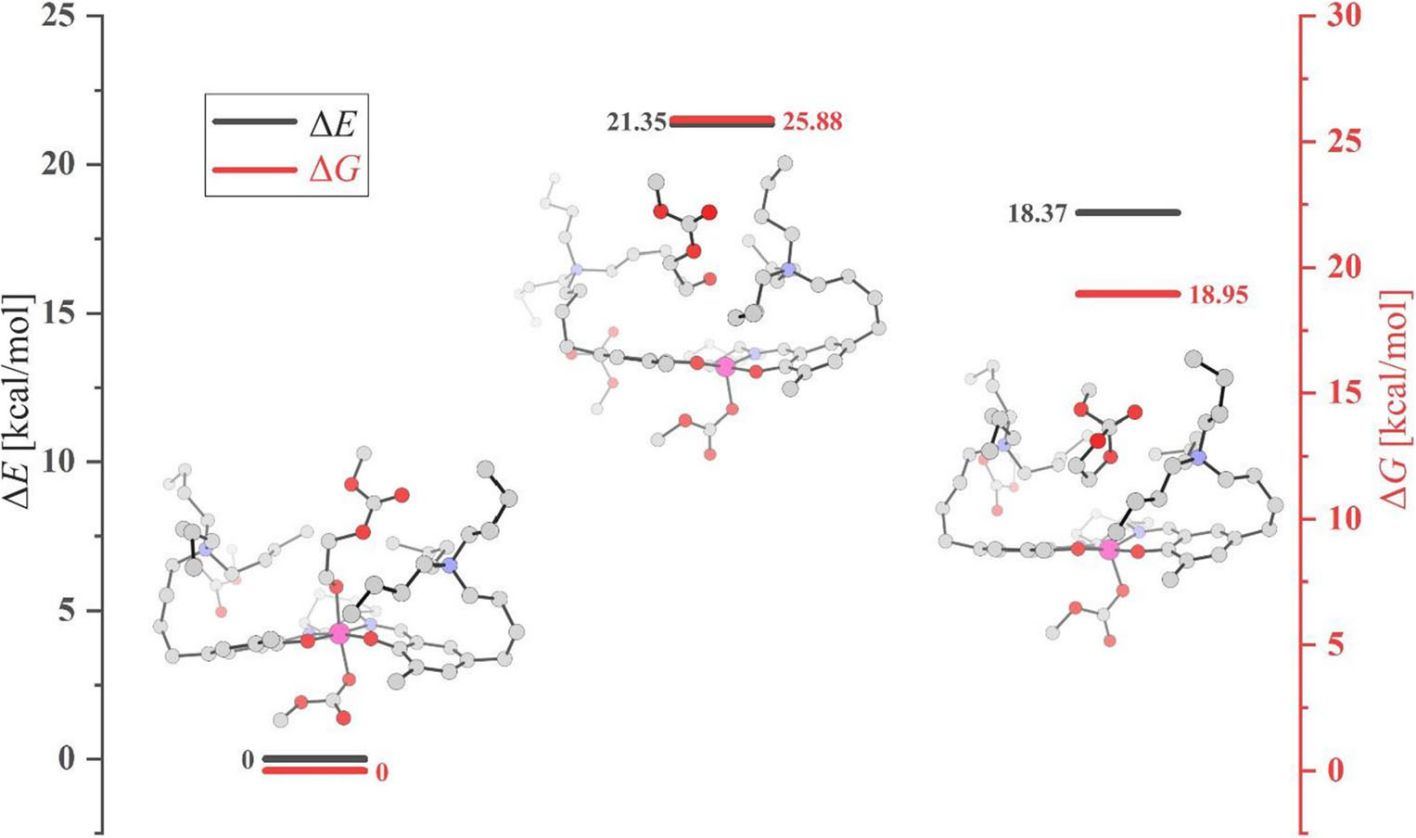
The energy/free-energy profiles for a cyclization pathway, involving dissociation of the alkoxide-ended open chain from the metal, and its transfer to the neighborhood of the ammonium cation. Energy/free-energy values in kcal/mol

**Fig. 10:**
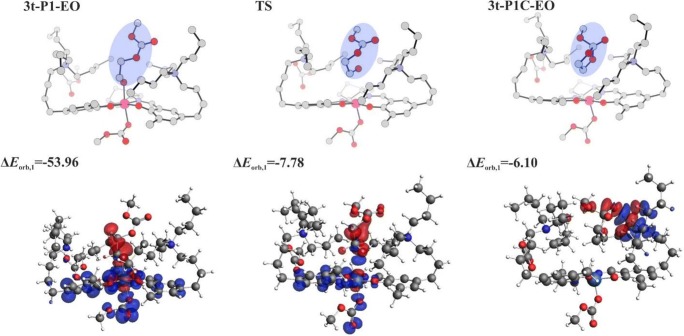
The dominating NOCV-contribution to deformation-density for the initial structure, TS, and the product, corresponding to the dissociation/cyclization pathway presented in Fig. 9

In Fig. 9, the energy and free-energy profiles are shown for such a reaction pathway, involving dissociation of the alkoxide-ended open chain from the metal and its transfer to the neighborhood of the ammonium cation. Along this pathway, after passing the transition state, the cyclization occurs spontaneously. The activation barrier for such alkoxide transfer is relatively high, ΔE#/ΔG# = 21.3/25.9 kcal/mol with respect to the initial structure. Also, the cyclic product attached to ammonium cation is much higher in energy/free energy (18.4/19.0 kcal/mol) than the starting open system. The high barrier originates from breaking the strong alkoxide-metal bond. To illustrate this, the results of the ETS-NOCV analysis are presented in Fig. 10, performed for the three structures shown in Fig. 9 (open structure, TS, cyclic product), concerning the bond between alkoxide fragment and the rest of the complex. In Fig. 10, only the dominating deformation-density NOCV-contribution for each of the three structures is shown; other NOCV-contributions and the ETS interaction-energy components are presented in Supporting Information (Fig. S7). The deformation-density contributions clearly show strong alkoxide-metal bond in the initial, open alkoxide structure (Δ*E*_orb,1_ = − 54.0 kcal/mol). In the TS structure, this bond is practically broken (Δ*E*_orb,1_ = − 7.8 kcal/mol), and in the final structure, the interaction between the cyclic alkoxide and the ammonium cation (Δ*E*_orb,1_ = − 7.8 kcal/mol) is manifested by polarization of these two parts of the system. The total interaction energy is the most stabilizing for the initial complex (− 144.3 kcal/mol), while for TS and the cyclic product, the stabilization is much lower (− 111.1, − 111.3 kcal/mol). The corresponding values for the orbital interaction energy are − 94.0, − 43.2, and − 38.6 kcal/mol.

It should be emphasized that for copolymerization process to proceed, it is required that one of the ligands attached to the metal is transferred to quaternary ammonium cation, to make space for upcoming epoxide, which must be coordinated to the metal prior to its opening. However, a dissociative transfer of the alkoxide is much less probable than the transfer of carbonate anion, since the metal-alkoxide bond is much stronger than the metal-carbonate interaction. This was shown previously by Darensbourg and Yeung [27] for other model catalysts, by considering the ligand dissociation energies. For the complexes investigated in the present work, this is illustrated by the results of ETS-NOCV analysis, presented in Supporting Information for systems **2t-P1-EO** (Fig. S8), and **3t-P1-EO** (Fig. S9), and compared with **1-P1-EO** (Fig. S10). In the case of **2t-P1-EO** model (anionic), the total interaction energy for alkoxide is − 88.6, while for carbonate, − 47.5 kcal/mol. The corresponding orbital interaction energies are − 94.8, and − 54.5 kcal/mol. For **t-P1-EO** (neutral), the total interaction energies are − 144.9, and − 119.1 kcal/mol, for alkoxide and carbonate, respectively. The corresponding orbital interaction energies are − 110.6, and − 71.1 kcal/mol (see Fig. S6). Thus, for both, model (anionic) and real (neutral) catalysts, the total orbital interaction energy is more stabilizing for alkoxide than for carbonate, by ca. 40 kcal/mol. Therefore, it may be concluded that for the bifunctional Co-salen catalysts tethering two-N^+^ cations, cyclization is disfavored. The results presented here justify high selectivity of these catalysts toward polymer formation observed experimentally for the “real” catalysts studied here and related bifunctional catalysts [15–18].

## Concluding remarks

In the present account, the preference of open chain of growing macromolecule vs. possible cyclic form was examined for the bifunctional cobalt(III)-salen catalyst for the copolymerization of CO_2_ copolymerization with epoxides. *Trans* and *cis-β* isomers of the salen-core were considered, as well as, a variety of the structures resulting from different mutual orientation of the N^+^-chains and possible binding modes. To explore the large conformational space in the studied systems, a combined approach was applied, utilizing semiempirical (PM7) MD and the DFT calculations.

Furthermore, the preference of the open and cyclic macromolecules attached to the metal center was compared with the corresponding results for isolated model macromolecules, and the systems built of the macromolecule interacting with the tetra-butyl ammonium cation.

Result shows that the cyclic structures are strongly preferred for isolated ions; for those systems, the cyclization reactions are characterized by low barriers. In the field of positive point charge, the open structures are strongly preferred. However, for the ions interacting with tetrabutyl ammonium cation, the cyclic structures are still strongly preferred, due to delocalization of the positive charge in the cation; the cyclization barriers for the ions in the vicinity of the cation are low. For the complexes involving model and “real” Co(III)-salen catalysts the open structures are strongly preferred. The possible cyclization for the real catalyst by dissociation of alkoxide and its transfer to the neighborhood of quaternary ammonium cation is characterized by high activation barriers. Furthermore, the transfer of alkoxide from the metal center to the cation is less likely than the transfer of carbonate, since the metal-alkoxide bond-energy energy is much stronger than energy of metal-carbonate bonding, as shown by ETS-NOCV results. The conclusions concerning lack of cyclization for the investigated bifunctional catalysts are in qualitative agreement with experimental data showing high selectivity towards copolymer formation in the copolymerization processes catalyzed by those complexes.

## Electronic supplementary material

ESM 1 (PDF 2189 kb)
