# Supplementary material for: Theoretical study on preference of open polymer vs. cyclic products in CO2/epoxide copolymerization with cobalt(III)-salen bifunctional catalysts
Source: J Mol Model. 2020 May 6;26(6):113. doi: 10.1007/s00894-020-04364-x (PMC7203596; doi:10.1007/s00894-020-04364-x)
Supplement: Supplementary file 1 — (PDF 2189 kb) [file 894_2020_4364_MOESM1_ESM.pdf]

Supporting Information

Theoretical study on preference of open-polymer vs. cyclic products in CO_2/epoxide copolymerization with cobalt(III)-salen bifunctional catalysts

Aleksandra Roznowska[1] • Karol Dyduch[1] • Bun Yeoul Lee[2] • Artur Michalak[1]

Corresponding author:
Artur Michalak
michalak@chemia.uj.edu.pl)
ORCID: 0000-0003-1408-5474

[1] Department of Theoretical Chemistry, Faculty of Chemistry, Jagiellonian University, Gronostajowa 2, 30-387 Krakow, Poland

2 Department of Molecular Science and Technology, Ajou University, Suwon, South Korea

Table of contents

Table S1. The difference in dispersion energies for **P1C / P1,** and **1-P1C/1-P1** (cyclic – open); the values in kcal/mol. **page 3**

Figure S1. The position of the positive point charge in the model calculations for open-and cyclic structures. **page 4**

Figure S2. Different binding modes considered for the systems involving model catalyst (**2t-/2c-**); for the 'real catalyst' (**3t-/3c-**) only the major (low-energy) binding modes were considered; indicated here by shaded background. **page 5**

Figure S3. Possible isomers resulting from mutual orientations of the two N^+-chains in the case of 'real' catalyst (**3t-**, **3c-**) **page 6**

Figure S4. The lowest-energy structures together with the relative energies / free-energies (in kcal/mol) for the *trans* and *cis-β* complexes involving the open chain, and the cyclic intermediate in PO copolymerization. For *cis-β* complexes the lowest-energy structures from two subsets, with mono-dentate (middle row) and bi-dentate bonding (bottom row) are shown. For clarity, the hydrogen atoms are not shown. **page 7**

Figure S5. The lowest-energy structures for **3t-P1-EO**, within the three groups of isomers, corresponding to different mutual orientation of the N+-chains and the alkoxide, together with the values of relative energies / free-energies (in kcal/mol). **page 8**

Figure S6. Energies of four groups of complexes for the 'real' catalyst (*trans / cis-β; open / cyclic)* together with the lowest-energy structure within each group, for PO copolymerization. For clarity, the hydrogen atoms are not shown. **page 9**

Figure S7. The ETS interaction-energy components, and the dominating NOCV-contributions to deformation-density for the initial structure, TS, and the product, corresponding to the dissociation / cyclization pathway presented in Figure 9 (main manuscript file). **page 10**

Figure S8. The ETS interaction-energy components, and the dominating NOCV-contributions to deformation-density, for alkoxide bonding for **2t-P1-EO** (**left**), **2t-P1-PO** (**middle**), and carbonate bonding in **2t-P1-EO** (**right**). **page 11**

Figure S9. The ETS interaction-energy components, and the dominating NOCV-contributions to deformation-density in **3t-P1-EO**, describing alkoxide bonding (left) and carbonate bonding (right).

page 12

Figure S10. The ETS interaction-energy components, and the dominating NOCV-contributions to deformation-density in **1-P1-EO**, describing alkoxide bonding (left) and carbonate bonding (right).

page 13

Geometries of the systems 2t-/2c- and 3t/3c presented in the manuscript **pages 14-69**

Table S1. The difference in dispersion energies for **P1C / P1,** and **1-P1C/1-P1** (cyclic – open); the values in kcal/mol.

	EO	PO1	PO2
P1C / P1	-3.13	-3.46	-3.76
1-P1C / 1-P1	-2.35	-5.24	-5.38

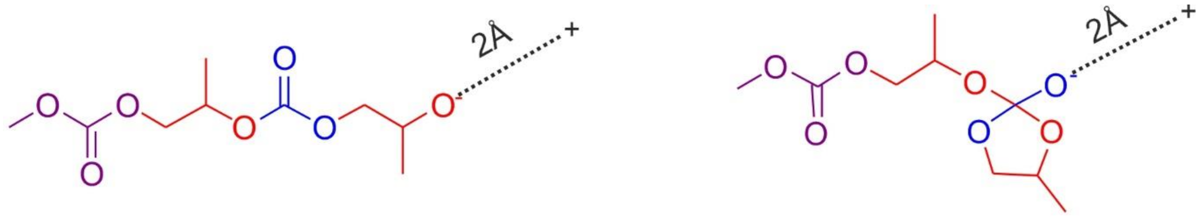

Figure S1. The position of the positive point charge in the model calculations for open-and cyclic structures.

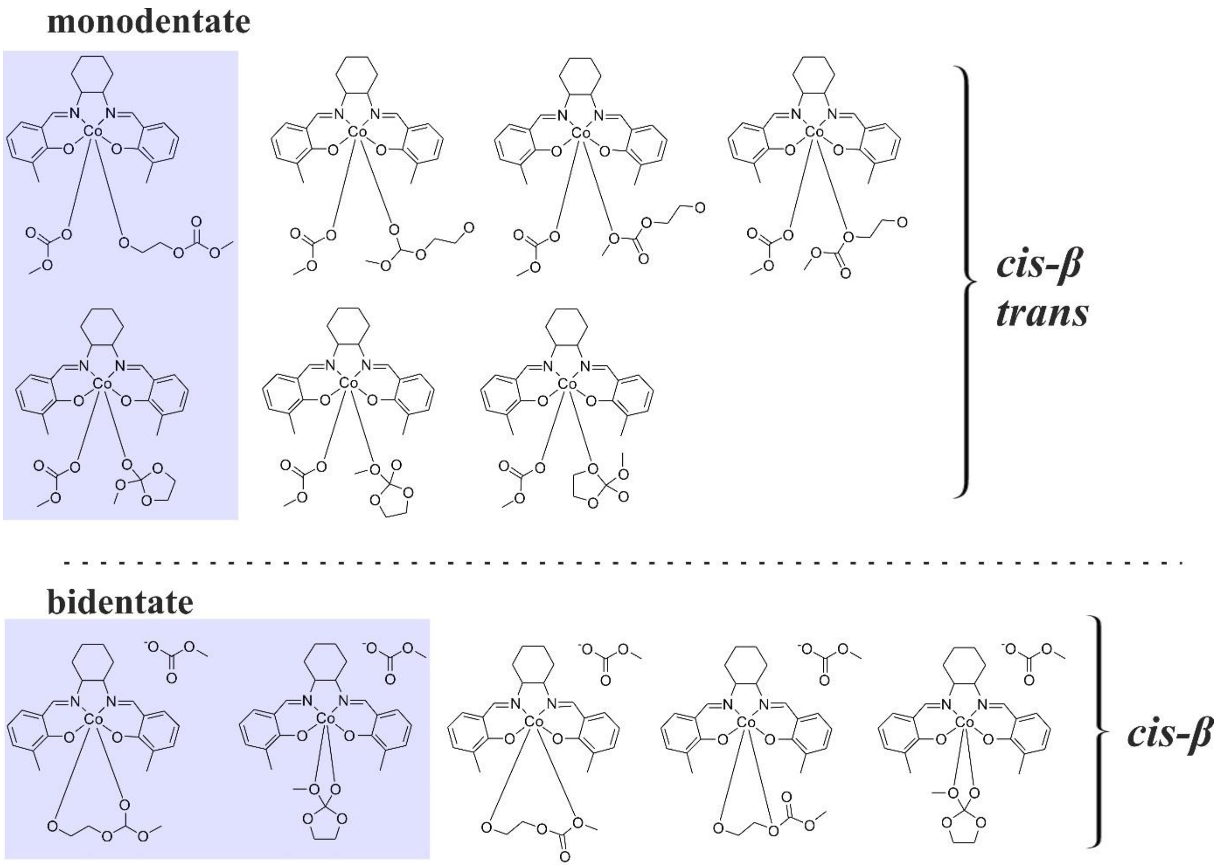

Figure S2. Different binding modes considered for the systems involving model catalyst (**2t-/2c-**); for the 'real catalyst' (**3t-/3c-**) only the major (low-energy) binding modes were considered; indicated here by shaded background.

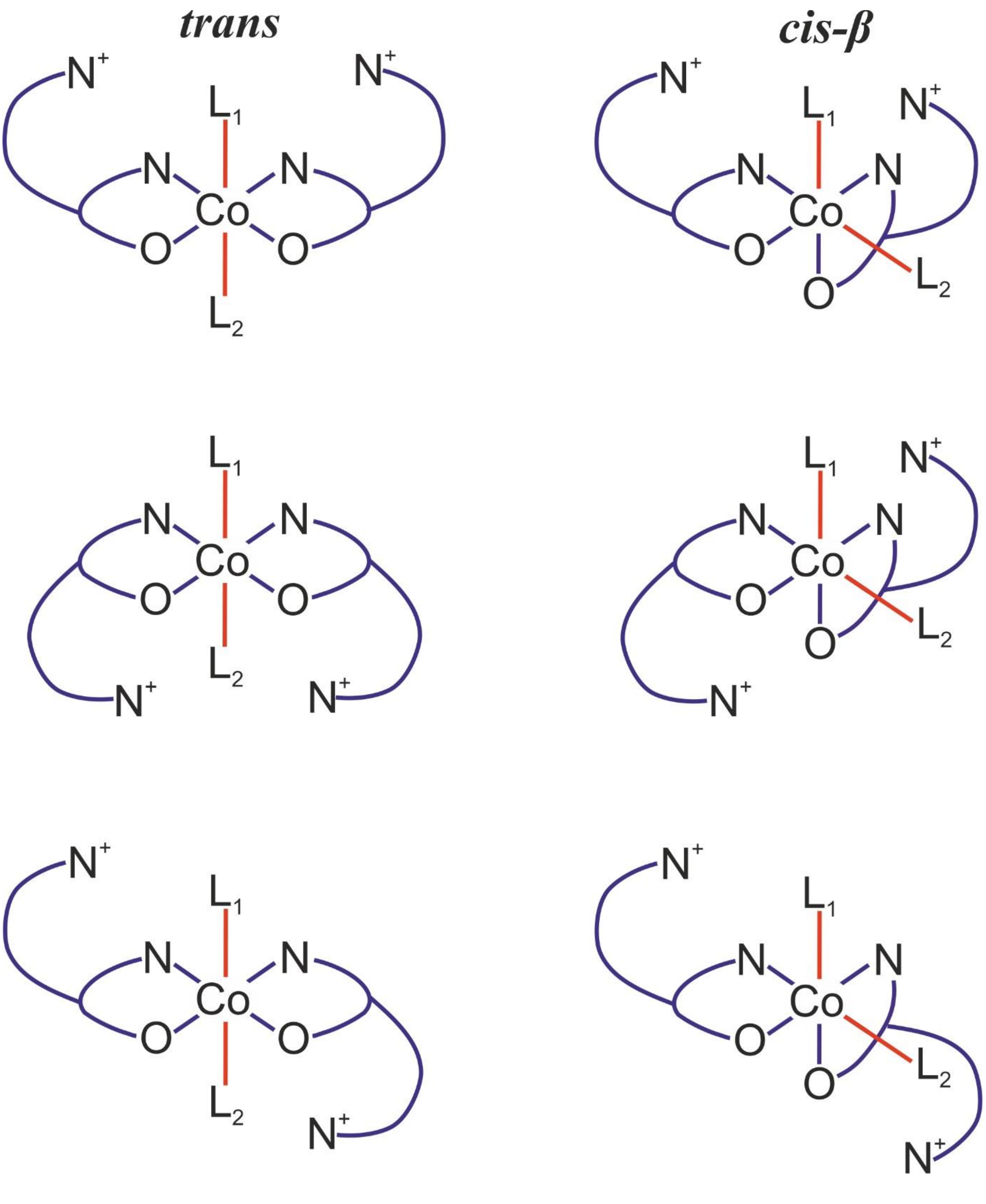

Figure S3. Possible isomers resulting from mutual orientations of the two N^+-chains in the case of 'real' catalyst (**3t-**, **3c-**)

2t-P1-PO1
Δ*E*=0.00 / Δ*G* = 0.00

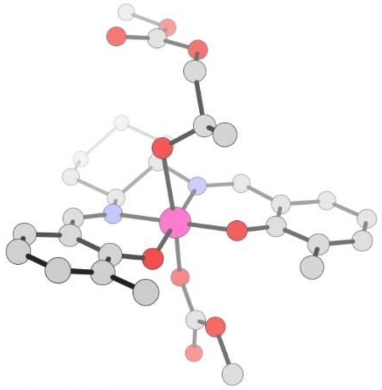

2t-P1C-PO1
Δ*E* = 12.7 / Δ*G* = 17.2

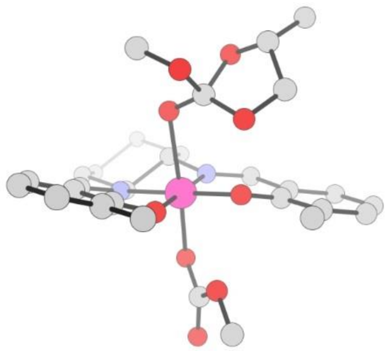

2c-P1-PO1
Δ*E* = 8.51 / Δ*G* = 10.42

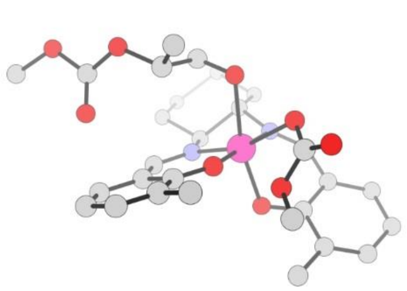

2c-P1C-PO1
Δ*E* = 14.78 / Δ*G* = 18.86

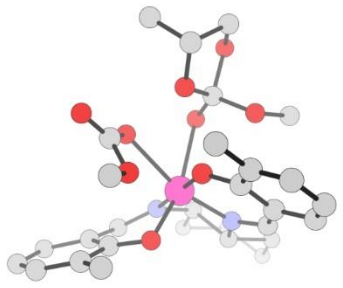

2c-P1-PO1
Δ*E* = 31.95 / Δ*G* = 33.96

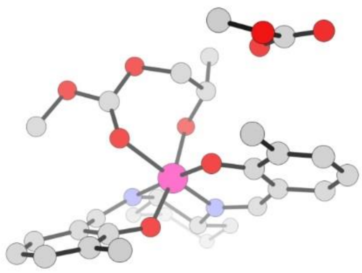

2c-P1C-PO1
Δ*E* = 35.82 / Δ*G* = 38.36

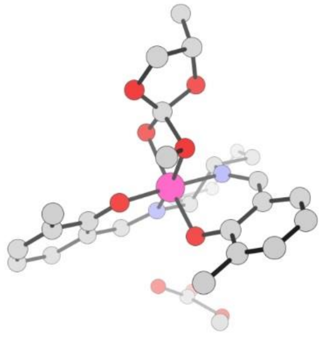

Figure S4. The lowest-energy structures together with the relative energies / free-energies (in kcal/mol) for the *trans* and *cis*-β complexes involving the open chain, and the cyclic intermediate in PO copolymerization. For *cis*-β complexes the lowest-energy structures from two subsets, with mono-dentate (middle row) and bi-dentate bonding (bottom row) are shown. For clarity, the hydrogen atoms are not shown.

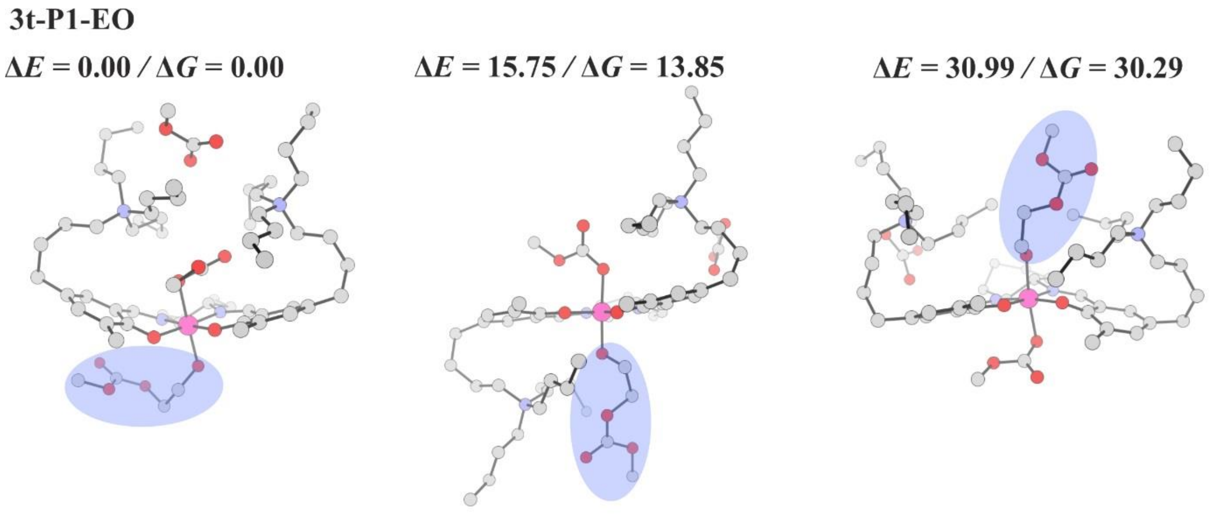

Figure S5. The lowest-energy structures for **3t-P1-EO**, within the three groups of isomers, corresponding to different mutual orientation of the N+-chains and the alkoxide, together with the values of relative energies / free-energies (in kcal/mol).

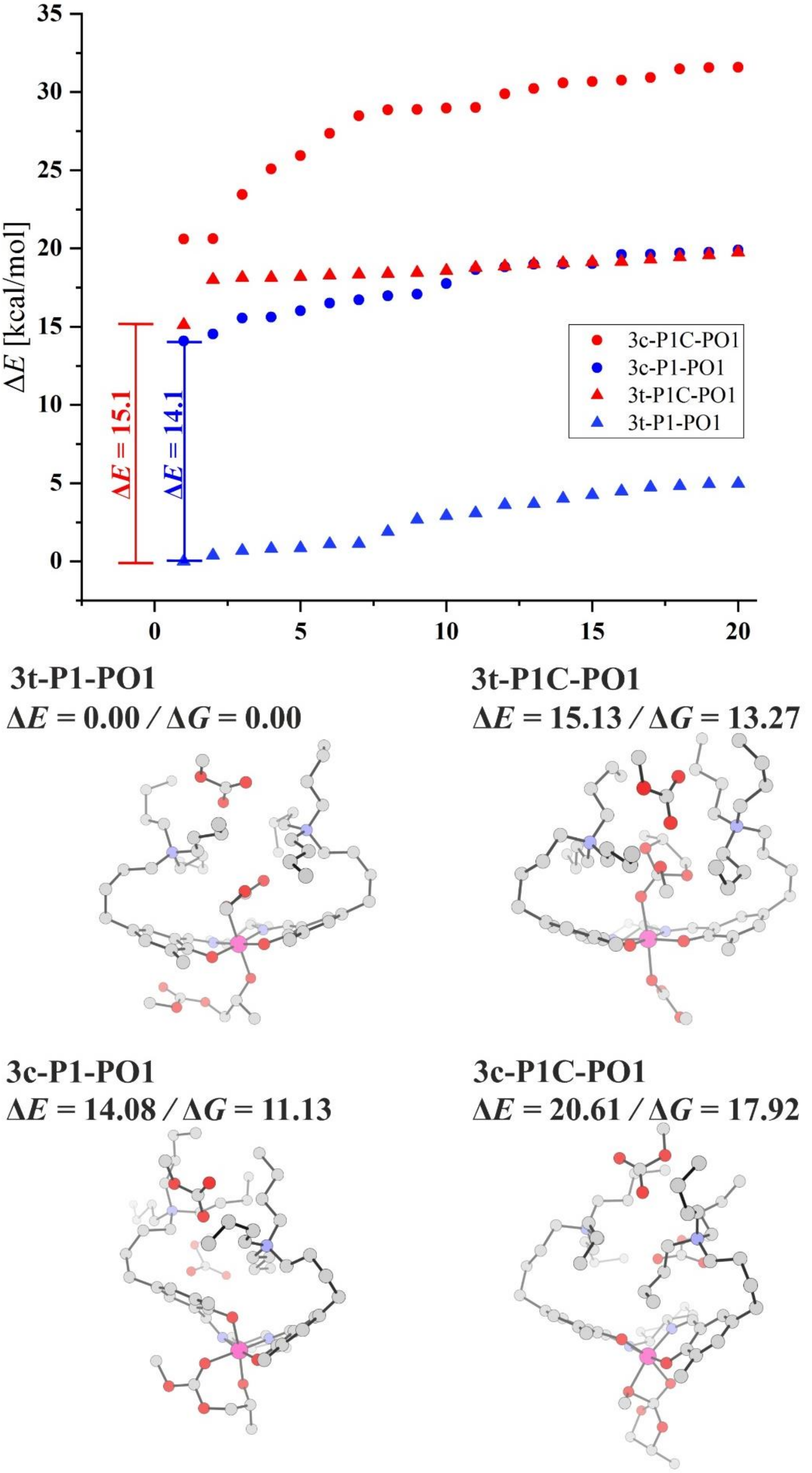

Figure S6. Energies of four groups of complexes for the 'real' catalyst (*trans / cis-β; open / cyclic)* together with the lowest-energy structure within each group, for PO copolymerization. For clarity, the hydrogen atoms are not shown.

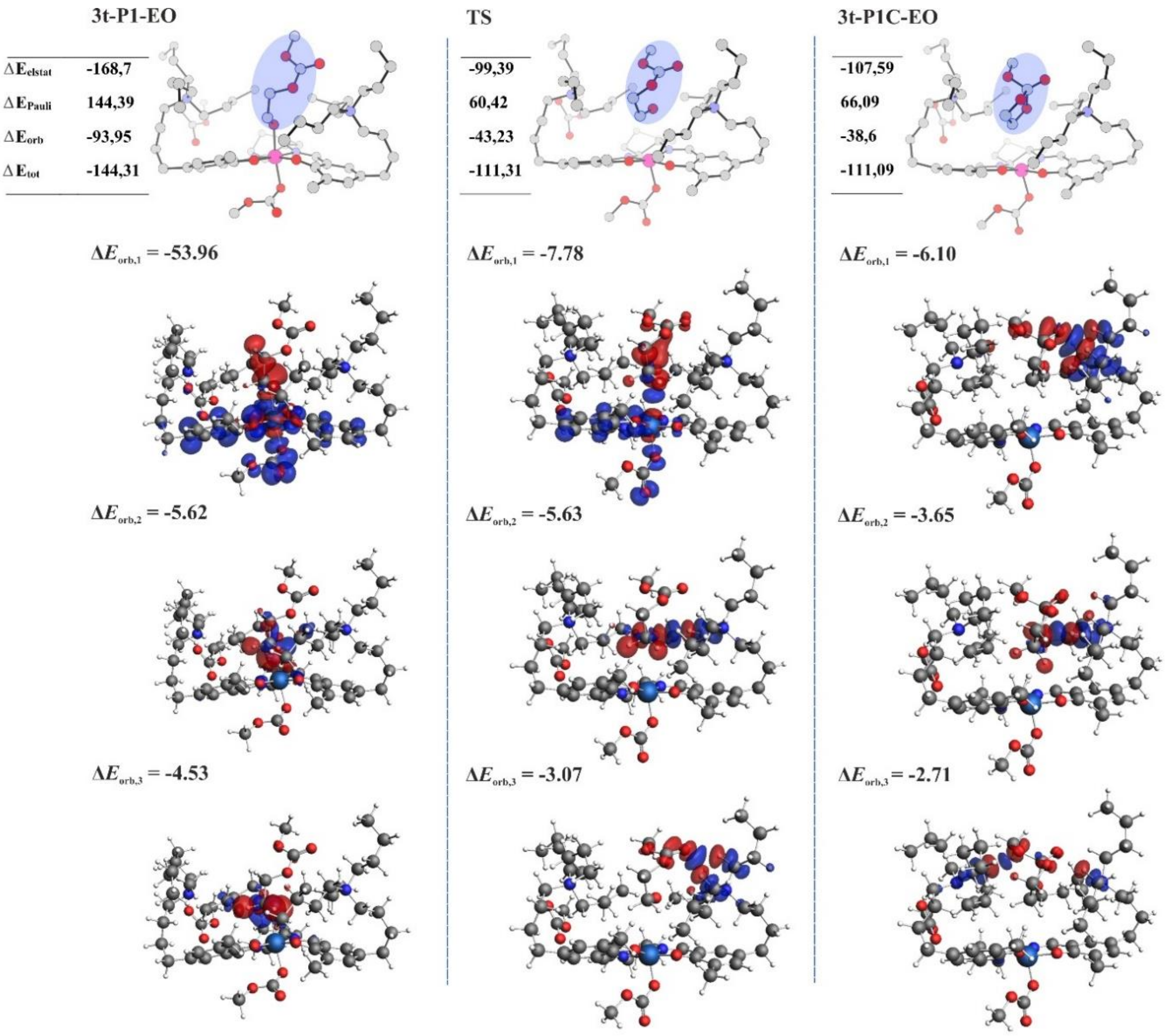

Figure S7. The ETS interaction-energy components, and the dominating NOCV-contributions to deformation-density for the initial structure, TS, and the product, corresponding to the dissociation / cyclization pathway presented in Figure 9 (main manuscript file).

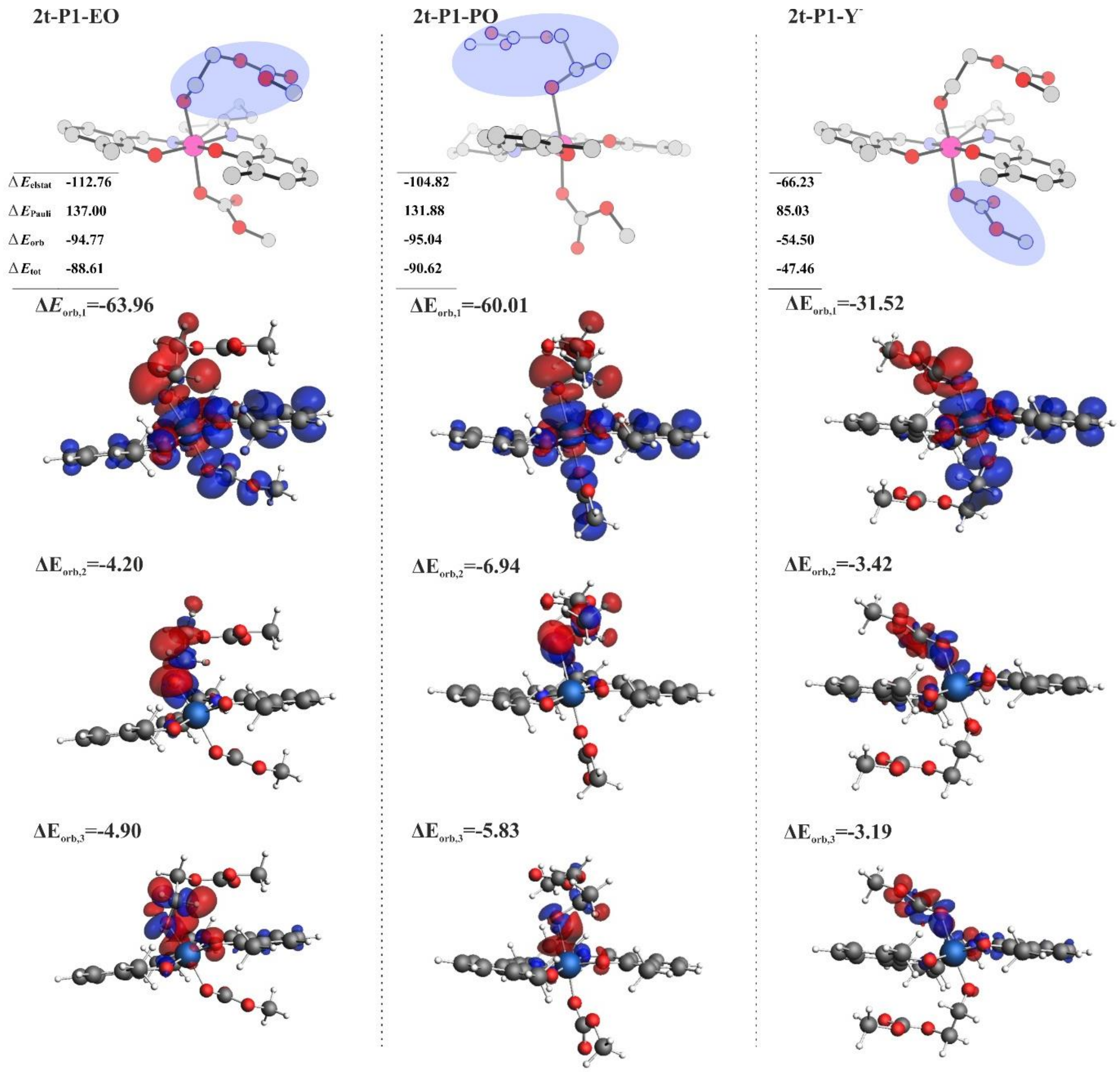

Figure S8. The ETS interaction-energy components, and the dominating NOCV-contributions to deformation-density, for alkoxide bonding for **2t-P1-EO** (**left**), **2t-P1-PO** (**middle**), and carbonate bonding in **2t-P1-EO** (**right**).

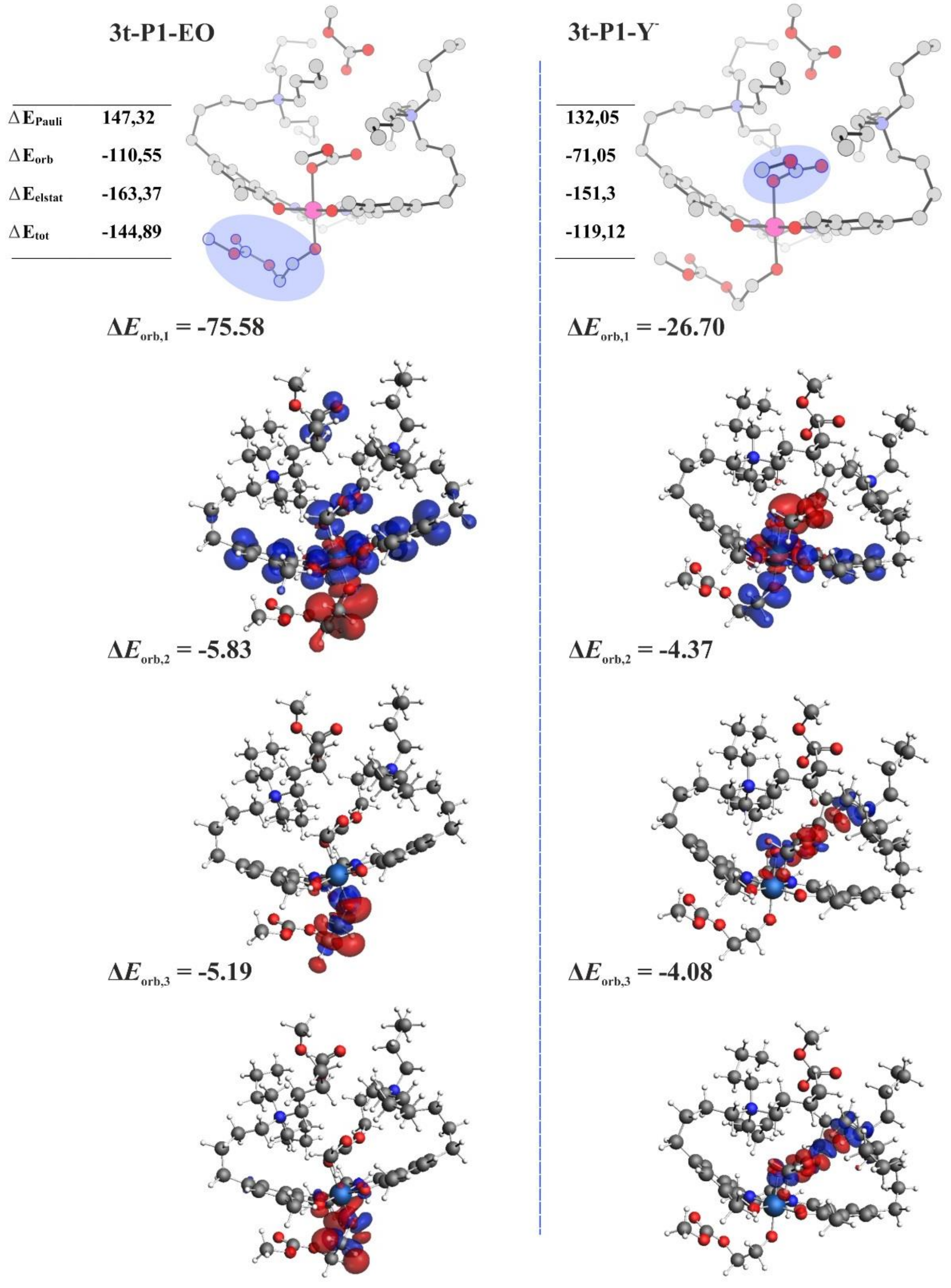

Figure S9. The ETS interaction-energy components, and the dominating NOCV-contributions to deformation-density in **3t-P1-EO**, describing alkoxide bonding (left) and carbonate bonding (right).

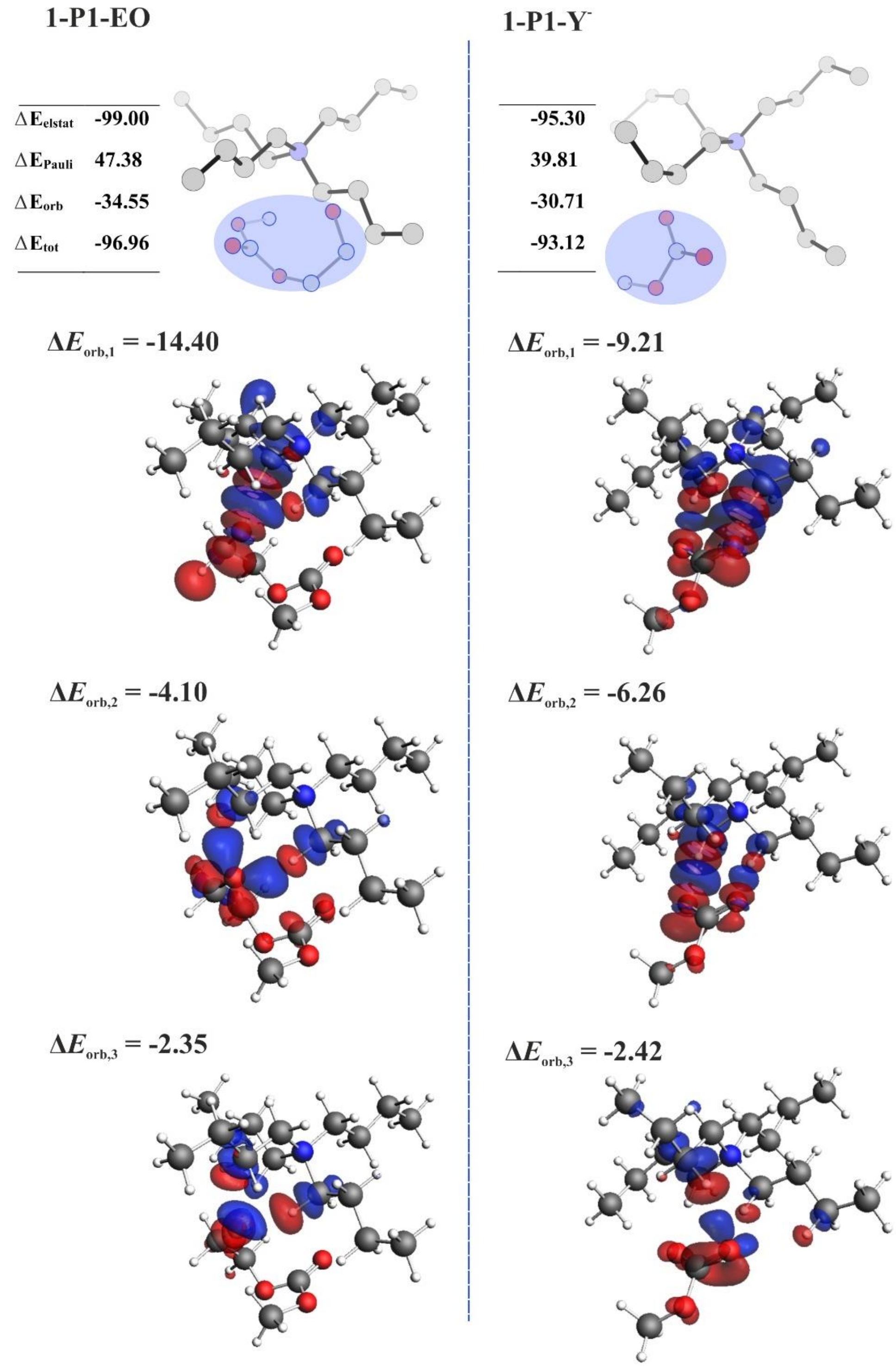

Figure S10. The ETS interaction-energy components, and the dominating NOCV-contributions to deformation-density in **1-P1-EO**, describing alkoxide bonding (left) and carbonate bonding (right).

Geometries of the systems 2t-/2c- and 3t/3c presented in the manuscript

74

2t-P1-EO ΔE=0.00 Fig. 7

C	0.166460	0.638048	-0.037665
C	1.579761	0.496220	-0.257856
C	2.394111	1.644997	-0.364329
C	1.872105	2.917917	-0.234301
C	0.490011	3.061769	0.003415
C	-0.354028	1.970508	0.098847
C	2.209995	-0.784040	-0.370917
N	1.637070	-1.924538	-0.177403
Co	-0.130590	-2.137945	0.471879
O	0.589781	-1.453301	2.116806
C	-1.823032	2.099739	0.364910
O	-0.677650	-0.335405	0.032009
C	2.333937	-3.210028	-0.226014
C	1.846464	-3.970752	1.025886
C	2.427086	-5.375393	1.096977
C	3.957125	-5.307069	1.046397
C	4.437664	-4.571622	-0.208143
C	3.855328	-3.154165	-0.278993
N	0.391798	-3.875827	0.981603
C	-0.394006	-4.884431	1.146292
C	-1.823347	-4.814862	1.114122
C	-2.541495	-6.026422	1.149506
C	-3.923036	-6.046535	1.160063
C	-4.615964	-4.820609	1.148332
C	-3.955554	-3.604271	1.124156
C	-2.522487	-3.563299	1.090410
C	-4.674005	-2.289893	1.090954
O	-1.961762	-2.394192	1.080659
O	-0.813741	-2.713061	-1.334068
C	-0.815717	-3.934338	-1.725911
O	0.106687	-4.763079	-1.793221
O	-2.104838	-4.299859	-2.141339
C	-2.225936	-5.681152	-2.491739
H	-4.468893	-6.990457	1.175756
H	2.515437	3.795446	-0.305159
H	2.146921	-3.379169	1.912111
H	1.917740	-3.756625	-1.096064
H	4.228203	-2.556397	0.573906
H	4.187546	-2.657629	-1.205964
H	4.119178	-5.136582	-1.102956
H	5.539936	-4.524152	-0.229573
H	4.325806	-4.777094	1.944020
H	4.380021	-6.325628	1.078989
H	2.050809	-5.961686	0.239249
H	2.095569	-5.863623	2.027680
H	0.038570	-5.871093	1.330918
H	-5.709955	-4.822154	1.151485
H	-1.974418	-6.960545	1.175539
H	-5.764180	-2.432751	1.073949
H	-4.360025	-1.716909	0.204124
H	-4.402274	-1.671407	1.961725

H	3.274977	-0.769304	-0.631651
H	0.068036	4.063424	0.124646
H	3.464310	1.506414	-0.540596
H	-2.105062	3.148641	0.536314
H	-2.104028	1.492246	1.239713
H	-2.408109	1.698962	-0.478476
H	-3.282065	-5.826721	-2.755330
H	-1.574609	-5.943712	-3.340544
H	-1.965690	-6.320461	-1.635190
C	-0.257591	-1.424216	3.213576
C	0.385193	-2.094765	4.420410
O	0.743502	-3.505528	4.198712
C	-0.209641	-4.447448	4.191392
O	0.060562	-5.625706	4.039049
O	-1.448607	-3.933010	4.383304
C	-2.541701	-4.882140	4.333788
H	-0.257697	-2.026244	5.312556
H	-0.478204	-0.375125	3.538679
H	-2.963438	-4.964174	5.345016
H	-3.281047	-4.478234	3.633546
H	-2.177128	-5.852608	3.985720
H	1.364651	-1.639740	4.623941
H	-1.234418	-1.891052	3.012355

74

2t-P1C-EO ΔE=12.44 Fig. 7

C	0.393709	0.444808	0.124819
C	1.823280	0.302494	0.118768
C	2.648426	1.431225	0.305147
C	2.114511	2.682676	0.546566
C	0.712826	2.824167	0.586702
C	-0.139036	1.753832	0.381078
C	2.452103	-0.968496	-0.083840
N	1.846859	-2.104203	-0.027511
Co	0.002912	-2.340145	0.324776
O	0.158300	-1.640655	2.130373
C	-1.630695	1.876984	0.442579
O	-0.451899	-0.507276	-0.100439
C	2.472179	-3.388628	-0.326641
C	1.896392	-4.399420	0.677854
C	2.313142	-5.812069	0.275652
C	3.845139	-5.900957	0.245643
C	4.456817	-4.849298	-0.684920
C	3.994580	-3.432992	-0.317482
N	0.448003	-4.145743	0.741058
C	-0.380876	-5.066491	1.112960
C	-1.793934	-4.926367	1.234113
C	-2.541067	-6.066367	1.601487
C	-3.918544	-6.030878	1.684076
C	-4.582297	-4.822916	1.381231
C	-3.897172	-3.678669	1.019928
C	-2.461952	-3.687960	0.954528
C	-4.581798	-2.393133	0.671043
O	-1.884707	-2.573517	0.658038
O	-0.111238	-2.805707	-1.631018
C	-0.417669	-4.010187	-1.977287
O	0.318954	-4.844687	-2.523339
O	-1.744589	-4.315231	-1.700602
C	-2.104858	-5.684381	-1.907873
H	-4.486102	-6.917991	1.966200
H	2.762570	3.544614	0.708856
H	2.298531	-4.146429	1.674264

H	2.090011	-3.673306	-1.326688
H	4.360970	-3.153453	0.687422
H	4.406758	-2.713263	-1.043644
H	4.145934	-5.062787	-1.723342
H	5.558431	-4.906479	-0.655509
H	4.232325	-5.746796	1.269749
H	4.155494	-6.912302	-0.068234
H	1.883178	-6.032710	-0.717490
H	1.925899	-6.554131	0.991704
H	0.019419	-6.059157	1.347819
H	-5.674865	-4.786893	1.424707
H	-1.999794	-6.993736	1.809811
H	-5.675298	-2.497376	0.720878
H	-4.285350	-2.073578	-0.340997
H	-4.259810	-1.585524	1.347489
H	3.531274	-0.954047	-0.278580
H	0.279511	3.805977	0.797428
H	3.732915	1.292583	0.276869
H	-1.934399	2.893953	0.731297
H	-2.033617	1.147560	1.163128
H	-2.083075	1.624337	-0.529669
H	-3.083776	-5.808271	-1.427460
H	-2.161854	-5.919840	-2.982429
H	-1.371059	-6.356462	-1.440958
C	0.912107	-2.094515	3.118108
O	0.928154	-1.078043	4.158889
C	1.894021	-0.145748	3.668109
O	2.329987	-2.285029	2.805835
C	3.000305	-1.067462	3.132423
O	0.547181	-3.327917	3.694967
C	-0.852319	-3.374916	4.016796
H	3.764232	-1.281348	3.902342
H	1.465059	0.471850	2.861197
H	-1.446342	-2.896042	3.228489
H	-1.126760	-4.434396	4.089880
H	-1.038071	-2.869292	4.979827
H	3.488006	-0.643991	2.238940
H	2.221407	0.490270	4.502227

74

2c-P1-EO ΔE=8.30 Fig. 7

C	0.275169	-0.229479	1.012318
C	1.643787	-0.107539	0.707018
C	2.194978	1.168205	0.330976
C	1.292814	2.276509	0.234656
C	-0.037934	2.115301	0.582151
C	-0.563091	0.871022	0.980846
C	2.466557	-1.274400	0.577661
N	3.759259	-1.231607	0.615325
C	4.628253	-2.214720	-0.013404
C	5.304664	-1.399699	-1.142116
C	6.370586	-2.244079	-1.836031
C	5.763637	-3.559896	-2.343014
C	5.068285	-4.338312	-1.221429
C	3.989939	-3.487750	-0.541847
N	5.769305	-0.150308	-0.519101
Co	4.781149	0.316131	1.041058
O	6.179022	-0.753646	1.898098
O	3.444246	1.369777	0.040811
C	1.866847	3.588607	-0.204097
C	6.850033	0.455736	-0.889659
C	7.399157	1.636233	-0.299422

C	6.810419	2.285478	0.840804
C	7.446946	3.477119	1.338151
C	8.593984	3.953860	0.735767
C	9.180377	3.304749	-0.373112
C	8.580381	2.166326	-0.870837
O	5.749965	1.901278	1.463154
C	6.804816	4.134589	2.521843
O	3.745149	0.487345	2.708333
C	3.149681	1.466807	3.291430
O	3.230265	2.676003	2.618946
C	2.408233	3.696037	3.193652
O	2.539781	1.385904	4.367022
H	10.090429	3.699128	-0.826421
H	-1.614562	0.778076	1.253866
H	4.506744	-1.122533	-1.861546
H	5.411736	-2.441303	0.727926
H	3.197773	-3.242902	-1.274737
H	3.531409	-4.031127	0.298985
H	5.814633	-4.637712	-0.463466
H	4.624440	-5.266326	-1.620154
H	5.024884	-3.333126	-3.134366
H	6.551861	-4.174640	-2.809739
H	7.174331	-2.448798	-1.106044
H	6.818328	-1.696053	-2.681529
H	7.418189	0.046626	-1.733770
H	9.059789	4.859304	1.135357
H	9.014414	1.648543	-1.730667
H	7.318502	5.069564	2.786497
H	6.813435	3.454428	3.389052
H	5.741921	4.334696	2.318229
H	1.975084	-2.231398	0.366021
H	-0.696760	2.987951	0.548531
H	-0.113332	-1.212828	1.289751
H	1.163332	4.413848	-0.021032
H	2.113414	3.566025	-1.279273
H	2.813825	3.768815	0.325706
H	2.631402	4.610408	2.629309
H	1.343209	3.438509	3.083380
H	2.633532	3.835339	4.260882
C	6.103911	-0.950246	3.270362
C	6.150870	-2.431924	3.647797
O	5.159409	-3.285359	2.970925
C	3.843625	-3.104996	3.195835
O	3.005678	-3.745605	2.580655
O	3.597373	-2.193936	4.149122
C	2.208293	-1.778571	4.253527
H	6.043870	-2.558052	4.738606
H	6.980322	-0.496265	3.799945
H	1.647890	-2.523831	4.836232
H	2.239819	-0.791245	4.727724
H	1.780189	-1.678699	3.251518
H	7.095296	-2.884475	3.311096
H	5.203875	-0.499422	3.711701

74

2c-P1C-EO ΔE=22.59 Fig. 7

C	0.231027	-0.441118	0.546777
C	1.646440	-0.438492	0.297832
C	2.381068	0.763787	0.425983
C	1.776893	1.942926	0.809207
C	0.390681	1.938115	1.083726
C	-0.374176	0.795189	0.967783

C	2.370377	-1.630422	-0.001274
N	1.872205	-2.822782	-0.063649
Co	0.008590	-3.236772	0.062150
O	-1.800943	-3.959717	0.281662
C	-2.946203	-3.574028	-0.163974
O	-4.003996	-4.197808	-0.036448
C	-1.834576	0.744602	1.294315
O	-0.553184	-1.455305	0.450803
C	2.712594	-3.997869	-0.349135
C	1.897721	-5.230705	0.106556
C	2.607104	-6.522545	-0.266547
C	3.986866	-6.542895	0.404296
C	4.817827	-5.321365	-0.004476
C	4.091554	-4.006202	0.307185
N	0.587196	-4.980660	-0.468846
C	0.070716	-5.649412	-1.447571
C	-0.911279	-5.099137	-2.337404
C	-0.934359	-3.676086	-2.527391
C	-1.739923	-3.152019	-3.586504
C	-2.518670	-4.012124	-4.342950
C	-2.516967	-5.404228	-4.132346
C	-1.698789	-5.934850	-3.151481
O	-0.233444	-2.834819	-1.823789
C	-1.723242	-1.668498	-3.792528
O	0.203933	-3.791484	1.949297
O	-2.921216	-2.348211	-0.827455
C	-4.172300	-2.011625	-1.431616
H	2.356275	2.861608	0.907100
H	-3.154737	-6.052750	-4.734299
H	2.807473	-4.070357	-1.453264
H	1.773416	-5.152663	1.199287
H	2.728900	-6.588312	-1.364140
H	2.004454	-7.387345	0.056831
H	3.851479	-6.538960	1.500387
H	4.521744	-7.471955	0.144559
H	5.027440	-5.375685	-1.089579
H	5.793266	-5.335866	0.510611
H	3.936958	-3.893238	1.392315
H	4.700435	-3.158679	-0.046069
H	3.450991	-1.505896	-0.151111
H	-0.093690	2.862582	1.410397
H	3.456380	0.735738	0.228006
H	-2.211841	1.732018	1.596885
H	-2.006835	0.016832	2.103329
H	-2.410602	0.370569	0.434143
H	0.464032	-6.647918	-1.677916
H	-3.162777	-3.588689	-5.118850
H	-1.669908	-7.013462	-2.978903
H	-2.550594	-1.346650	-4.440997
H	-0.771316	-1.346916	-4.247881
H	-1.785368	-1.164108	-2.817543
H	-4.054656	-0.988015	-1.811249
H	-4.396723	-2.697157	-2.265031
H	-4.994289	-2.061788	-0.702293
C	0.821850	-3.162006	2.906850
O	0.555645	-1.749771	2.952316
C	1.637834	-1.140874	3.636609
O	2.294803	-3.259202	2.866656
C	2.847143	-2.002991	3.239627
O	0.515296	-3.748100	4.188750
C	-0.900163	-3.761005	4.425522

H	3.559642	-2.130848	4.073170
H	1.720258	-0.101762	3.284685
H	-1.431425	-4.236628	3.588529
H	-1.052118	-4.335308	5.349794
H	-1.286321	-2.736803	4.559466
H	3.378628	-1.562598	2.375529
H	1.475482	-1.162488	4.731303

74

2c-P1-EO ΔE=28.29 Fig. 7

Co	0.118849	0.080527	0.205846
O	2.362878	0.058064	0.036641
C	3.031629	1.093943	0.044829
O	3.925069	1.407963	-0.904067
C	3.935912	0.491228	-2.034647
N	-0.053740	0.536080	-1.660557
C	-1.143537	1.499519	-1.784436
C	-2.303394	0.801326	-1.039270
C	-3.508731	1.729821	-0.933838
C	-3.917510	2.226650	-2.328035
C	-2.752603	2.885372	-3.072200
C	-1.562883	1.924156	-3.182195
O	0.276801	1.971857	0.598712
C	0.786867	2.341705	1.841371
C	2.180469	1.787092	2.131367
O	3.067371	2.029984	0.987618
N	-1.737512	0.326940	0.245690
C	-2.438908	0.306887	1.337730
C	-1.998819	-0.063099	2.641655
C	-2.944948	-0.011882	3.693919
C	-2.603928	-0.374936	4.978322
C	-1.291616	-0.817465	5.236547
C	-0.327574	-0.884243	4.247801
C	-0.655809	-0.481958	2.912330
O	0.251898	-1.847758	-0.308440
C	0.999368	-2.165195	-1.325327
C	1.123066	-1.380871	-2.522430
C	1.892901	-1.852450	-3.607783
C	2.593881	-3.036596	-3.517932
C	2.504536	-3.794717	-2.332634
C	1.724736	-3.399049	-1.260049
C	0.388713	-0.165500	-2.659040
C	1.619774	-4.194416	0.004183
O	0.294947	-0.516525	2.028429
C	1.066913	-1.365819	4.512279
H	-3.337577	-0.331958	5.783011
H	3.207066	-3.385664	-4.348236
H	-2.563332	-0.104178	-1.622851
H	-0.847953	2.375208	-1.182283
H	-1.861010	1.041905	-3.777481
H	-0.715942	2.407480	-3.697413
H	-2.435930	3.795734	-2.532690
H	-3.074694	3.202487	-4.077154
H	-4.287836	1.371025	-2.921521
H	-4.758239	2.932269	-2.230461
H	-3.237429	2.581862	-0.285481
H	-4.364707	1.213294	-0.471293
H	-3.490735	0.597824	1.262636
H	-1.021427	-1.120791	6.251297
H	-3.960199	0.320352	3.467159
H	1.171868	-1.722640	5.545543
H	1.803365	-0.565601	4.343068

H	1.326518	-2.181185	3.820603
H	0.164378	0.176727	-3.676794
H	3.068684	-4.727246	-2.253804
H	1.944697	-1.248339	-4.517021
H	2.270334	-5.078852	-0.026076
H	0.580900	-4.517624	0.174397
H	1.890441	-3.567255	0.867743
H	0.844483	3.450581	1.833382
H	2.147958	0.708442	2.322270
H	3.860780	-0.547350	-1.695996
H	3.086959	0.723843	-2.687383
H	4.884612	0.680436	-2.546107
H	0.138045	2.058361	2.699055
H	2.660852	2.314231	2.967507
C	-4.281506	-3.642651	0.906166
O	-4.116107	-3.318108	-0.459114
C	-3.877007	-1.969958	-0.740220
O	-3.994418	-1.151137	0.193107
O	-3.560275	-1.755701	-1.914591
H	-4.856653	-4.574101	0.887151
H	-3.292712	-3.797777	1.347435
H	-4.819661	-2.864838	1.458396

74

2c-P1C-EO ΔE=41.93 Fig. 7

C	0.055822	2.640546	0.937056
C	1.483912	2.607542	1.069455
C	2.216975	3.808269	1.183767
C	1.580464	5.031125	1.131334
C	0.178431	5.068032	0.993340
C	-0.587837	3.917816	0.909357
C	2.152020	1.351224	1.177340
N	1.653002	0.238092	0.737405
C	2.068730	-1.067258	1.241489
C	0.871256	-1.488781	2.124753
C	1.061547	-2.913955	2.639331
C	2.406780	-3.046289	3.366757
C	3.580900	-2.590877	2.495823
C	3.376540	-1.149318	2.014120
N	-0.332255	-1.250619	1.306623
Co	-0.079091	0.036235	-0.070025
O	-0.709888	1.584782	0.870275
C	-2.078366	3.939651	0.763643
C	-1.383782	-2.006580	1.398943
C	-2.556542	-1.943111	0.598533
C	-3.594645	-2.871081	0.869792
C	-4.743716	-2.896057	0.113511
C	-4.878813	-1.985662	-0.956941
C	-3.898707	-1.062087	-1.265499
C	-2.701129	-1.008912	-0.477730
O	-1.831450	-0.107070	-0.804740
C	-4.007631	-0.104092	-2.411116
O	0.587102	-1.339473	-1.292119
H	-5.536038	-3.613357	0.327915
H	2.149908	5.957926	1.197984
H	0.836385	-0.777587	2.974913
H	2.097521	-1.741964	0.369538
H	3.345683	-0.477478	2.890808
H	4.213467	-0.827936	1.372429
H	3.672677	-3.257911	1.620089
H	4.524681	-2.668355	3.059074
H	2.381144	-2.436086	4.287743

H	2.548615	-4.091933	3.683549
H	1.019791	-3.604545	1.778214
H	0.249543	-3.191903	3.329579
H	-1.376737	-2.796411	2.157512
H	-5.783178	-2.014086	-1.569471
H	-3.462510	-3.574268	1.695306
H	-4.933584	-0.268472	-2.979360
H	-3.137247	-0.219687	-3.075624
H	-3.985422	0.936744	-2.049831
H	3.120965	1.332147	1.690496
H	-0.327097	6.035830	0.948069
H	3.302342	3.752450	1.294611
H	-2.450529	4.965034	0.638255
H	-2.560519	3.489273	1.646100
H	-2.383664	3.326106	-0.097462
C	0.574227	-0.650393	-2.388621
O	-0.484701	-0.886802	-3.265821
C	-0.084582	-0.304804	-4.513804
O	1.764134	-0.635995	-3.129345
C	1.418136	-0.583107	-4.532873
O	0.299249	0.890211	-1.863416
C	1.394410	1.797078	-2.044065
H	2.008556	0.205517	-5.019604
H	-0.639225	-0.801110	-5.319395
H	1.542257	1.979728	-3.119242
H	1.116436	2.739231	-1.555546
H	2.330525	1.411402	-1.616231
H	1.654747	-1.561859	-4.979950
H	-0.309999	0.775287	-4.507023
C	-0.016873	2.196514	5.067966
O	1.396826	2.129805	5.151679
C	2.024649	1.586222	4.037451
O	1.284226	1.067357	3.159500
O	3.250099	1.671276	4.010553
H	-0.338717	2.562534	4.086230
H	-0.444964	1.207247	5.248952
H	-0.284732	2.905878	5.858632

184

3t-P1-EO ΔE=0.00 Fig. 8, SI

C	-1.571397	1.014609	-3.469323
C	-0.399866	1.309627	-2.521999
C	-0.459502	2.746406	-2.015353
C	-1.826999	3.014105	-1.372024
C	-2.979105	2.720180	-2.337731
C	-2.930100	1.272222	-2.839094
N	0.809209	0.917348	-3.255743
Co	0.488636	-0.474020	-4.516029
O	0.766266	-1.972847	-3.128548
C	1.961874	-2.013387	-2.668534
O	2.788930	-3.027663	-3.155384
C	2.292865	-3.745664	-4.302863
N	-1.310621	-0.326111	-3.978325
C	-2.168629	-1.288043	-3.969206
C	-1.929569	-2.580028	-4.542374
C	-2.868796	-3.593995	-4.277079
C	-2.749778	-4.863137	-4.816414
C	-1.684442	-5.088217	-5.711675
C	-0.758490	-4.112777	-6.045354
C	-0.827811	-2.819824	-5.428548
C	-3.658558	-5.984250	-4.397046
C	-3.038966	-6.890375	-3.314314

C	-2.616604	-6.138510	-2.034645
C	-1.150621	-5.728489	-2.104826
N	-0.631728	-4.841027	-0.987163
C	0.882177	-4.695353	-1.169285
C	1.685624	-5.986591	-1.154704
C	3.186545	-5.660386	-1.123827
C	4.042825	-6.909120	-1.319519
C	0.314766	-4.376560	-7.064093
O	0.075102	-1.937211	-5.737944
C	1.951432	1.471060	-3.036478
C	3.204346	1.056839	-3.580323
C	4.348921	1.682010	-3.050816
C	5.627405	1.230468	-3.316317
C	5.755462	0.141935	-4.201460
C	4.668384	-0.480750	-4.792785
C	3.338046	-0.042285	-4.489297
C	6.821589	1.845344	-2.634412
C	6.700178	1.906714	-1.104200
C	6.626478	0.538644	-0.400467
C	5.221037	-0.064225	-0.364814
N	5.089331	-1.407880	0.377114
C	6.128464	-1.544801	1.466499
C	6.105219	-2.859208	2.230582
C	6.884873	-2.726455	3.547249
C	6.161480	-1.879462	4.597577
C	4.834935	-1.646145	-5.719431
O	2.348375	-0.694979	-5.022441
O	0.263737	0.937769	-5.776371
O	2.473918	-1.300990	-1.796073
C	3.675323	-1.503821	0.958503
C	3.339152	-0.531579	2.080866
C	1.844070	-0.171099	2.005818
C	1.507028	0.732169	0.812926
C	5.152658	-2.551881	-0.625795
C	6.361426	-2.575176	-1.539726
C	6.196176	-3.712918	-2.553951
C	7.319518	-3.732384	-3.587533
C	-1.229015	-3.455934	-1.159300
C	-0.736723	-2.410552	-0.167687
C	-1.675009	-1.200406	-0.197473
C	-3.046571	-1.427509	0.437130
C	-0.883786	-5.409493	0.407777
C	-2.257625	-5.190958	1.032063
C	-2.177430	-5.493719	2.540468
C	-1.496116	-4.380766	3.339540
O	1.421841	-3.687536	1.795910
C	2.267847	-4.583653	2.042878
O	1.662472	-5.855844	2.266490
C	2.592762	-6.913978	2.524019
O	3.518758	-4.530788	2.085205
H	-1.436625	1.668467	-4.350972
H	-0.470736	0.618263	-1.665120
H	-0.294350	3.431596	-2.866427
H	0.335127	2.930893	-1.275419
H	-1.933540	2.374928	-0.475891
H	-1.873853	4.059306	-1.024530
H	-2.914040	3.402593	-3.204252
H	-3.947447	2.915258	-1.849089
H	-3.097282	0.589287	-1.987929
H	-3.719647	1.087837	-3.585455
H	-3.157778	-1.116890	-3.533077

H	-1.585506	-6.076868	-6.172897
H	-3.711245	-3.357886	-3.620363
H	-0.107888	-4.379041	-8.083092
H	0.791553	-5.354809	-6.901119
H	1.076241	-3.588410	-7.027755
H	1.999205	2.297518	-2.318263
H	6.758105	-0.230526	-4.432188
H	4.196428	2.512726	-2.357026
H	5.883556	-1.763022	-6.025837
H	4.206999	-1.520919	-6.614771
H	4.506953	-2.576219	-5.232166
H	3.066660	-4.486423	-4.544031
H	1.343202	-4.256643	-4.085742
H	2.133177	-3.051314	-5.133788
H	7.726103	1.269343	-2.896052
H	6.990574	2.870163	-3.012444
H	7.583258	2.439069	-0.711053
H	5.827199	2.518603	-0.818405
H	7.341412	-0.145941	-0.883060
H	6.974049	0.688220	0.632633
H	4.549291	0.633657	0.150615
H	4.796519	-0.240585	-1.359942
H	3.538634	-2.541393	1.294808
H	3.031928	-1.346868	0.087558
H	4.242323	-2.429514	-1.230466
H	5.057986	-3.473280	-0.036555
H	7.109819	-1.403676	0.998375
H	5.955101	-0.696261	2.141916
H	-4.601694	-5.563727	-4.006809
H	-3.927216	-6.607769	-5.267184
H	-3.773218	-7.668806	-3.047817
H	-2.166839	-7.420247	-3.737488
H	-3.270613	-5.264125	-1.894125
H	-2.763772	-6.798746	-1.168515
H	-0.533369	-6.634957	-2.085449
H	-0.938421	-5.184229	-3.036592
H	-0.963726	-3.143182	-2.178149
H	-2.315608	-3.585593	-1.104934
H	-0.112880	-4.947060	1.040510
H	-0.657446	-6.482593	0.341684
H	0.997643	-4.173877	-2.122697
H	1.211437	-4.042223	-0.349324
H	3.197177	-6.705922	3.419379
H	1.989253	-7.818694	2.679268
H	3.278397	-7.060128	1.676640
H	5.079414	-3.196575	2.441876
H	6.564486	-3.649949	1.615827
H	7.042061	-3.739709	3.951126
H	7.891429	-2.311098	3.355105
H	6.015453	-0.839540	4.267446
H	5.169377	-2.303746	4.814893
H	6.731462	-1.843772	5.538153
H	6.422630	-1.633152	-2.106522
H	7.305651	-2.700836	-0.979831
H	5.224819	-3.578554	-3.056352
H	6.149515	-4.679380	-2.023981
H	7.346953	-2.783208	-4.143582
H	8.302462	-3.875003	-3.110309
H	7.177048	-4.543501	-4.316781
H	3.564878	-0.988715	3.057188
H	3.929982	0.400115	2.019628

H	1.272444	-1.112978	1.953131
H	1.552862	0.329195	2.944627
H	2.101246	1.661980	0.844711
H	1.696072	0.237240	-0.152290
H	0.446084	1.025283	0.834468
H	-0.653399	-2.809041	0.853289
H	0.269162	-2.079461	-0.462510
H	-1.174957	-0.367086	0.320532
H	-1.797655	-0.885817	-1.243919
H	-2.946952	-1.720600	1.493683
H	-3.654434	-0.510289	0.399567
H	-3.621129	-2.212934	-0.078340
H	-2.585840	-4.149346	0.910979
H	-3.023630	-5.826208	0.566626
H	-3.201991	-5.653210	2.916561
H	-1.636453	-6.444629	2.693531
H	-1.444552	-4.643427	4.407292
H	-0.469320	-4.191590	2.990326
H	-2.068207	-3.442483	3.253719
H	1.477594	-6.592823	-2.052762
H	1.438992	-6.590010	-0.266945
H	3.411199	-4.919464	-1.905660
H	3.430175	-5.190239	-0.158201
H	5.114708	-6.672301	-1.245172
H	3.818356	-7.670184	-0.555161
H	3.868009	-7.364709	-2.307801
C	-0.239187	0.596169	-7.032447
C	-1.545189	1.308444	-7.349735
O	-2.647985	0.989123	-6.428922
C	-3.302050	-0.176667	-6.535821
O	-4.254000	-0.443458	-5.824751
O	-2.790483	-0.984816	-7.492570
C	-3.475064	-2.257907	-7.610028
H	-1.870558	1.098472	-8.380766
H	0.474656	0.907852	-7.831873
H	-3.466611	-2.784065	-6.650101
H	-4.509643	-2.096643	-7.939284
H	-2.904847	-2.814561	-8.359531
H	-1.430594	2.392312	-7.208467
H	-0.376904	-0.489239	-7.151215

184

3t-P1C-EO ΔE=19.76 Fig. 8

C	0.438893	-0.841186	-0.016513
C	-0.005417	-2.071288	0.784106
C	1.171631	-2.999209	1.051633
C	2.258492	-2.228796	1.811999
C	2.686465	-0.966207	1.059441
C	1.490921	-0.053993	0.754361
N	-1.152550	-2.658658	0.078267
Co	-2.210840	-1.315218	-0.732778
O	-2.981731	-1.179978	1.095436
N	-0.787990	-0.121291	-0.377169
C	-0.854183	1.166294	-0.455714
C	-1.978505	1.928359	-0.900446
C	-1.928701	3.330703	-0.752439
C	-2.973373	4.149615	-1.138363
C	-4.094349	3.537791	-1.744340
C	-4.200788	2.170161	-1.910334
C	-3.147473	1.311477	-1.443376
C	-2.976468	5.628984	-0.856719
C	-4.038373	6.003842	0.191733

C	-3.813185	5.292982	1.529112
C	-4.924898	5.551206	2.552071
N	-5.807351	4.353485	2.882514
C	-6.448548	3.799453	1.623129
C	-7.410011	4.706032	0.879152
C	-7.888172	3.939872	-0.362724
C	-8.900033	4.731837	-1.186067
C	-5.411768	1.524216	-2.511833
O	-3.350242	0.036075	-1.522500
C	-1.505902	-3.889894	0.226768
C	-2.707614	-4.490416	-0.276651
C	-2.979808	-5.832046	0.079051
C	-4.129163	-6.485675	-0.327282
C	-4.968350	-5.818566	-1.249828
C	-4.766048	-4.502123	-1.614914
C	-3.681145	-3.760098	-1.031371
C	-4.600002	-7.741092	0.351915
C	-5.740418	-7.447464	1.361687
C	-5.412659	-6.305661	2.354432
C	-5.935296	-4.980782	1.806396
N	-5.577051	-3.692070	2.531603
C	-6.424780	-3.512524	3.774853
C	-6.139082	-2.241413	4.557947
C	-7.015800	-2.156040	5.808975
C	-6.731149	-0.864799	6.579721
C	-5.676829	-3.771016	-2.552127
O	-3.686666	-2.479232	-1.204040
O	-1.601775	-1.645790	-2.537586
C	-0.611956	-2.410217	-2.886469
O	-0.711981	-2.831275	-4.203848
C	-1.950398	-2.517254	-4.874512
O	0.376593	-2.785316	-2.255110
C	-4.087315	-3.644451	2.846004
C	-3.598081	-4.348400	4.106253
C	-2.071468	-4.506958	4.017685
C	-1.330663	-3.171171	3.934246
C	-5.810410	-2.534018	1.544885
C	-7.143755	-2.532026	0.818040
C	-7.212015	-1.308076	-0.109044
C	-8.410796	-1.358288	-1.052852
C	-4.971734	3.213191	3.463730
C	-4.306166	3.485448	4.812082
C	-5.064576	2.954521	6.035731
C	-4.173903	2.887746	7.273995
C	-6.846122	4.838691	3.883640
C	-7.848307	3.802910	4.392690
C	-9.187444	3.713587	3.654140
C	-10.154264	2.798201	4.406380
O	-6.884758	0.785800	3.718817
C	-7.874532	0.616262	2.957211
O	-8.706387	-0.451889	3.390047
C	-9.898238	-0.617366	2.611888
O	-8.205912	1.230632	1.917436
H	0.850857	-1.227234	-0.967416
H	-0.413114	-1.687848	1.735687
H	1.548641	-3.374381	0.084505
H	0.856417	-3.866776	1.652468
H	1.867153	-1.941750	2.805857
H	3.128931	-2.882731	1.985364
H	3.163657	-1.258221	0.107097
H	3.440811	-0.411990	1.642012

H	1.042772	0.329554	1.689028
H	1.837133	0.809556	0.164495
H	0.026024	1.744915	-0.157218
H	-4.917778	4.169090	-2.092074
H	-1.038421	3.770796	-0.293893
H	-5.120224	0.818990	-3.305185
H	-6.098200	2.275113	-2.927257
H	-5.951458	0.929132	-1.758261
H	-0.844172	-4.554784	0.792671
H	-5.842357	-6.344844	-1.646343
H	-2.277016	-6.342457	0.744052
H	-6.162437	-2.924761	-2.047935
H	-6.447876	-4.438267	-2.963123
H	-5.101051	-3.328393	-3.379586
H	-2.805259	-2.911047	-4.309617
H	-2.073296	-1.432316	-4.990569
H	-1.880274	-3.003466	-5.855912
H	-4.960106	-8.485740	-0.379332
H	-3.757573	-8.204645	0.893337
H	-6.660957	-7.199888	0.804130
H	-5.960153	-8.370058	1.924438
H	-5.857135	-6.524731	3.338524
H	-4.321850	-6.265040	2.477007
H	-5.556217	-4.839048	0.789013
H	-7.033115	-5.008304	1.756263
H	-3.831455	-2.579461	2.884874
H	-3.604784	-4.059399	1.951582
H	-4.977358	-2.585361	0.831194
H	-5.668382	-1.618280	2.128245
H	-7.471006	-3.519785	3.434952
H	-6.259967	-4.408902	4.391697
H	-1.979462	5.939067	-0.499142
H	-3.173184	6.201737	-1.780408
H	-4.051560	7.098163	0.344959
H	-5.029139	5.726259	-0.203526
H	-3.686104	4.216640	1.335182
H	-2.852366	5.620197	1.961145
H	-4.516457	5.888115	3.513284
H	-5.609977	6.334877	2.198629
H	-5.646437	2.341456	3.519679
H	-4.223350	2.976409	2.698316
H	-7.355398	5.687685	3.403319
H	-6.253959	5.242271	4.716453
H	-5.610292	3.536661	0.966639
H	-6.956458	2.859096	1.902738
H	-10.498000	0.308753	2.599663
H	-9.666661	-0.887278	1.568520
H	-10.465550	-1.428108	3.097407
H	-5.080277	-2.196045	4.849913
H	-6.338317	-1.343005	3.957880
H	-8.075093	-2.161739	5.499818
H	-6.853509	-3.038271	6.455999
H	-5.677795	-0.825618	6.903252
H	-6.922027	-0.006173	5.919587
H	-7.365590	-0.781772	7.475167
H	-7.252557	-3.440081	0.201043
H	-7.986122	-2.498874	1.528505
H	-6.279861	-1.259172	-0.696697
H	-7.265607	-0.389158	0.494325
H	-8.376490	-2.250095	-1.699363
H	-9.360070	-1.384577	-0.494327

H	-8.432101	-0.470162	-1.701600
H	-3.846848	-3.755376	5.001242
H	-4.053033	-5.337810	4.245905
H	-1.722081	-5.079097	4.893337
H	-1.832757	-5.118506	3.129011
H	-0.244009	-3.325068	3.857578
H	-1.652722	-2.578780	3.067754
H	-1.520291	-2.565341	4.835382
H	-3.327555	2.979253	4.772869
H	-4.054862	4.551948	4.950161
H	-5.944855	3.580015	6.251752
H	-5.459247	1.952324	5.802040
H	-3.767182	3.879044	7.531493
H	-4.731441	2.514219	8.145264
H	-3.320716	2.211223	7.105452
H	-7.395188	2.799569	4.422076
H	-8.054649	4.074040	5.443851
H	-9.628637	4.722472	3.549736
H	-9.035813	3.295468	2.649862
H	-9.723601	1.792126	4.522716
H	-10.373185	3.189108	5.413683
H	-11.107282	2.700094	3.866104
H	-6.938519	5.653043	0.567759
H	-8.283129	4.960798	1.498709
H	-7.012316	3.686267	-0.983616
H	-8.322505	2.980267	-0.035774
H	-8.472058	5.684197	-1.538340
H	-9.223638	4.161415	-2.068917
H	-9.796577	4.965534	-0.590177
C	-2.937504	-0.179835	1.930190
O	-1.630813	0.150619	2.445869
C	-1.864453	0.791662	3.695467
C	-3.119051	0.084479	4.257387
O	-3.688576	-0.585777	3.142095
O	-3.475471	1.061646	1.486217
C	-4.823820	0.875301	1.022651
H	-3.844133	0.789740	4.689253
H	-0.973446	0.655934	4.325186
H	-5.516070	0.769718	1.868559
H	-5.075158	1.759004	0.431868
H	-4.866298	0.004611	0.361767
H	-2.855042	-0.662724	5.024962
H	-2.043313	1.870090	3.542390

184

3c-P1-EO ΔE=14.37 Fig. 8

C	0.078586	-0.248346	0.951053
C	-0.962297	-1.340064	1.130047
C	-1.547474	-1.767852	-0.229858
C	-2.254434	-0.588904	-0.888407
C	-1.242423	0.551030	-1.062248
C	-0.588667	0.954446	0.265043
N	-0.572050	-2.608478	1.747940
C	0.660196	-3.000248	1.849092
C	1.054497	-4.360597	2.067090
C	2.403435	-4.669488	2.360239
C	2.832899	-5.980695	2.492428
C	1.857334	-7.000248	2.444013
C	0.536123	-6.760301	2.125267
C	0.131468	-5.426372	1.808189
C	4.282893	-6.376140	2.516534
C	4.905922	-6.475895	1.094708

C	3.963893	-7.003826	-0.017073
C	3.345531	-5.803635	-0.722333
N	2.333536	-6.071790	-1.815452
C	2.029471	-4.744652	-2.488802
C	1.468607	-3.668810	-1.557586
C	2.440511	-2.512928	-1.311647
C	2.651068	-1.660097	-2.565098
C	-0.492936	-7.846005	2.061689
O	-1.028616	-5.281953	1.237115
Co	-2.138898	-3.761929	1.692311
O	-3.207810	-2.325770	2.503914
N	-2.388802	-2.958886	0.016407
C	-3.151302	-3.439316	-0.913910
C	-3.867968	-4.667776	-0.846355
C	-4.397589	-5.157657	-2.057688
C	-4.927549	-6.432248	-2.149529
C	-4.979583	-7.204667	-0.970146
C	-4.540822	-6.740115	0.259206
C	-3.967106	-5.438005	0.352158
C	-5.333047	-7.018318	-3.475616
C	-4.135908	-7.402125	-4.366317
C	-3.311002	-6.184279	-4.809715
C	-2.531862	-6.519513	-6.067705
N	-1.879008	-5.318800	-6.747217
C	-2.851165	-4.157784	-6.874858
C	-4.182345	-4.483475	-7.539851
C	-5.169836	-3.320168	-7.350325
C	-5.641756	-3.155561	-5.903256
C	-4.604773	-7.566656	1.506924
O	-3.584528	-5.019082	1.529731
C	-0.691680	-4.873713	-5.901611
C	0.035047	-3.652244	-6.451261
C	1.316965	-3.369011	-5.656484
C	2.422159	-4.395896	-5.901171
C	-1.430755	-5.751192	-8.132763
C	-0.300886	-6.767054	-8.147643
C	-0.070880	-7.297899	-9.563983
C	1.083994	-8.299605	-9.600076
C	2.870885	-7.048206	-2.847627
C	4.204759	-6.680200	-3.479162
C	4.473847	-7.591045	-4.689232
C	4.571977	-9.075540	-4.330054
C	1.039052	-6.658498	-1.235445
C	-0.192985	-6.455117	-2.109011
C	-1.421567	-7.163856	-1.526503
C	-1.415068	-8.679105	-1.698112
O	-0.730101	-1.442519	-3.825453
C	-1.238428	-0.301268	-4.522234
C	-1.662023	-2.515547	-3.693598
O	-2.827652	-2.330876	-4.114559
O	-1.154313	-3.521931	-3.142611
O	0.997842	-7.378214	-5.196351
C	0.449478	-8.511433	-5.089487
O	1.187964	-9.384812	-4.247140
C	0.643341	-10.706557	-4.137954
O	-0.612893	-8.937533	-5.589540
H	-0.717574	-2.103902	-0.877598
H	-1.807404	-0.968456	1.734214
H	0.907783	-0.603153	0.316958
H	0.502566	0.043149	1.926963
H	-1.355064	1.374073	0.941162

H	0.158195	1.746603	0.095161
H	-0.463644	0.212532	-1.767731
H	-1.736085	1.423052	-1.521157
H	-3.097768	-0.280412	-0.245404
H	-2.664990	-0.877743	-1.867090
H	-3.212419	-2.900413	-1.864996
H	-5.376851	-8.222466	-1.028814
H	-4.309357	-4.517139	-2.936063
H	-3.605120	-7.646437	1.959942
H	-4.991237	-8.573536	1.298040
H	-5.243866	-7.086624	2.263833
H	1.459702	-2.260537	1.716842
H	2.168554	-8.029880	2.644557
H	3.121702	-3.848258	2.444035
H	-1.405582	-7.518119	2.580553
H	-0.118733	-8.769817	2.523762
H	-0.787905	-8.069497	1.026486
H	-5.954154	-7.913717	-3.306601
H	-5.963451	-6.297127	-4.026538
H	-3.490610	-8.129060	-3.846870
H	-4.529184	-7.918534	-5.261223
H	-4.005220	-5.353146	-4.996976
H	-2.639009	-5.847226	-4.007701
H	-1.738939	-7.260705	-5.868417
H	-3.203549	-6.953777	-6.823012
H	-0.043262	-5.759111	-5.807487
H	-1.092697	-4.641474	-4.905202
H	-1.148703	-4.834922	-8.671501
H	-2.326037	-6.162836	-8.620876
H	-2.328677	-3.389753	-7.460714
H	-2.982986	-3.752638	-5.861066
H	-0.426789	0.438945	-4.529831
H	-1.520236	-0.557140	-5.556112
H	-2.125751	0.116216	-4.023682
H	4.882113	-5.671806	3.116290
H	4.368708	-7.359638	3.007578
H	5.265296	-5.475098	0.796802
H	5.803030	-7.112970	1.151914
H	4.540559	-7.608058	-0.733151
H	3.184546	-7.652008	0.410725
H	2.844338	-5.160157	0.008870
H	4.145028	-5.217366	-1.199038
H	2.931272	-8.016638	-2.335394
H	2.100445	-7.137092	-3.634213
H	0.893730	-6.178331	-0.263586
H	1.251406	-7.721891	-1.062932
H	2.973884	-4.418225	-2.937834
H	1.339342	-4.982514	-3.303114
H	0.528900	-11.170397	-5.128015
H	1.359644	-11.278916	-3.532905
H	-0.338023	-10.697529	-3.642740
H	0.628632	-6.310822	-7.774275
H	-0.517787	-7.609551	-7.470640
H	-0.993125	-7.784154	-9.927964
H	0.137083	-6.461161	-10.255336
H	1.244512	-8.693563	-10.614208
H	2.020123	-7.827706	-9.264231
H	0.879719	-9.145656	-8.927096
H	0.301833	-3.789234	-7.514279
H	-0.610327	-2.763430	-6.382195
H	1.674442	-2.362254	-5.931001

H	1.046731	-3.307166	-4.591936
H	3.336121	-4.130044	-5.347430
H	2.123160	-5.410668	-5.602402
H	2.692871	-4.431439	-6.969984
H	-4.038481	-4.673647	-8.616091
H	-4.630521	-5.392151	-7.107484
H	-6.037954	-3.496784	-8.007480
H	-4.702547	-2.382179	-7.699779
H	-6.389539	-2.351115	-5.828211
H	-6.116294	-4.082514	-5.543028
H	-4.815727	-2.900117	-5.223390
H	4.191629	-5.639702	-3.837771
H	5.036695	-6.767468	-2.756999
H	3.655725	-7.451566	-5.415083
H	5.407893	-7.254250	-5.170149
H	3.591494	-9.464527	-4.021501
H	4.895252	-9.665213	-5.201334
H	5.302288	-9.244660	-3.520537
H	-0.435351	-5.386632	-2.169673
H	-0.004736	-6.814768	-3.133437
H	-2.305853	-6.753842	-2.034677
H	-1.543055	-6.872837	-0.472114
H	-0.558989	-9.160516	-1.196455
H	-2.334363	-9.119689	-1.280952
H	-1.358832	-8.943779	-2.765677
H	0.552921	-3.289023	-2.041757
H	1.135645	-4.106464	-0.605203
H	2.027893	-1.875453	-0.515226
H	3.409051	-2.885162	-0.929302
H	3.298341	-0.793394	-2.360866
H	3.122283	-2.239176	-3.374927
H	1.681514	-1.303624	-2.946372
C	-4.584290	-2.537088	2.665882
C	-4.876543	-2.974756	4.104901
O	-3.984535	-4.097673	4.398975
C	-2.683289	-3.776146	4.426589
O	-1.819644	-4.470982	3.884228
O	-2.415063	-2.739196	5.242013
C	-1.084317	-2.190709	5.075409
H	-5.141287	-1.597168	2.466628
H	-5.890094	-3.376710	4.232347
H	-0.353049	-2.998350	4.950439
H	-1.093857	-1.549173	4.184340
H	-0.891929	-1.606318	5.983238
H	-4.965070	-3.309676	1.976481
H	-4.691174	-2.167311	4.826794

184

3c-P1C-EO ΔE=27.48 Fig. 8

C	-3.971931	-6.193496	-5.081026
C	-3.594522	-6.702300	-3.697473
C	-2.104405	-6.432622	-3.452617
C	-1.258851	-7.266713	-4.401556
C	-1.618463	-6.858208	-5.837037
C	-3.127561	-6.967545	-6.111865
N	-4.209284	-6.130067	-2.494124
C	-4.577824	-4.884688	-2.510858
C	-4.688382	-4.027444	-1.380367
C	-5.213146	-2.731543	-1.564916
C	-5.294220	-1.822727	-0.528140
C	-4.955510	-2.274893	0.767842
C	-4.413263	-3.525516	1.002329

C	-4.139073	-4.391139	-0.108989
C	-5.562435	-0.371322	-0.810236
C	-4.369516	0.288764	-1.529649
C	-3.037065	0.240443	-0.758240
C	-2.289309	-1.091330	-0.875404
N	-0.804017	-1.057493	-0.490242
C	0.074004	-0.652160	-1.672908
C	-0.099518	0.766350	-2.204529
C	-1.019345	0.882494	-3.422384
C	-1.202136	2.328721	-3.875439
C	-4.099948	-4.036156	2.375302
O	-3.396190	-5.428367	0.108956
Co	-3.344828	-7.011523	-0.982963
O	-5.185524	-7.533246	0.086973
N	-1.847020	-6.651577	-2.017224
C	-0.694920	-6.356372	-1.510527
C	-0.338533	-6.517633	-0.146170
C	0.907490	-5.987352	0.254206
C	1.420374	-6.204138	1.515265
C	0.635215	-6.982347	2.399317
C	-0.611950	-7.488033	2.072035
C	-1.155555	-7.251290	0.768821
C	2.745706	-5.637521	1.976599
C	3.390929	-4.636408	1.016360
C	3.959294	-5.264711	-0.277712
C	3.871704	-4.215451	-1.373728
N	4.338643	-4.641736	-2.764676
C	5.680784	-5.330197	-2.701292
C	6.802944	-4.479769	-2.116836
C	8.017546	-5.342803	-1.745733
C	7.787777	-6.237092	-0.525808
C	-1.454700	-8.249657	3.048565
O	-2.335347	-7.732186	0.495028
C	3.297058	-5.587774	-3.338245
C	3.495869	-5.992279	-4.790487
C	2.221571	-6.684921	-5.286276
C	2.336395	-7.167986	-6.729288
C	4.470330	-3.380523	-3.615432
C	3.156474	-2.689713	-3.921999
C	3.404763	-1.434698	-4.760414
C	2.123466	-0.611249	-4.883626
C	-0.586809	-0.154989	0.706514
C	0.862358	0.118918	1.072045
C	0.946894	1.064417	2.278005
C	0.448980	0.473025	3.598681
C	-0.377835	-2.506639	-0.186541
C	-0.961120	-3.121545	1.082803
C	-0.011515	-3.112568	2.282605
C	-0.571458	-3.864295	3.486592
O	-1.290679	-3.104621	-3.460961
C	-2.060393	-2.642809	-4.572111
C	-0.045041	-3.754340	-3.817197
O	0.194191	-3.912817	-5.027834
O	0.603518	-4.072016	-2.788534
O	4.880011	-1.203402	-1.356698
C	3.689149	-0.938970	-1.078230
O	3.186689	0.349265	-1.476184
C	4.158492	1.208469	-2.078531
O	2.805468	-1.626270	-0.510951
H	-1.931620	-5.357260	-3.638672
H	-3.757870	-7.787808	-3.630853

H	-3.754348	-5.116123	-5.178302
H	-5.048879	-6.339673	-5.268401
H	-3.426385	-8.031328	-6.084575
H	-3.350098	-6.599226	-7.126577
H	-1.279707	-5.818250	-5.990705
H	-1.066340	-7.483944	-6.556812
H	-1.467237	-8.335740	-4.218897
H	-0.191940	-7.089764	-4.212641
H	0.052244	-5.912904	-2.179165
H	1.012686	-7.161765	3.410887
H	1.425632	-5.370652	-0.477205
H	-0.978102	-8.291215	4.037539
H	-1.632114	-9.277495	2.694005
H	-2.447625	-7.781744	3.139077
H	-4.776758	-4.424918	-3.486580
H	-5.104596	-1.599522	1.615937
H	-5.523141	-2.437955	-2.572318
H	-4.190789	-3.242174	3.129432
H	-3.094379	-4.471267	2.417148
H	-4.796299	-4.851433	2.633934
H	2.593813	-5.140607	2.951978
H	3.447831	-6.470582	2.174852
H	2.664141	-3.854249	0.746752
H	4.201505	-4.097718	1.532874
H	4.998641	-5.576959	-0.094457
H	3.390127	-6.171613	-0.534994
H	2.833747	-3.892743	-1.512790
H	4.436327	-3.308893	-1.113173
H	2.328101	-5.073157	-3.215481
H	3.302076	-6.473341	-2.686042
H	4.993134	-3.683095	-4.535437
H	5.103735	-2.695836	-3.034214
H	5.925658	-5.633867	-3.729107
H	5.521639	-6.242303	-2.110755
H	-2.236346	-3.446632	-5.301570
H	-3.018094	-2.295586	-4.158041
H	-1.559409	-1.812967	-5.095173
H	-6.463516	-0.252112	-1.436877
H	-5.758606	0.166196	0.133472
H	-4.236675	-0.184810	-2.517998
H	-4.615229	1.347746	-1.721426
H	-2.409954	1.056115	-1.140377
H	-3.238226	0.464892	0.303309
H	-2.751940	-1.844508	-0.231461
H	-2.301483	-1.488922	-1.898550
H	-1.077957	0.798312	0.479743
H	-1.145735	-0.620484	1.529779
H	0.719467	-2.479668	-0.159456
H	-0.661340	-3.067545	-1.086423
H	1.103008	-0.828433	-1.328401
H	-0.155206	-1.397760	-2.446242
H	3.647738	2.161581	-2.273779
H	5.016861	1.371252	-1.409843
H	4.535966	0.792674	-3.024090
H	2.454662	-3.342935	-4.460632
H	2.662943	-2.401089	-2.983066
H	4.194569	-0.827124	-4.288558
H	3.771853	-1.721693	-5.762848
H	1.303338	-1.232955	-5.276218
H	1.834691	-0.241132	-3.890952
H	2.255621	0.256969	-5.547214

H	3.665689	-5.104538	-5.420284
H	4.364530	-6.661927	-4.915789
H	1.989833	-7.538720	-4.624609
H	1.395046	-5.959451	-5.202192
H	1.407494	-7.659064	-7.053880
H	2.519783	-6.321563	-7.409440
H	3.160417	-7.889436	-6.851433
H	7.107376	-3.714167	-2.846492
H	6.455748	-3.935266	-1.225147
H	8.864823	-4.667477	-1.542395
H	8.316938	-5.958150	-2.613222
H	8.698250	-6.801364	-0.273677
H	7.512925	-5.632603	0.352627
H	6.986493	-6.972946	-0.691469
H	1.416170	-0.809034	1.269691
H	1.385146	0.584897	0.224022
H	2.002509	1.361112	2.392221
H	0.390603	1.994212	2.055162
H	0.565592	1.195418	4.421022
H	-0.615747	0.196187	3.557515
H	1.019839	-0.430062	3.863550
H	-1.918067	-2.646701	1.354247
H	-1.226615	-4.163901	0.841279
H	0.229192	-2.080445	2.574917
H	0.937996	-3.573022	1.972366
H	-0.816051	-4.902322	3.216462
H	0.158232	-3.893701	4.311110
H	-1.487633	-3.385507	3.866583
H	-0.419824	1.460316	-1.409287
H	0.909976	1.114687	-2.480738
H	-0.584934	0.285071	-4.240937
H	-2.003541	0.434513	-3.212052
H	-1.833533	2.395027	-4.773896
H	-1.676250	2.933446	-3.085230
H	-0.230209	2.791628	-4.108860
C	-5.614135	-8.498631	-0.522722
O	-3.538804	-8.737188	-1.817944
C	-2.600101	-9.714269	-1.444397
O	-5.612226	-9.755376	-0.022559
C	-6.270289	-10.630733	-0.977139
C	-6.337166	-9.773018	-2.238285
O	-6.264559	-8.424616	-1.708299
H	-5.655032	-11.530830	-1.088622
H	-7.274095	-9.869555	-2.798374
H	-2.772228	-10.610649	-2.074962
H	-1.555355	-9.389040	-1.611877
H	-2.687950	-10.013562	-0.384009
H	-7.260507	-10.886145	-0.571573
H	-5.457310	-9.912793	-2.881066

184

3t-P1-EO ΔE=0.00 Fig. 9

C	-2.391081	-1.338526	4.547382
C	-3.111088	-0.022998	4.864397
C	-2.986138	0.342858	6.337561
C	-1.495912	0.343242	6.791830
C	-0.529458	0.008205	5.650660
C	-0.902356	-1.306329	4.960458
N	-4.462191	-0.191165	4.336496
Co	-4.420140	-1.024120	2.632928
O	-3.821490	0.695630	1.926667
N	-2.672991	-1.598027	3.126041

C	-1.923921	-2.393520	2.440969
C	-2.166144	-2.797404	1.092597
C	-1.175934	-3.589847	0.491108
C	-1.240306	-3.964254	-0.840572
C	-2.344860	-3.510925	-1.585978
C	-3.368952	-2.753003	-1.027737
C	-3.332505	-2.414684	0.361420
C	-0.059021	-4.663649	-1.468030
C	1.182104	-3.754569	-1.385808
C	0.964382	-2.457759	-2.181812
C	1.708814	-1.246682	-1.614112
N	0.798990	-0.091488	-1.204727
C	1.680265	1.026864	-0.680854
C	2.638132	1.689259	-1.660229
C	3.646386	2.542824	-0.869978
C	4.660434	1.705416	-0.085647
C	-4.558108	-2.310058	-1.826754
O	-4.367556	-1.803864	0.865226
C	-5.535556	0.092275	4.993855
C	-6.869180	-0.076659	4.502524
C	-7.939651	0.298601	5.345977
C	-9.260265	0.124054	4.976541
C	-9.511730	-0.589420	3.780006
C	-8.503334	-0.958816	2.909212
C	-7.147769	-0.565689	3.184632
C	-10.377287	0.793180	5.724817
C	-10.918668	2.032420	4.970366
C	-9.813024	2.998888	4.482962
C	-9.430651	2.672116	3.045522
N	-8.270959	3.461205	2.467958
C	-8.500166	4.959061	2.550210
C	-9.796127	5.472791	1.939299
C	-9.873649	7.001007	2.084635
C	-8.839320	7.750148	1.242244
C	-8.741587	-1.809919	1.703195
O	-6.282992	-0.656640	2.225645
O	-5.170862	-2.619224	3.545152
C	-5.389350	-3.662577	2.798498
O	-4.206042	-4.278387	2.427926
C	-4.348526	-5.279520	1.408389
O	-6.479697	-4.131204	2.472595
C	-6.955076	3.077575	3.186480
C	-6.477259	4.033787	4.271010
C	-5.227907	3.481465	4.973788
C	-4.033765	3.252325	4.049086
C	-8.130081	3.105892	0.987852
C	-7.892414	1.641940	0.671348
C	-7.526121	1.506208	-0.813343
C	-7.353756	0.047748	-1.238989
C	-0.126317	0.371948	-2.317189
C	0.411560	0.375428	-3.742430
C	-0.761220	0.432741	-4.737290
C	-1.597958	-0.849867	-4.768547
C	-0.095478	-0.593355	-0.049531
C	-0.825345	0.497312	0.722045
C	-0.127727	1.016843	1.985028
C	-0.861734	2.229797	2.550906
O	2.835058	-0.845045	1.425641
C	2.102516	-1.364367	2.312586
O	2.215682	-0.857128	3.633010
C	3.143287	0.220848	3.779399

O	1.232586	-2.260311	2.205730
H	-2.933643	-2.131719	5.095195
H	-2.649520	0.755824	4.236391
H	-3.563093	-0.387188	6.930846
H	-3.439260	1.331414	6.512811
H	-1.236334	1.320208	7.229559
H	-1.364849	-0.402702	7.593376
H	0.502159	-0.067757	6.027575
H	-0.524150	0.825440	4.912502
H	-0.234325	-1.459670	4.100121
H	-0.703873	-2.145686	5.647214
H	-0.991136	-2.759510	2.882488
H	-2.408283	-3.767001	-2.648870
H	-0.298806	-3.838203	1.092096
H	-5.489581	-2.711007	-1.395042
H	-4.478557	-2.636419	-2.873595
H	-4.662922	-1.214346	-1.800203
H	-5.441691	0.479323	6.014889
H	-10.544118	-0.855288	3.529903
H	-7.703547	0.763891	6.307933
H	-9.804730	-2.071484	1.604387
H	-8.137055	-2.729526	1.808262
H	-8.399112	-1.319045	0.785010
H	-4.854799	-6.172970	1.801538
H	-4.927822	-4.884693	0.562405
H	-3.328127	-5.517511	1.083675
H	-11.215226	0.097436	5.904321
H	-10.010876	1.113967	6.715128
H	-11.513187	1.694242	4.103447
H	-11.616789	2.574732	5.629153
H	-10.168674	4.039051	4.559240
H	-8.934316	2.897507	5.138011
H	-9.143086	1.618093	2.961858
H	-10.286529	2.839749	2.374219
H	-6.199884	2.996246	2.400668
H	-7.119769	2.073755	3.595172
H	-7.292771	3.722831	0.633707
H	-9.053415	3.449090	0.503261
H	-8.479189	5.224924	3.613003
H	-7.620427	5.401178	2.066288
H	0.150623	-5.614754	-0.949324
H	-0.280666	-4.915470	-2.520070
H	1.365446	-3.504016	-0.327811
H	2.081235	-4.281889	-1.747493
H	1.250201	-2.614059	-3.235064
H	-0.116150	-2.253661	-2.199488
H	2.256468	-1.482945	-0.685001
H	2.421708	-0.835521	-2.336689
H	-0.997362	-0.291751	-2.261340
H	-0.468243	1.376874	-2.031275
H	0.540137	-1.200681	0.612935
H	-0.813837	-1.265547	-0.529184
H	0.997926	1.786871	-0.281237
H	2.220546	0.557625	0.157204
H	3.168750	0.458254	4.851544
H	4.147279	-0.061146	3.429911
H	2.818953	1.109427	3.214660
H	-9.866211	5.221644	0.869584
H	-10.665402	5.020020	2.442668
H	-10.886809	7.321422	1.789963
H	-9.762135	7.272967	3.149391

H	-8.971490	8.837816	1.338456
H	-7.807472	7.514471	1.541131
H	-8.944821	7.491906	0.176708
H	-7.076991	1.217796	1.274712
H	-8.790955	1.041984	0.885422
H	-6.588973	2.062239	-0.988655
H	-8.298293	1.991563	-1.438901
H	-8.315649	-0.486548	-1.213675
H	-6.960359	-0.021762	-2.264360
H	-6.662696	-0.481032	-0.566620
H	-6.223523	5.009689	3.828885
H	-7.257119	4.196737	5.031527
H	-4.956889	4.194894	5.771233
H	-5.490708	2.539344	5.476979
H	-3.788987	4.171839	3.492353
H	-3.141402	2.985853	4.634155
H	-4.204089	2.435679	3.327802
H	1.088423	1.225482	-3.909184
H	0.984892	-0.541400	-3.943695
H	-1.406258	1.298095	-4.500256
H	-0.349911	0.623415	-5.742160
H	-0.968528	-1.720291	-5.011333
H	-2.092013	-1.057329	-3.807839
H	-2.388817	-0.781867	-5.530416
H	-1.088423	1.337066	0.054098
H	-1.792006	0.084248	1.047743
H	-0.115504	0.201157	2.722779
H	0.929104	1.259407	1.803473
H	-1.923588	1.985535	2.697101
H	-0.430200	2.557916	3.509028
H	-0.812304	3.086961	1.855629
H	2.090392	2.328748	-2.370978
H	3.200514	0.948923	-2.250118
H	4.174848	3.202902	-1.578328
H	3.094205	3.208450	-0.181391
H	5.352958	2.354764	0.470792
H	4.179516	1.025210	0.633263
H	5.261051	1.087434	-0.772971
C	-4.126864	0.911863	0.590315
C	-3.787337	2.331667	0.186052
O	-4.840691	3.248667	0.677703
C	-4.565792	4.527048	0.932424
O	-5.429248	5.307280	1.307852
O	-3.268046	4.851555	0.740087
C	-2.954294	6.222637	1.089288
H	-2.831770	2.639737	0.618153
H	-3.541511	0.274228	-0.112633
H	-3.554785	6.914192	0.486193
H	-3.153609	6.393043	2.153921
H	-1.888159	6.333690	0.871946
H	-3.770023	2.444492	-0.910131
H	-5.185062	0.707839	0.346157

184

TS ΔE=21.35 Fig. 9

C	-1.481053	3.246355	1.407275
C	-0.613485	2.434240	2.366433
C	0.564257	3.266848	2.894094
C	0.182339	4.758771	3.025773
C	-1.313461	4.917829	3.304256
C	-2.150527	4.441124	2.102714
N	-0.229070	1.200274	1.651251

Co	-1.430004	0.732301	0.259015
O	-3.586417	-1.003479	3.006187
N	-2.376732	2.273029	0.759192
C	-3.596044	2.569372	0.442401
C	-4.476756	1.707723	-0.280025
C	-5.792214	2.160713	-0.489423
C	-6.733648	1.391163	-1.147929
C	-6.325572	0.144354	-1.660081
C	-5.038445	-0.340714	-1.498471
C	-4.081069	0.423168	-0.763864
C	-8.176143	1.812705	-1.182822
C	-8.974925	1.081803	-0.080291
C	-8.418767	1.303813	1.335218
C	-8.609809	2.755170	1.745826
N	-8.340782	3.098655	3.206513
C	-8.384325	4.624611	3.330807
C	-9.596780	5.315121	2.739491
C	-9.543599	6.815424	3.072323
C	-8.309207	7.514405	2.494991
C	-4.620692	-1.669408	-2.046645
O	-2.906290	-0.113644	-0.565200
C	0.797252	0.503559	2.032120
C	1.240716	-0.719267	1.440883
C	2.268998	-1.435446	2.097204
C	2.709527	-2.665656	1.651977
C	2.197094	-3.134573	0.417925
C	1.199387	-2.475376	-0.274137
C	0.612401	-1.286747	0.284187
C	3.557399	-3.551808	2.522101
C	2.746108	-4.689873	3.190480
C	1.471323	-4.221452	3.929815
C	0.283618	-4.254078	2.972864
N	-1.029576	-3.656815	3.442261
C	-1.455385	-4.302365	4.742990
C	-2.853305	-3.899593	5.205521
C	-3.023797	-4.105288	6.713121
C	-2.917561	-5.571563	7.137719
C	0.645250	-2.978390	-1.571806
O	-0.475145	-0.850802	-0.264621
O	-0.367727	1.784638	-0.956386
C	-0.935963	2.436364	-1.940037
O	-2.195253	1.995565	-2.228944
C	-2.929079	2.813937	-3.155851
O	-0.382712	3.352672	-2.543305
C	-0.975059	-2.131160	3.529675
C	-0.235209	-1.521180	4.717049
C	-0.854624	-0.174150	5.120811
C	-2.200388	-0.325823	5.829637
C	-2.100452	-4.006525	2.405032
C	-1.859702	-3.460730	1.011033
C	-2.816019	-4.116769	0.008635
C	-2.497148	-5.584714	-0.276897
C	-9.380751	2.429869	4.076497
C	-9.293071	2.757234	5.568201
C	-10.257850	3.850409	6.040601
C	-10.085687	4.163815	7.525353
C	-6.911046	2.748712	3.621126
C	-6.557011	1.312764	3.964406
C	-5.215428	1.312732	4.719424
C	-4.058950	1.906694	3.912970
O	-7.043796	5.111073	0.383769

C	-5.906512	5.200663	0.913683
O	-4.816285	5.558799	0.088347
C	-5.143377	5.731595	-1.298785
O	-5.554087	4.973807	2.101221
H	-0.813754	3.600192	0.599971
H	-1.267307	2.119958	3.199507
H	1.414523	3.154301	2.201514
H	0.878407	2.874108	3.874747
H	0.787865	5.226912	3.817365
H	0.426744	5.285948	2.087517
H	-1.564170	5.966243	3.528328
H	-1.579907	4.332745	4.202804
H	-3.165519	4.193916	2.439591
H	-2.275619	5.256337	1.373796
H	-3.992336	3.547008	0.732709
H	-7.048587	-0.465930	-2.210612
H	-6.085898	3.138235	-0.111014
H	-3.608494	-1.605937	-2.471786
H	-5.323896	-2.020692	-2.815093
H	-4.579925	-2.420091	-1.243155
H	1.369427	0.851140	2.900347
H	2.580375	-4.075528	0.011040
H	2.684667	-1.014220	3.016797
H	1.106574	-3.932760	-1.859189
H	0.820730	-2.239732	-2.370373
H	-0.445856	-3.101191	-1.511401
H	-3.910545	2.332797	-3.241336
H	-3.041709	3.831673	-2.759414
H	-2.421298	2.854984	-4.128676
H	4.380687	-4.004743	1.942943
H	4.024443	-2.940462	3.312673
H	2.469411	-5.436174	2.424393
H	3.406473	-5.213133	3.901266
H	1.285864	-4.880378	4.792661
H	1.632386	-3.207463	4.318116
H	0.537896	-3.717648	2.056061
H	0.057872	-5.298390	2.700637
H	-2.028620	-1.784296	3.447944
H	-0.492773	-1.823086	2.596313
H	-3.038345	-3.592074	2.787608
H	-2.160043	-5.105065	2.415475
H	-1.382163	-5.386958	4.569352
H	-0.701968	-4.035346	5.494452
H	-8.243252	2.904816	-1.050493
H	-8.628839	1.576355	-2.160779
H	-10.035822	1.389105	-0.121200
H	-8.947937	-0.001021	-0.289147
H	-8.934338	0.615960	2.024036
H	-7.352637	1.036754	1.336343
H	-7.974372	3.451655	1.170769
H	-9.657538	3.043600	1.574970
H	-10.356699	2.729685	3.667635
H	-9.267491	1.354519	3.902251
H	-6.713145	3.383307	4.498077
H	-6.290647	3.170021	2.817624
H	-8.292890	4.835039	4.405917
H	-7.456476	4.957000	2.834137
H	-4.217872	6.066225	-1.783882
H	-5.477859	4.784727	-1.748254
H	-5.936498	6.480832	-1.428775
H	-3.049763	-2.848649	4.944880

H	-3.606741	-4.501185	4.674896
H	-2.277758	-3.498720	7.257549
H	-4.011872	-3.710014	7.002430
H	-3.115460	-5.693047	8.213339
H	-3.645948	-6.185619	6.584934
H	-1.914858	-5.982734	6.941475
H	-2.017788	-2.373779	1.001589
H	-0.831843	-3.648879	0.671841
H	-2.752022	-3.540693	-0.925799
H	-3.852522	-4.016463	0.372264
H	-3.148330	-5.982949	-1.069871
H	-1.453139	-5.698306	-0.611279
H	-2.642286	-6.222190	0.608791
H	-0.255841	-2.176120	5.602066
H	0.825959	-1.372436	4.463896
H	-0.135152	0.357480	5.767700
H	-0.993904	0.439048	4.217378
H	-2.964102	-0.678201	5.119448
H	-2.123642	-1.032766	6.672637
H	-2.544093	0.637812	6.234371
H	-9.509752	1.832054	6.129945
H	-8.258992	3.024008	5.840755
H	-10.111670	4.770297	5.453426
H	-11.293713	3.521297	5.843316
H	-10.800050	4.931467	7.857125
H	-10.243564	3.265139	8.142160
H	-9.070451	4.537035	7.731738
H	-7.321188	0.841752	4.604538
H	-6.456586	0.702327	3.058575
H	-5.350847	1.859977	5.671801
H	-4.963080	0.270281	4.966381
H	-4.307565	2.886402	3.483950
H	-3.179819	2.047850	4.559432
H	-3.767835	1.195142	3.123838
H	-10.542111	4.890723	3.121186
H	-9.583891	5.208445	1.644121
H	-10.458461	7.283393	2.670994
H	-9.581206	6.952837	4.168871
H	-8.377233	8.603314	2.636627
H	-7.378808	7.170618	2.970126
H	-8.210937	7.300713	1.420199
C	-4.527792	-1.144761	2.037493
C	-5.721363	-2.001966	2.446896
O	-5.218102	-3.374562	2.660111
C	-5.955681	-4.278408	3.300486
O	-5.527893	-5.383027	3.589329
O	-7.210886	-3.834915	3.585828
C	-8.014735	-4.817886	4.280741
H	-6.145541	-1.649861	3.397475
H	-4.999894	-0.175911	1.700966
H	-8.983306	-4.334223	4.441448
H	-8.124917	-5.719937	3.666463
H	-7.547134	-5.085131	5.236210
H	-6.499711	-2.043111	1.666113
H	-4.138846	-1.576272	1.079965

184

3t-P1C-EO ΔE=18.37 Fig. 9

C	-2.179861	-2.000393	4.685023
C	-2.849549	-0.681625	5.080463
C	-2.744329	-0.421734	6.588644
C	-1.408314	-0.947018	7.165748

C	-0.318403	-0.992287	6.093757
C	-0.672950	-2.012193	4.999461
N	-4.225339	-0.735352	4.546438
Co	-4.364559	-1.795956	2.983791
O	-4.330985	2.013715	0.832140
N	-2.556640	-2.227362	3.276730
C	-1.749550	-2.787265	2.430864
C	-2.069330	-3.059592	1.063757
C	-1.039633	-3.579987	0.261522
C	-1.187488	-3.718080	-1.109622
C	-2.434755	-3.393357	-1.675564
C	-3.494457	-2.905078	-0.923020
C	-3.319705	-2.696348	0.476983
C	0.029403	-3.966112	-1.965095
C	1.007539	-2.782882	-1.794465
C	0.348399	-1.440259	-2.147129
C	1.025157	-0.256313	-1.455069
N	0.055418	0.676570	-0.734003
C	0.880029	1.774150	-0.083525
C	1.646908	2.741717	-0.976571
C	2.747695	3.418387	-0.140850
C	3.913579	2.485171	0.195302
C	-4.814594	-2.558659	-1.540243
O	-4.308791	-2.149607	1.135652
C	-5.200977	-0.090831	5.108136
C	-6.558559	-0.073679	4.663066
C	-7.502456	0.675716	5.407432
C	-8.837477	0.738041	5.056171
C	-9.263147	-0.072696	3.977053
C	-8.388238	-0.816795	3.210924
C	-6.979039	-0.756668	3.479023
C	-9.808310	1.649769	5.762160
C	-10.381141	2.774593	4.868597
C	-9.291015	3.588628	4.135119
C	-9.146044	3.084352	2.704100
N	-7.895189	3.489040	1.960948
C	-7.782739	4.988541	1.725753
C	-8.926722	5.643992	0.967673
C	-8.613632	7.136573	0.763867
C	-7.448692	7.386695	-0.196560
C	-8.846994	-1.729385	2.118076
O	-6.183349	-1.314744	2.618973
O	-4.944795	-3.223022	4.111904
C	-4.758569	-4.466671	3.736176
O	-4.244488	-4.583156	2.481130
C	-3.920891	-5.925246	2.072794
O	-5.022724	-5.420773	4.460454
C	-6.653177	2.991179	2.724086
C	-6.007897	4.020179	3.649966
C	-5.074650	3.345417	4.656942
C	-3.909258	2.611872	4.006634
C	-7.923438	2.842595	0.579224
C	-7.931259	1.326665	0.519300
C	-8.049688	0.943712	-0.964111
C	-7.914238	-0.555215	-1.218138
C	-1.042949	1.200946	-1.638577
C	-0.634648	1.794624	-2.979760
C	-1.865479	1.910678	-3.895192
C	-2.403920	0.560601	-4.374864
C	-0.635377	-0.146054	0.383232
C	-1.212869	0.663310	1.543319

C	-0.212216	0.871714	2.695203
C	-0.448964	2.122123	3.531374
O	2.575657	-0.482939	1.351620
C	2.015239	-1.317077	2.108978
O	2.254616	-1.210109	3.500653
C	3.114707	-0.128999	3.876264
O	1.211821	-2.240360	1.824031
H	-2.699048	-2.797002	5.250173
H	-2.335641	0.121622	4.522869
H	-3.589807	-0.916276	7.094973
H	-2.833700	0.660457	6.776349
H	-1.102441	-0.320275	8.017564
H	-1.556790	-1.966302	7.561545
H	0.656421	-1.257462	6.529716
H	-0.195848	0.009874	5.648567
H	-0.063144	-1.803558	4.108902
H	-0.388770	-3.024805	5.329175
H	-0.716166	-2.999512	2.726879
H	-2.569112	-3.505877	-2.755487
H	-0.072845	-3.761190	0.732234
H	-5.638867	-2.965864	-0.936968
H	-4.887177	-2.945239	-2.565955
H	-4.962004	-1.469969	-1.571779
H	-4.981858	0.503025	6.001885
H	-10.330767	-0.117152	3.739476
H	-7.150792	1.229814	6.281222
H	-9.905416	-1.564664	1.872461
H	-8.716822	-2.778136	2.433454
H	-8.232533	-1.602120	1.220128
H	-3.217829	-6.383356	2.780689
H	-4.828250	-6.540153	2.012163
H	-3.456657	-5.812935	1.086435
H	-10.650346	1.066076	6.174522
H	-9.296828	2.113249	6.622814
H	-11.080283	2.341115	4.131869
H	-10.987094	3.442874	5.501801
H	-9.533604	4.663366	4.143105
H	-8.344336	3.461183	4.677533
H	-9.122481	1.989263	2.715738
H	-10.003238	3.399106	2.090287
H	-5.922333	2.691803	1.959976
H	-6.984383	2.103275	3.277865
H	-7.032162	3.239111	0.066068
H	-8.830720	3.225306	0.093248
H	-7.697621	5.461305	2.710220
H	-6.825539	5.087700	1.192726
H	0.536859	-4.899153	-1.669247
H	-0.267590	-4.076818	-3.021618
H	1.326221	-2.747625	-0.738671
H	1.915191	-2.944310	-2.400670
H	0.322215	-1.292464	-3.239414
H	-0.702504	-1.495363	-1.834946
H	1.716153	-0.581591	-0.657710
H	1.584647	0.362650	-2.164423
H	-1.684810	0.330608	-1.818098
H	-1.635883	1.925365	-1.057107
H	0.106069	-0.875484	0.746370
H	-1.428577	-0.701393	-0.127986
H	0.187053	2.345826	0.543526
H	1.578124	1.218866	0.557377
H	3.247318	-0.210880	4.963151

H	4.087236	-0.195580	3.367826
H	2.665088	0.845828	3.631611
H	-9.071635	5.186113	-0.022471
H	-9.873563	5.545876	1.524987
H	-9.521811	7.629169	0.378109
H	-8.402924	7.602019	1.743762
H	-7.273585	8.465238	-0.323849
H	-6.510639	6.932413	0.153219
H	-7.664861	6.960350	-1.188584
H	-7.003505	0.912502	0.940717
H	-8.771014	0.889564	1.080286
H	-7.274819	1.497042	-1.517278
H	-9.021266	1.300472	-1.352640
H	-8.776497	-1.111795	-0.821945
H	-7.845124	-0.770089	-2.295571
H	-7.011722	-0.952566	-0.732872
H	-5.430357	4.730246	3.036084
H	-6.754242	4.593637	4.218863
H	-4.686546	4.122440	5.338213
H	-5.664721	2.653167	5.278938
H	-3.310323	3.305095	3.398372
H	-3.258145	2.178592	4.778353
H	-4.235623	1.808307	3.333355
H	-0.196781	2.792118	-2.849282
H	0.119809	1.164800	-3.478893
H	-2.651329	2.480092	-3.370082
H	-1.582011	2.515482	-4.772645
H	-1.635115	0.009455	-4.940135
H	-2.729181	-0.083762	-3.544349
H	-3.274671	0.699277	-5.033075
H	-1.654175	1.611396	1.196714
H	-2.081050	0.092959	1.912750
H	-0.227740	-0.020241	3.335033
H	0.810847	0.902343	2.303526
H	-1.424766	2.105336	4.030118
H	0.325815	2.226033	4.306898
H	-0.423448	3.030929	2.907341
H	0.971715	3.508921	-1.382396
H	2.119218	2.234464	-1.832132
H	3.118583	4.293710	-0.699778
H	2.301110	3.814843	0.789361
H	4.670889	3.010754	0.796234
H	3.595075	1.595791	0.759439
H	4.403597	2.134700	-0.727472
C	-4.355126	0.826560	0.058189
C	-4.631306	1.286060	-1.388142
O	-5.124634	2.612954	-1.265320
C	-4.457089	3.216582	-0.111954
O	-5.024986	4.205664	0.409880
O	-3.065626	3.424588	-0.611509
C	-2.321951	4.220888	0.298836
H	-3.712936	1.269433	-1.991408
H	-3.400405	0.278974	0.117703
H	-2.804600	5.198553	0.457640
H	-2.214169	3.722900	1.280103
H	-1.323800	4.370842	-0.139693
H	-5.388061	0.672083	-1.897031
H	-5.133088	0.148161	0.442600

77

2t-P1-PO1 ΔE=0.0 SI

C	0.000000	0.000000	0.000000

C	1.433161	0.000000	0.000000
C	2.136144	1.223696	0.000000
C	1.474402	2.433151	0.016143
C	0.064412	2.435989	0.026620
C	-0.668005	1.267674	0.013770
C	2.181479	-1.213844	-0.055978
N	1.671858	-2.396425	0.038048
Co	-0.139872	-2.780908	0.459264
O	0.434886	-2.151941	2.662025
C	-2.162732	1.227937	0.026271
O	-0.751882	-1.046268	-0.030982
C	2.441592	-3.631133	-0.069703
C	1.925839	-4.527801	1.068315
C	2.589249	-5.895818	1.042055
C	4.111879	-5.747329	1.085102
C	4.614608	-4.869145	-0.059890
C	3.954875	-3.489012	-0.026339
N	0.470828	-4.513536	0.945192
C	-0.273740	-5.555243	1.112126
C	-1.699054	-5.554791	1.072679
C	-2.375671	-6.788348	1.191131
C	-3.752307	-6.854959	1.205435
C	-4.492559	-5.658535	1.105524
C	-3.878928	-4.428809	0.997452
C	-2.448510	-4.337957	0.975032
C	-4.623598	-3.136470	0.889664
O	-1.953669	-3.152546	0.887009
O	-0.262908	-3.511266	-1.359894
C	-1.168474	-3.347494	-2.269044
O	-1.086584	-3.783693	-3.422587
O	-2.280480	-2.638799	-1.848508
C	-3.267693	-2.490566	-2.872664
H	-4.263058	-7.812748	1.292307
H	2.027408	3.371201	0.024418
H	2.153067	-4.006907	2.019153
H	2.125130	-4.099991	-1.021789
H	4.241623	-2.960532	0.901600
H	4.301587	-2.886824	-0.881756
H	4.381162	-5.358145	-1.024003
H	5.710745	-4.760157	-0.007112
H	4.403019	-5.290065	2.048988
H	4.584508	-6.742515	1.041193
H	2.289216	-6.421922	0.117124
H	2.249991	-6.497278	1.900647
H	0.201465	-6.521525	1.311861
H	-5.584384	-5.700668	1.113591
H	-1.779319	-7.699510	1.271740
H	-5.710745	-3.299162	0.883192
H	-4.312998	-2.610673	-0.026210
H	-4.350076	-2.472775	1.725100
H	3.262617	-1.109537	-0.197747
H	-0.469589	3.389196	0.047053
H	3.227632	1.193550	-0.009413
H	-2.589452	2.235298	0.131804
H	-2.502310	0.580673	0.847752
H	-2.538071	0.761981	-0.899092
H	-4.071124	-1.894444	-2.418383
H	-2.853519	-1.968499	-3.751408
H	-3.654234	-3.471834	-3.194952
C	-0.327694	-2.832247	3.535790
C	0.492335	-3.019495	4.832427

O	1.331154	-4.298733	4.862514
C	2.634372	-4.167379	4.688846
O	3.329817	-3.158256	4.677420
O	3.160838	-5.447743	4.521869
C	4.592092	-5.455174	4.382877
H	4.922493	-4.718373	3.638763
H	5.083042	-5.228109	5.342052
H	4.853869	-6.470949	4.062319
C	-1.513031	-2.086836	3.912404
H	-0.185288	-3.140819	5.686585
H	1.174213	-2.176952	4.988401
H	-0.566672	-3.885877	3.167010
H	-2.133884	-1.923548	3.032745
H	-2.076071	-2.650977	4.654463
H	-1.219357	-1.126158	4.332852

77

2t-P1C-PO1 ΔE=12.7 SI

C	0.000000	0.000000	0.000000
C	1.433161	0.000000	0.000000
C	2.136144	1.223696	0.000000
C	1.474402	2.433151	0.016143
C	0.064412	2.435989	0.026620
C	-0.668005	1.267674	0.013770
C	2.181479	-1.213844	-0.055978
N	1.671858	-2.396425	0.038048
Co	-0.139872	-2.780908	0.459264
O	0.434886	-2.151941	2.662025
C	-2.162732	1.227937	0.026271
O	-0.751882	-1.046268	-0.030982
C	2.441592	-3.631133	-0.069703
C	1.925839	-4.527801	1.068315
C	2.589249	-5.895818	1.042055
C	4.111879	-5.747329	1.085102
C	4.614608	-4.869145	-0.059890
C	3.954875	-3.489012	-0.026339
N	0.470828	-4.513536	0.945192
C	-0.273740	-5.555243	1.112126
C	-1.699054	-5.554791	1.072679
C	-2.375671	-6.788348	1.191131
C	-3.752307	-6.854959	1.205435
C	-4.492559	-5.658535	1.105524
C	-3.878928	-4.428809	0.997452
C	-2.448510	-4.337957	0.975032
C	-4.623598	-3.136470	0.889664
O	-1.953669	-3.152546	0.887009
O	-0.262908	-3.511266	-1.359894
C	-1.168474	-3.347494	-2.269044
O	-1.086584	-3.783693	-3.422587
O	-2.280480	-2.638799	-1.848508
C	-3.267693	-2.490566	-2.872664
H	-4.263058	-7.812748	1.292307
H	2.027408	3.371201	0.024418
H	2.153067	-4.006907	2.019153
H	2.125130	-4.099991	-1.021789
H	4.241623	-2.960532	0.901600
H	4.301587	-2.886824	-0.881756
H	4.381162	-5.358145	-1.024003
H	5.710745	-4.760157	-0.007112
H	4.403019	-5.290065	2.048988
H	4.584508	-6.742515	1.041193
H	2.289216	-6.421922	0.117124

H	2.249991	-6.497278	1.900647
H	0.201465	-6.521525	1.311861
H	-5.584384	-5.700668	1.113591
H	-1.779319	-7.699510	1.271740
H	-5.710745	-3.299162	0.883192
H	-4.312998	-2.610673	-0.026210
H	-4.350076	-2.472775	1.725100
H	3.262617	-1.109537	-0.197747
H	-0.469589	3.389196	0.047053
H	3.227632	1.193550	-0.009413
H	-2.589452	2.235298	0.131804
H	-2.502310	0.580673	0.847752
H	-2.538071	0.761981	-0.899092
H	-4.071124	-1.894444	-2.418383
H	-2.853519	-1.968499	-3.751408
H	-3.654234	-3.471834	-3.194952
C	-0.561607	-2.254637	3.394989
O	-0.405692	-3.232294	4.568803
C	-1.638452	-3.188909	5.254566
O	-2.031228	-2.411673	3.069786
C	-2.679783	-3.100087	4.123523
O	-0.588327	-0.890253	4.070355
C	0.734442	-0.454038	4.329690
H	-3.594753	-2.563548	4.445161
C	-1.795533	-4.386666	6.056564
H	1.353697	-0.508000	3.416537
H	0.679844	0.591415	4.677589
H	1.228321	-1.063898	5.114439
H	-2.973555	-4.117860	3.787886
H	-1.708128	-2.298799	5.912996
H	-1.038298	-4.401471	6.839058
H	-2.785819	-4.392909	6.509572
H	-1.680455	-5.265166	5.423385

77

2c-P1-PO1 ΔE=8.51 SI

C	0.000000	0.000000	0.000000
C	1.432588	0.000000	0.000000
C	2.136321	1.230444	0.000000
C	1.469861	2.432552	-0.009298
C	0.061253	2.432050	-0.016814
C	-0.673532	1.263244	-0.010398
C	2.197369	-1.197811	0.026141
N	1.729576	-2.408588	0.001814
Co	-0.111152	-2.851532	-0.032678
O	-2.298135	-3.418227	0.655842
C	-2.169712	1.244549	-0.010641
O	-0.751975	-1.057702	0.020070
C	2.611259	-3.588517	0.021095
C	1.770656	-4.733010	0.617675
C	2.521972	-6.050527	0.572337
C	3.828755	-5.907537	1.358768
C	4.680263	-4.765143	0.804181
C	3.919288	-3.434619	0.786113
N	0.520124	-4.644102	-0.120443
C	0.098560	-5.519943	-0.975092
C	-0.849382	-5.206305	-1.996421
C	-0.980725	-3.849439	-2.435801
C	-1.832770	-3.578178	-3.546169
C	-2.498827	-4.623005	-4.163712
C	-2.356463	-5.956147	-3.740821
C	-1.530503	-6.238615	-2.673560

O	-0.326528	-2.834907	-1.926164
C	-1.974050	-2.151992	-3.975845
O	-0.488049	-3.071263	2.288478
H	2.020515	3.372180	-0.010928
H	-2.894710	-6.754370	-4.250163
H	2.814863	-3.858359	-1.033902
H	1.538318	-4.463400	1.662149
H	2.751142	-6.314931	-0.475663
H	1.898395	-6.853750	0.996496
H	3.593400	-5.710764	2.419753
H	4.391669	-6.853069	1.323591
H	4.992633	-5.013950	-0.226089
H	5.600605	-4.651681	1.397853
H	3.687145	-3.106194	1.814981
H	4.552408	-2.662494	0.323599
H	3.282636	-1.066539	0.081574
H	-0.473125	3.384093	-0.025231
H	3.227222	1.205679	0.005856
H	-2.577992	2.262891	-0.032125
H	-2.546037	0.722345	0.882487
H	-2.546286	0.684080	-0.879500
H	0.527057	-6.529006	-0.962976
H	-3.159728	-4.396752	-5.002842
H	-1.400881	-7.266597	-2.331743
H	-2.735405	-2.047020	-4.759160
H	-1.015764	-1.762697	-4.351684
H	-2.242081	-1.527111	-3.111711
C	-3.157732	-2.778120	-0.068574
O	-4.383750	-2.870641	0.057746
O	-2.596589	-1.943114	-1.019121
C	-3.574090	-1.239417	-1.790331
H	-3.000391	-0.643617	-2.513605
H	-4.244226	-1.939436	-2.316807
H	-4.184360	-0.580799	-1.150048
C	0.619789	-2.708883	2.958694
C	0.523082	-1.193456	3.246629
O	1.667548	-0.645104	4.101312
C	2.589568	0.071554	3.483193
O	2.600136	0.530035	2.346625
O	3.635128	0.267181	4.384810
C	4.688509	1.091263	3.856475
H	4.995619	0.751551	2.858464
H	4.369136	2.142709	3.785285
H	5.519562	1.002226	4.566761
H	0.790146	-3.213233	3.967532
C	-0.654002	-0.919668	4.047887
H	0.506151	-0.619089	2.314439
H	1.613662	-2.857196	2.416972
H	-1.540229	-1.275313	3.524400
H	-0.568303	-1.431170	5.005459
H	-0.737494	0.153101	4.215569

77

2c-P1C-PO1 ΔE=14.78 SI

C	0.000000	0.000000	0.000000
C	1.432589	0.000000	0.000000
C	2.136322	1.230444	0.000000
C	1.469862	2.432552	-0.009298
C	0.061254	2.432050	-0.016814
C	-0.673531	1.263244	-0.010398
C	2.197370	-1.197811	0.026141
N	1.729577	-2.408588	0.001814

Co	-0.111151	-2.851532	-0.032678
O	-2.298134	-3.418227	0.655842
C	-3.157731	-2.778120	-0.068574
O	-4.383749	-2.870641	0.057746
C	-2.169711	1.244549	-0.010641
O	-0.751974	-1.057702	0.020070
C	2.611260	-3.588517	0.021095
C	1.770657	-4.733010	0.617676
C	2.521973	-6.050526	0.572338
C	3.828756	-5.907536	1.358769
C	4.680264	-4.765143	0.804181
C	3.919289	-3.434619	0.786113
N	0.520125	-4.644102	-0.120442
C	0.098561	-5.519943	-0.975091
C	-0.849380	-5.206306	-1.996420
C	-0.980724	-3.849440	-2.435801
C	-1.832768	-3.578180	-3.546169
C	-2.498826	-4.623007	-4.163712
C	-2.356461	-5.956149	-3.740821
C	-1.530502	-6.238616	-2.673560
O	-0.326527	-2.834907	-1.926165
C	-1.974049	-2.151994	-3.975846
O	-0.488048	-3.071262	2.288478
O	-2.596588	-1.943114	-1.019121
C	-3.574089	-1.239417	-1.790331
H	2.020516	3.372180	-0.010928
H	-2.894708	-6.754372	-4.250162
H	2.814865	-3.858359	-1.033901
H	1.538318	-4.463399	1.662149
H	2.751143	-6.314931	-0.475662
H	1.898396	-6.853750	0.996497
H	3.593401	-5.710763	2.419754
H	4.391670	-6.853069	1.323592
H	4.992634	-5.013950	-0.226089
H	5.600605	-4.651681	1.397853
H	3.687146	-3.106193	1.814981
H	4.552409	-2.662494	0.323599
H	3.282637	-1.066539	0.081573
H	-0.473124	3.384093	-0.025231
H	3.227223	1.205679	0.005856
H	-2.577991	2.262891	-0.032125
H	-2.546036	0.722345	0.882488
H	-2.546285	0.684080	-0.879500
H	0.527058	-6.529006	-0.962975
H	-3.159726	-4.396754	-5.002843
H	-1.400879	-7.266598	-2.331742
H	-2.735404	-2.047023	-4.759161
H	-1.015762	-1.762699	-4.351685
H	-2.242080	-1.527113	-3.111712
H	-3.000390	-0.643617	-2.513605
H	-4.244224	-1.939436	-2.316807
H	-4.184359	-0.580799	-1.150048
C	-0.940431	-2.005867	2.736879
O	-1.233141	-2.008628	4.244286
C	-2.121336	-0.931709	4.452144
O	-2.259147	-1.516797	2.178312
C	-3.050802	-0.986164	3.225504
O	-0.094573	-0.754949	2.541666
C	1.139165	-0.895176	3.223930
H	-3.442829	0.015742	2.959054
H	-2.668167	-1.076607	5.400662

H	1.638029	-1.840895	2.946466
H	1.778405	-0.042767	2.937999
H	1.009382	-0.887464	4.326097
C	-4.178369	-1.859916	3.485600
H	-1.585117	0.038851	4.486260
H	-4.825437	-1.887885	2.610132
H	-3.817040	-2.864138	3.702162
H	-4.739465	-1.483936	4.339843

77

2c-P1-PO1 ΔE=31.95 SI

C	1.686179	-3.584495	-1.339506
C	0.960811	-2.350642	-1.404784
C	1.084509	-1.566318	-2.601887
C	1.854344	-2.037897	-3.687240
C	2.555324	-3.222043	-3.597389
C	2.465979	-3.980163	-2.412091
O	0.213341	-2.033205	-0.387897
Co	0.080292	-0.104920	0.126389
O	0.256390	-0.701972	1.948972
C	-0.694366	-0.667405	2.832873
C	-2.037376	-0.248546	2.562198
C	-2.983505	-0.197329	3.614462
C	-2.642485	-0.560383	4.898865
C	-1.330173	-1.002911	5.157090
C	-0.366131	-1.069690	4.168344
C	-2.477465	0.121440	1.258273
N	-1.776069	0.141494	0.166233
C	-2.341951	0.615880	-1.118727
C	-1.182094	1.314071	-1.863893
C	-1.601440	1.738708	-3.261652
C	-2.791159	2.699925	-3.151658
C	-3.956066	2.041204	-2.407493
C	-3.547288	1.544374	-1.013296
N	-0.092297	0.350633	-1.740014
C	0.350156	-0.350947	-2.738497
C	1.028356	-1.551266	4.432822
C	1.581217	-4.379863	-0.075274
O	2.324321	-0.127382	-0.042815
C	2.993071	0.908497	-0.034627
O	3.028813	1.844539	0.908162
C	2.141910	1.601646	2.051911
C	0.748308	2.156258	1.761914
O	0.238243	1.786410	0.519255
O	3.886511	1.222518	-0.983523
C	3.897355	0.305784	-2.114103
H	-3.376134	-0.517404	5.703554
H	3.168509	-3.571110	-4.427693
H	-2.601889	-0.289626	-1.702308
H	-0.886510	2.189761	-1.261740
H	-1.899566	0.856457	-3.856939
H	-0.754499	2.222034	-3.776870
H	-2.474486	3.610286	-2.612148
H	-3.113250	3.017041	-4.156612
H	-4.326393	1.185578	-3.000978
H	-4.796795	2.746823	-2.309919
H	-3.275985	2.396415	-0.364938
H	-4.403264	1.027847	-0.550751
H	-3.529292	0.412378	1.183179
H	-1.059984	-1.306237	6.171840
H	-3.998756	0.134905	3.387702
H	1.133311	-1.908087	5.466086

H	1.764808	-0.751048	4.263611
H	1.287961	-2.366631	3.741146
H	0.125821	-0.008720	-3.756251
H	3.030127	-4.912693	-2.333261
H	1.906140	-1.433786	-4.596478
H	2.231777	-5.264299	-0.105533
H	0.542343	-4.703071	0.094940
H	1.851884	-3.752702	0.788286
C	0.823544	3.604268	1.751482
H	2.109400	0.522996	2.242814
H	3.822224	-0.732795	-1.775452
H	3.048402	0.538399	-2.766839
H	4.846055	0.494992	-2.625563
H	0.099486	1.872915	2.619598
H	2.622293	2.128786	2.888051
H	1.218246	3.954123	2.704232
H	1.480599	3.927821	0.945558
H	-0.171707	4.018364	1.596820
C	3.727856	0.733941	5.130276
O	2.624866	0.609750	5.983887
C	1.524771	1.498075	5.731986
O	1.391993	1.892514	4.561598
O	0.867806	1.694322	6.742747
H	4.375436	-0.108521	5.401618
H	3.435184	0.661645	4.079192
H	4.230198	1.689666	5.321830

77

2c-P1C-PO1 ΔE=35.82 SI

C	2.441993	-0.110763	-3.656752
C	1.086709	0.089238	-4.334081
O	0.161775	-0.509635	-3.409221
C	0.715850	-0.398808	-2.135871
O	2.113750	-0.258306	-2.265968
O	0.307835	-1.252156	-1.251204
Co	-0.265016	0.021094	0.125532
O	0.275879	1.048784	-1.492779
C	-0.764546	1.769659	-2.175859
N	1.499110	0.231558	0.791777
C	1.968622	-1.057400	1.272515
C	0.833072	-1.510759	2.219999
C	1.084708	-2.932659	2.712267
C	2.467413	-3.026990	3.372361
C	3.584208	-2.538445	2.445264
C	3.320086	-1.105163	1.966134
N	-0.424333	-1.291643	1.481704
C	-1.486833	-2.004557	1.690248
C	-2.733160	-1.888001	1.009662
C	-3.783083	-2.750581	1.411870
C	-5.012779	-2.718350	0.793254
C	-5.218433	-1.813139	-0.267416
C	-4.227336	-0.954608	-0.704243
C	-2.945562	-0.960389	-0.062548
C	-4.430182	-0.004252	-1.844110
O	-2.062320	-0.120526	-0.506523
C	2.043159	1.375327	1.043589
C	1.329668	2.612385	0.914789
C	-0.099149	2.609970	1.033195
C	-0.780344	3.864335	1.024128
C	-0.049506	5.035399	0.891024
C	1.352853	5.034361	0.783220
C	2.029756	3.829567	0.813329

O	-0.825204	1.528380	1.177103
C	-2.273377	3.851367	1.151833
H	-5.815009	-3.383423	1.111872
H	1.896201	5.973616	0.680917
H	0.830718	-0.805459	3.075601
H	1.960648	-1.730851	0.397085
H	3.324582	-0.419028	2.832595
H	4.109461	-0.776221	1.270508
H	3.651954	-3.205463	1.567243
H	4.555764	-2.588980	2.962225
H	2.468374	-2.416710	4.293760
H	2.655523	-4.067819	3.681184
H	1.019414	-3.618864	1.849074
H	0.314772	-3.236918	3.438631
H	-1.439420	-2.785904	2.456733
H	-6.188869	-1.789820	-0.769295
H	-3.597235	-3.449099	2.230576
H	-5.449248	-0.082073	-2.247072
H	-3.711548	-0.216197	-2.651472
H	-4.250153	1.033293	-1.522570
H	3.070443	1.407542	1.425378
H	-0.586864	5.986480	0.864185
H	3.119170	3.805440	0.739382
H	-2.700252	4.837267	0.923726
H	-2.572822	3.564446	2.173049
H	-2.711024	3.092607	0.485827
H	3.113780	0.749565	-3.784894
H	0.989115	-0.434065	-5.293325
H	-1.622758	1.125173	-2.395821
H	-1.066926	2.582274	-1.503287
H	-0.352195	2.201087	-3.099890
C	3.101358	-1.323078	-4.101757
H	0.848461	1.156636	-4.468887
H	4.059489	-1.422581	-3.593815
H	2.475666	-2.183261	-3.868271
H	3.264126	-1.273883	-5.177400
C	-0.882480	0.796812	4.983504
O	-0.239668	-0.276875	5.632183
C	-0.502168	-1.560567	5.107418
O	-1.275930	-1.621140	4.131860
O	0.142374	-2.445302	5.662159
H	-0.964185	1.569311	5.752470
H	-0.259013	1.133111	4.151374
H	-1.875217	0.516334	4.610825

184

3t-P1-EO ΔE=15.75 SI

C	-6.634394	0.397307	-0.808474
C	-5.524022	0.391677	-1.844624
C	-6.120178	0.408581	-3.261618
C	-7.473530	1.154102	-3.283317
C	-7.494136	2.290160	-2.255231
C	-7.380750	1.738367	-0.815144
N	-4.626869	-0.733375	-1.546138
Co	-4.549939	-1.144994	0.307618
O	-3.287590	0.336033	0.380536
N	-6.044327	0.010149	0.475434
C	-6.706064	0.229044	1.564951
C	-6.389479	-0.314861	2.846243
C	-7.317489	-0.031022	3.868685
C	-7.242861	-0.631370	5.110754
C	-6.150410	-1.489077	5.347631

C	-5.184962	-1.771741	4.393265
C	-5.288016	-1.199394	3.083694
C	-8.354419	-0.448128	6.113984
C	-9.748051	-0.661576	5.496107
C	-9.912710	-2.045857	4.816602
C	-9.873108	-1.917648	3.297350
N	-10.123656	-3.218894	2.540408
C	-10.105747	-2.947421	1.035564
C	-8.908973	-2.205316	0.469314
C	-8.886030	-2.393752	-1.055977
C	-9.956931	-1.588720	-1.790445
C	-4.057342	-2.719029	4.674763
O	-4.389987	-1.525331	2.200305
C	-3.816192	-1.184398	-2.446594
C	-2.782771	-2.149703	-2.238656
C	-1.973310	-2.495566	-3.341536
C	-0.953680	-3.425245	-3.249351
C	-0.821165	-4.128045	-2.028764
C	-1.587679	-3.836038	-0.917146
C	-2.541335	-2.758887	-0.960933
C	0.103303	-3.524256	-4.311852
C	1.395390	-2.774202	-3.909211
C	1.175608	-1.313169	-3.455695
C	0.865151	-1.250488	-1.963121
N	0.615891	0.124997	-1.364546
C	1.602247	1.168885	-1.846060
C	3.060149	0.898648	-1.508003
C	3.957961	1.910816	-2.227485
C	5.434031	1.727291	-1.878402
C	-1.472007	-4.592208	0.370043
O	-3.109514	-2.432224	0.147727
O	-5.890021	-2.611670	0.288988
C	-5.900143	-3.703007	-0.396254
O	-5.127460	-3.642146	-1.538202
C	-4.925733	-4.893843	-2.206815
O	-6.557544	-4.712952	-0.114998
C	-0.798005	0.627001	-1.632661
C	-1.183963	1.011743	-3.051952
C	-2.444162	1.890369	-2.953242
C	-2.122865	3.351725	-2.641907
C	0.749589	0.012173	0.159803
C	-0.259268	-0.890698	0.844301
C	-0.075290	-0.774011	2.361761
C	-1.036069	-1.690046	3.116292
C	-9.135393	-4.308555	2.943148
C	-7.661120	-3.924844	2.973166
C	-6.841368	-4.973913	3.735121
C	-7.119765	-5.023926	5.238049
C	-11.534964	-3.673759	2.879805
C	-12.068144	-4.873568	2.110891
C	-13.452970	-5.253895	2.647226
C	-14.070440	-6.423080	1.882027
O	-11.673231	-0.209256	1.425753
C	-10.733451	0.583362	1.165035
O	-10.813887	1.310269	-0.064904
C	-12.047569	1.140786	-0.769896
O	-9.684310	0.842586	1.803465
H	-7.333060	-0.411040	-1.071002
H	-4.924273	1.303095	-1.663136
H	-6.258517	-0.631961	-3.600668
H	-5.415500	0.892861	-3.957162

H	-7.669121	1.536830	-4.297512
H	-8.288740	0.450635	-3.050652
H	-8.419239	2.879286	-2.348534
H	-6.656147	2.977252	-2.470590
H	-6.833575	2.451576	-0.175244
H	-8.382909	1.609741	-0.377882
H	-7.642287	0.804281	1.510543
H	-6.068968	-1.976813	6.324322
H	-8.161816	0.609115	3.604262
H	-3.863880	-3.354870	3.798494
H	-4.278692	-3.349114	5.548482
H	-3.122236	-2.173248	4.875177
H	-3.909003	-0.798302	-3.469791
H	-0.064466	-4.915689	-1.951916
H	-2.146411	-1.981268	-4.292428
H	-2.442470	-5.044848	0.630458
H	-1.231258	-3.915364	1.202221
H	-0.710252	-5.381691	0.304924
H	-4.132651	-4.705105	-2.941275
H	-4.606058	-5.667532	-1.495642
H	-5.846110	-5.229466	-2.705914
H	0.362581	-4.575802	-4.526470
H	-0.279837	-3.091453	-5.252079
H	1.899202	-3.331969	-3.099746
H	2.089446	-2.780077	-4.766585
H	2.091438	-0.739288	-3.670463
H	0.363634	-0.866688	-4.047052
H	-0.022563	-1.838262	-1.714770
H	1.709631	-1.675898	-1.400278
H	-0.908899	1.481612	-0.958742
H	-1.482632	-0.143891	-1.264890
H	0.664126	1.043756	0.529312
H	1.771993	-0.345678	0.341883
H	1.478673	1.239329	-2.933045
H	1.269440	2.114359	-1.392146
H	-8.212883	-1.162849	6.943490
H	-8.313570	0.561824	6.561835
H	-10.504334	-0.550614	6.290281
H	-9.958968	0.131484	4.760425
H	-9.115379	-2.719025	5.173052
H	-10.871840	-2.477033	5.140417
H	-10.628300	-1.211191	2.910590
H	-8.899246	-1.554360	2.962105
H	-9.469415	-4.634363	3.938707
H	-9.291463	-5.139286	2.240945
H	-12.168354	-2.789845	2.708589
H	-11.536240	-3.900640	3.953376
H	-10.173889	-3.940785	0.570169
H	-11.023565	-2.371199	0.846787
H	-12.221810	0.088847	-1.037286
H	-12.900428	1.486329	-0.165832
H	-11.963645	1.748322	-1.681336
H	3.227740	0.984784	-0.423309
H	3.354359	-0.122323	-1.805995
H	3.817429	1.813248	-3.318842
H	3.635988	2.932429	-1.959287
H	5.601665	1.855621	-0.797667
H	5.784160	0.720676	-2.156280
H	6.061514	2.460307	-2.405653
H	-1.297018	-0.601944	0.609306
H	-0.124159	-1.938428	0.532637

H	-0.256687	0.276164	2.650645
H	0.970467	-1.001813	2.642956
H	-2.071224	-1.551589	2.772105
H	-0.779701	-2.749026	2.957637
H	-0.992824	-1.496334	4.198610
H	-0.395216	1.572399	-3.577854
H	-1.399645	0.107731	-3.637254
H	-3.014539	1.827374	-3.893809
H	-3.083565	1.478569	-2.157231
H	-1.484128	3.438692	-1.751796
H	-1.585800	3.826856	-3.478605
H	-3.038237	3.931686	-2.451364
H	-7.256612	-3.867814	1.955543
H	-7.512957	-2.949472	3.456079
H	-6.995430	-5.968969	3.279875
H	-5.781352	-4.726906	3.570237
H	-8.151506	-5.337060	5.466967
H	-6.448218	-5.735602	5.742607
H	-6.963045	-4.031356	5.686140
H	-12.154397	-4.643830	1.038228
H	-11.393292	-5.739381	2.211012
H	-13.372992	-5.509794	3.718448
H	-14.118708	-4.375454	2.583875
H	-13.434248	-7.319255	1.948426
H	-15.061442	-6.681677	2.282530
H	-14.191726	-6.174922	0.816371
H	-7.961104	-2.578653	0.864052
H	-8.977406	-1.136838	0.722325
H	-7.886178	-2.108378	-1.417378
H	-8.970135	-3.470276	-1.288391
H	-9.865235	-1.698112	-2.882703
H	-10.972828	-1.908794	-1.510064
H	-9.881190	-0.520593	-1.534409
C	-3.311707	1.049538	1.577492
C	-2.820350	2.463173	1.367585
O	-1.380628	2.397554	1.023461
C	-0.736156	3.511731	0.680937
O	0.415999	3.490049	0.269479
O	-1.487833	4.625851	0.822439
C	-0.812505	5.841345	0.412864
H	-3.342065	2.938854	0.525619
H	-4.329312	1.172512	1.999728
H	-0.573739	5.793706	-0.656641
H	-1.526172	6.644557	0.617315
H	0.109112	5.974050	0.991680
H	-2.921456	3.071095	2.278339
H	-2.709985	0.578259	2.383123

184

3t-P1-EO ΔE=30.99 SI

C	-2.391081	-1.338526	4.547382
C	-3.111088	-0.022998	4.864397
C	-2.986138	0.342858	6.337561
C	-1.495912	0.343242	6.791830
C	-0.529458	0.008205	5.650660
C	-0.902356	-1.306329	4.960458
N	-4.462191	-0.191165	4.336496
Co	-4.420140	-1.024120	2.632928
O	-3.821490	0.695630	1.926667
N	-2.672991	-1.598027	3.126041
C	-1.923921	-2.393520	2.440969
C	-2.166144	-2.797404	1.092597

C	-1.175934	-3.589847	0.491108
C	-1.240306	-3.964254	-0.840572
C	-2.344860	-3.510925	-1.585978
C	-3.368952	-2.753003	-1.027737
C	-3.332505	-2.414684	0.361420
C	-0.059021	-4.663649	-1.468030
C	1.182104	-3.754569	-1.385808
C	0.964382	-2.457759	-2.181812
C	1.708814	-1.246682	-1.614112
N	0.798990	-0.091488	-1.204727
C	1.680265	1.026864	-0.680854
C	2.638132	1.689259	-1.660229
C	3.646386	2.542824	-0.869978
C	4.660434	1.705416	-0.085647
C	-4.558108	-2.310058	-1.826754
O	-4.367556	-1.803864	0.865226
C	-5.535556	0.092275	4.993855
C	-6.869180	-0.076659	4.502524
C	-7.939651	0.298601	5.345977
C	-9.260265	0.124054	4.976541
C	-9.511730	-0.589420	3.780006
C	-8.503334	-0.958816	2.909212
C	-7.147769	-0.565689	3.184632
C	-10.377287	0.793180	5.724817
C	-10.918668	2.032420	4.970366
C	-9.813024	2.998888	4.482962
C	-9.430651	2.672116	3.045522
N	-8.270959	3.461205	2.467958
C	-8.500166	4.959061	2.550210
C	-9.796127	5.472791	1.939299
C	-9.873649	7.001007	2.084635
C	-8.839320	7.750148	1.242244
C	-8.741587	-1.809919	1.703195
O	-6.282992	-0.656640	2.225645
O	-5.170862	-2.619224	3.545152
C	-5.389350	-3.662577	2.798498
O	-4.206042	-4.278387	2.427926
C	-4.348526	-5.279520	1.408389
O	-6.479697	-4.131204	2.472595
C	-6.955076	3.077575	3.186480
C	-6.477259	4.033787	4.271010
C	-5.227907	3.481465	4.973788
C	-4.033765	3.252325	4.049086
C	-8.130081	3.105892	0.987852
C	-7.892414	1.641940	0.671348
C	-7.526121	1.506208	-0.813343
C	-7.353756	0.047748	-1.238989
C	-0.126317	0.371948	-2.317189
C	0.411560	0.375428	-3.742430
C	-0.761220	0.432741	-4.737290
C	-1.597958	-0.849867	-4.768547
C	-0.095478	-0.593355	-0.049531
C	-0.825345	0.497312	0.722045
C	-0.127727	1.016843	1.985028
C	-0.861734	2.229797	2.550906
O	2.835058	-0.845045	1.425641
C	2.102516	-1.364367	2.312586
O	2.215682	-0.857128	3.633010
C	3.143287	0.220848	3.779399
O	1.232586	-2.260311	2.205730
H	-2.933643	-2.131719	5.095195

H	-2.649520	0.755824	4.236391
H	-3.563093	-0.387188	6.930846
H	-3.439260	1.331414	6.512811
H	-1.236334	1.320208	7.229559
H	-1.364849	-0.402702	7.593376
H	0.502159	-0.067757	6.027575
H	-0.524150	0.825440	4.912502
H	-0.234325	-1.459670	4.100121
H	-0.703873	-2.145686	5.647214
H	-0.991136	-2.759510	2.882488
H	-2.408283	-3.767001	-2.648870
H	-0.298806	-3.838203	1.092096
H	-5.489581	-2.711007	-1.395042
H	-4.478557	-2.636419	-2.873595
H	-4.662922	-1.214346	-1.800203
H	-5.441691	0.479323	6.014889
H	-10.544118	-0.855288	3.529903
H	-7.703547	0.763891	6.307933
H	-9.804730	-2.071484	1.604387
H	-8.137055	-2.729526	1.808262
H	-8.399112	-1.319045	0.785010
H	-4.854799	-6.172970	1.801538
H	-4.927822	-4.884693	0.562405
H	-3.328127	-5.517511	1.083675
H	-11.215226	0.097436	5.904321
H	-10.010876	1.113967	6.715128
H	-11.513187	1.694242	4.103447
H	-11.616789	2.574732	5.629153
H	-10.168674	4.039051	4.559240
H	-8.934316	2.897507	5.138011
H	-9.143086	1.618093	2.961858
H	-10.286529	2.839749	2.374219
H	-6.199884	2.996246	2.400668
H	-7.119769	2.073755	3.595172
H	-7.292771	3.722831	0.633707
H	-9.053415	3.449090	0.503261
H	-8.479189	5.224924	3.613003
H	-7.620427	5.401178	2.066288
H	0.150623	-5.614754	-0.949324
H	-0.280666	-4.915470	-2.520070
H	1.365446	-3.504016	-0.327811
H	2.081235	-4.281889	-1.747493
H	1.250201	-2.614059	-3.235064
H	-0.116150	-2.253661	-2.199488
H	2.256468	-1.482945	-0.685001
H	2.421708	-0.835521	-2.336689
H	-0.997362	-0.291751	-2.261340
H	-0.468243	1.376874	-2.031275
H	0.540137	-1.200681	0.612935
H	-0.813837	-1.265547	-0.529184
H	0.997926	1.786871	-0.281237
H	2.220546	0.557625	0.157204
H	3.168750	0.458254	4.851544
H	4.147279	-0.061146	3.429911
H	2.818953	1.109427	3.214660
H	-9.866211	5.221644	0.869584
H	-10.665402	5.020020	2.442668
H	-10.886809	7.321422	1.789963
H	-9.762135	7.272967	3.149391
H	-8.971490	8.837816	1.338456
H	-7.807472	7.514471	1.541131

H	-8.944821	7.491906	0.176708
H	-7.076991	1.217796	1.274712
H	-8.790955	1.041984	0.885422
H	-6.588973	2.062239	-0.988655
H	-8.298293	1.991563	-1.438901
H	-8.315649	-0.486548	-1.213675
H	-6.960359	-0.021762	-2.264360
H	-6.662696	-0.481032	-0.566620
H	-6.223523	5.009689	3.828885
H	-7.257119	4.196737	5.031527
H	-4.956889	4.194894	5.771233
H	-5.490708	2.539344	5.476979
H	-3.788987	4.171839	3.492353
H	-3.141402	2.985853	4.634155
H	-4.204089	2.435679	3.327802
H	1.088423	1.225482	-3.909184
H	0.984892	-0.541400	-3.943695
H	-1.406258	1.298095	-4.500256
H	-0.349911	0.623415	-5.742160
H	-0.968528	-1.720291	-5.011333
H	-2.092013	-1.057329	-3.807839
H	-2.388817	-0.781867	-5.530416
H	-1.088423	1.337066	0.054098
H	-1.792006	0.084248	1.047743
H	-0.115504	0.201157	2.722779
H	0.929104	1.259407	1.803473
H	-1.923588	1.985535	2.697101
H	-0.430200	2.557916	3.509028
H	-0.812304	3.086961	1.855629
H	2.090392	2.328748	-2.370978
H	3.200514	0.948923	-2.250118
H	4.174848	3.202902	-1.578328
H	3.094205	3.208450	-0.181391
H	5.352958	2.354764	0.470792
H	4.179516	1.025210	0.633263
H	5.261051	1.087434	-0.772971
C	-4.126864	0.911863	0.590315
C	-3.787337	2.331667	0.186052
O	-4.840691	3.248667	0.677703
C	-4.565792	4.527048	0.932424
O	-5.429248	5.307280	1.307852
O	-3.268046	4.851555	0.740087
C	-2.954294	6.222637	1.089288
H	-2.831770	2.639737	0.618153
H	-3.541511	0.274228	-0.112633
H	-3.554785	6.914192	0.486193
H	-3.153609	6.393043	2.153921
H	-1.888159	6.333690	0.871946
H	-3.770023	2.444492	-0.910131
H	-5.185062	0.707839	0.346157

187

3t-P1-PO1 ΔE=0.00 SI

C	3.338046	-0.042285	-4.489297
C	3.204346	1.056839	-3.580323
C	4.348921	1.682010	-3.050816
C	5.627405	1.230468	-3.316317
C	5.755462	0.141935	-4.201460
C	4.668384	-0.480750	-4.792785
C	1.951432	1.471060	-3.036478
N	0.809209	0.917348	-3.255743
Co	0.488636	-0.474020	-4.516029

O	0.263737	0.937769	-5.776371
C	-0.239187	0.596168	-7.032447
C	-1.545189	1.308443	-7.349735
O	-2.647985	0.989122	-6.428922
C	-3.302050	-0.176668	-6.535821
O	-2.790483	-0.984817	-7.492570
C	-3.475064	-2.257908	-7.610028
C	6.821589	1.845344	-2.634412
C	6.700178	1.906714	-1.104200
C	6.626478	0.538644	-0.400467
C	5.221037	-0.064225	-0.364814
N	5.089331	-1.407880	0.377114
C	5.152658	-2.551881	-0.625795
C	6.361426	-2.575176	-1.539726
C	6.196176	-3.712918	-2.553951
C	7.319518	-3.732384	-3.587533
C	4.834935	-1.646145	-5.719431
O	2.348375	-0.694979	-5.022441
C	-0.399866	1.309627	-2.521999
C	-1.571397	1.014609	-3.469323
C	-2.930100	1.272222	-2.839094
C	-2.979105	2.720180	-2.337731
C	-1.826999	3.014105	-1.372024
C	-0.459502	2.746406	-2.015353
N	-1.310621	-0.326111	-3.978325
C	-2.168629	-1.288043	-3.969206
C	-1.929569	-2.580028	-4.542374
C	-2.868796	-3.593995	-4.277079
C	-2.749778	-4.863137	-4.816413
C	-1.684442	-5.088217	-5.711674
C	-0.758490	-4.112778	-6.045353
C	-0.827811	-2.819824	-5.428548
C	-3.658558	-5.984250	-4.397045
C	-3.038966	-6.890375	-3.314313
C	-2.616604	-6.138509	-2.034644
C	-1.150621	-5.728489	-2.104825
N	-0.631728	-4.841026	-0.987163
C	-0.883786	-5.409492	0.407778
C	-2.257625	-5.190956	1.032064
C	-2.177431	-5.493717	2.540469
C	-1.496116	-4.380764	3.339540
C	0.314766	-4.376561	-7.064092
O	0.075102	-1.937211	-5.737944
O	0.766266	-1.972847	-3.128548
C	1.961874	-2.013387	-2.668534
O	2.473917	-1.300990	-1.796073
O	2.788930	-3.027663	-3.155384
C	2.292865	-3.745664	-4.302863
C	0.882177	-4.695352	-1.169285
C	1.685624	-5.986591	-1.154703
C	3.186545	-5.660386	-1.123826
C	4.042825	-6.909120	-1.319518
C	-1.229015	-3.455933	-1.159300
C	-0.736723	-2.410551	-0.167687
C	-1.675009	-1.200405	-0.197474
C	-3.046571	-1.427508	0.437129
C	6.128464	-1.544801	1.466499
C	6.105219	-2.859208	2.230582
C	6.884873	-2.726456	3.547249
C	6.161480	-1.879463	4.597577
C	3.675323	-1.503821	0.958503

C	3.339152	-0.531580	2.080866
C	1.844070	-0.171100	2.005818
C	1.507028	0.732168	0.812926
O	-4.254000	-0.443459	-5.824751
O	3.518758	-4.530788	2.085205
C	2.267847	-4.583653	2.042878
O	1.662472	-5.855844	2.266490
C	2.592762	-6.913978	2.524019
O	1.421841	-3.687536	1.795910
H	-1.436625	1.668467	-4.350972
H	-0.470736	0.618263	-1.665120
H	-0.294350	3.431596	-2.866428
H	0.335127	2.930893	-1.275420
H	-1.933540	2.374928	-0.475891
H	-1.873853	4.059306	-1.024530
H	-2.914040	3.402593	-3.204252
H	-3.947447	2.915258	-1.849089
H	-3.097282	0.589287	-1.987929
H	-3.719647	1.087837	-3.585455
H	-3.157778	-1.116890	-3.533077
H	-1.585506	-6.076869	-6.172896
H	-3.711245	-3.357886	-3.620363
H	-0.107888	-4.379042	-8.083091
H	0.791553	-5.354810	-6.901118
H	1.076241	-3.588411	-7.027754
H	1.999205	2.297518	-2.318263
H	6.758105	-0.230526	-4.432188
H	4.196428	2.512726	-2.357026
H	5.883556	-1.763022	-6.025837
H	4.206999	-1.520919	-6.614771
H	4.506953	-2.576219	-5.232166
H	3.066660	-4.486423	-4.544031
H	1.343202	-4.256643	-4.085742
H	2.133177	-3.051314	-5.133788
H	7.726103	1.269343	-2.896052
H	6.990574	2.870163	-3.012444
H	7.583258	2.439069	-0.711053
H	5.827199	2.518603	-0.818405
H	7.341412	-0.145941	-0.883060
H	6.974049	0.688220	0.632633
H	4.549291	0.633657	0.150615
H	4.796519	-0.240585	-1.359942
H	3.538634	-2.541393	1.294808
H	3.031928	-1.346868	0.087558
H	4.242323	-2.429514	-1.230466
H	5.057986	-3.473280	-0.036555
H	7.109819	-1.403676	0.998375
H	5.955101	-0.696261	2.141916
H	-4.601694	-5.563727	-4.006808
H	-3.927216	-6.607769	-5.267183
H	-3.773218	-7.668806	-3.047816
H	-2.166839	-7.420247	-3.737487
H	-3.270613	-5.264124	-1.894124
H	-2.763772	-6.798745	-1.168514
H	-0.533369	-6.634957	-2.085448
H	-0.938421	-5.184229	-3.036591
H	-0.963726	-3.143182	-2.178149
H	-2.315608	-3.585592	-1.104934
H	-0.112880	-4.947059	1.040510
H	-0.657446	-6.482592	0.341685
H	0.997643	-4.173877	-2.122697

H	1.211437	-4.042222	-0.349324
H	3.197177	-6.705922	3.419379
H	1.989253	-7.818694	2.679267
H	3.278397	-7.060128	1.676639
H	5.079414	-3.196575	2.441876
H	6.564486	-3.649949	1.615826
H	7.042061	-3.739709	3.951126
H	7.891429	-2.311099	3.355104
H	6.015453	-0.839541	4.267446
H	5.169377	-2.303747	4.814893
H	6.731463	-1.843773	5.538153
H	6.422630	-1.633152	-2.106522
H	7.305651	-2.700836	-0.979831
H	5.224819	-3.578554	-3.056352
H	6.149515	-4.679380	-2.023981
H	7.346953	-2.783208	-4.143582
H	8.302462	-3.875003	-3.110309
H	7.177048	-4.543501	-4.316781
H	3.564878	-0.988716	3.057188
H	3.929982	0.400114	2.019628
H	1.272444	-1.112979	1.953131
H	1.552862	0.329194	2.944627
H	2.101246	1.661979	0.844711
H	1.696072	0.237240	-0.152290
H	0.446084	1.025282	0.834468
H	-0.653399	-2.809040	0.853289
H	0.269162	-2.079460	-0.462511
H	-1.174957	-0.367085	0.320531
H	-1.797655	-0.885816	-1.243920
H	-2.946952	-1.720598	1.493682
H	-3.654434	-0.510288	0.399566
H	-3.621129	-2.212933	-0.078341
H	-2.585840	-4.149344	0.910979
H	-3.023630	-5.826206	0.566627
H	-3.201992	-5.653208	2.916561
H	-1.636454	-6.444627	2.693532
H	-1.444552	-4.643425	4.407292
H	-0.469320	-4.191588	2.990326
H	-2.068207	-3.442481	3.253719
H	1.477594	-6.592823	-2.052761
H	1.438992	-6.590009	-0.266944
H	3.411199	-4.919464	-1.905660
H	3.430175	-5.190238	-0.158201
H	5.114708	-6.672301	-1.245171
H	3.818356	-7.670183	-0.555160
H	3.868009	-7.364709	-2.307800
H	-1.870558	1.098471	-8.380766
C	0.688169	1.001077	-8.070984
H	-3.466611	-2.784066	-6.650101
H	-4.509643	-2.096644	-7.939284
H	-2.904847	-2.814562	-8.359531
H	-1.430594	2.392311	-7.208467
H	-0.376904	-0.489240	-7.151215
H	0.283944	0.728909	-9.044867
H	0.834182	2.079348	-8.027022
H	1.642858	0.499074	-7.921041

187

3t-P1C-PO1 ΔE=15.13 SI

O	-3.201573	-1.311703	0.692865
C	-4.596325	-1.732154	0.783924
O	-4.523876	-3.196538	0.841791

C	-3.192850	-3.590047	1.199478
C	-2.458334	-2.263872	1.431165
O	-5.288066	-1.281702	1.807534
Co	-5.296279	0.457027	2.812081
O	-5.471805	2.123061	3.795299
C	-4.621912	2.566273	4.673624
O	-4.021038	1.968114	5.568887
O	-5.150533	-1.509420	-0.503140
C	-5.580301	-0.156573	-0.706359
N	-3.677624	-0.064623	3.634250
C	-2.492275	0.255084	3.230930
C	-2.179621	1.016179	2.065835
C	-0.846544	0.981501	1.601617
C	-0.463217	1.597884	0.426825
C	-1.425947	2.398848	-0.229156
C	-2.745549	2.463845	0.175093
C	-3.185046	1.655548	1.277786
C	0.852496	1.286893	-0.236995
C	0.631596	0.642475	-1.623146
C	-0.375624	-0.517064	-1.606564
C	-0.722522	-0.938838	-3.032851
N	-2.123875	-1.481240	-3.213230
C	-3.171022	-0.467372	-2.758802
C	-2.995327	0.924250	-3.360807
C	-4.344177	1.622041	-3.596511
C	-5.180084	0.966300	-4.697042
C	-3.752889	3.335705	-0.513213
O	-4.460878	1.558796	1.471163
C	-3.901109	-1.062087	4.688064
C	-5.254747	-0.733246	5.328996
C	-5.664135	-1.797531	6.336329
C	-4.573431	-1.932678	7.406814
C	-3.212310	-2.243254	6.779942
C	-2.810269	-1.185725	5.744906
N	-6.165795	-0.499376	4.208807
C	-7.392095	-0.896121	4.195563
C	-8.331348	-0.661140	3.140927
C	-9.534440	-1.394498	3.157202
C	-10.483592	-1.280790	2.154683
C	-10.262037	-0.294788	1.168526
C	-9.110159	0.472252	1.116072
C	-8.065024	0.261945	2.078301
C	-11.590276	-2.294923	2.025711
C	-11.074957	-3.576915	1.328103
C	-10.678066	-3.325326	-0.139577
C	-9.530118	-4.173839	-0.693246
N	-8.171136	-3.508870	-0.606984
C	-7.096474	-4.339622	-1.313890
C	-7.230250	-5.857550	-1.255803
C	-5.885094	-6.501359	-1.634930
C	-4.878320	-6.463785	-0.483683
C	-8.901528	1.520687	0.065224
O	-6.970535	0.939416	1.935229
C	-8.168973	-2.147332	-1.307921
C	-8.863549	-2.092220	-2.654205
C	-8.532769	-0.754883	-3.329065
C	-9.126564	-0.663994	-4.732374
C	-7.770098	-3.222352	0.832673
C	-7.386675	-4.429945	1.666861
C	-7.016130	-3.987120	3.085363
C	-6.534240	-5.155605	3.940636

C	-2.434091	-1.810000	-4.684270
C	-1.258697	-2.120625	-5.603228
C	-1.788094	-2.708325	-6.923182
C	-2.291888	-4.147155	-6.781658
C	-2.361586	-2.724310	-2.365848
C	-1.532420	-3.925591	-2.784815
C	-2.072057	-5.207838	-2.149203
C	-1.316959	-6.445634	-2.628349
O	-4.415742	3.931111	4.585131
C	-4.956567	4.572884	3.408523
O	-5.614421	-2.257150	-3.570913
C	-5.531475	-3.385178	-4.106865
O	-4.546086	-4.148840	-4.259110
O	-6.797790	-3.854185	-4.582970
C	-6.754467	-5.121206	-5.251024
H	-5.115719	0.249143	5.821671
H	-4.025353	-2.027785	4.156478
H	-2.676919	-0.196991	6.216193
H	-1.852160	-1.477389	5.285820
H	-3.256815	-3.233161	6.287807
H	-2.438065	-2.310128	7.561834
H	-4.506901	-0.985535	7.970460
H	-4.851670	-2.719648	8.126821
H	-5.807985	-2.763677	5.818631
H	-6.619516	-1.528697	6.815282
H	-7.760150	-1.480422	5.046070
H	-11.028127	-0.133058	0.403771
H	-9.688504	-2.119796	3.961697
H	-8.053061	1.268783	-0.588374
H	-8.649952	2.485661	0.532634
H	-9.800799	1.641649	-0.554701
H	-1.634184	-0.126075	3.794385
H	-1.125788	2.973400	-1.110705
H	-0.119245	0.385808	2.160701
H	-3.981392	4.218324	0.108326
H	-4.705394	2.808088	-0.653964
H	-3.380028	3.689243	-1.484352
H	-6.053597	4.608528	3.450361
H	-4.539836	5.587665	3.417652
H	-4.656493	4.032633	2.503177
H	1.470501	2.193125	-0.368091
H	1.428019	0.599991	0.406312
H	0.240932	1.417981	-2.305861
H	1.598408	0.314876	-2.044471
H	0.028680	-1.369109	-1.035289
H	-1.273868	-0.182526	-1.068846
H	-0.650656	-0.075209	-3.706837
H	-0.031763	-1.704290	-3.405125
H	-3.432465	-2.941568	-2.489254
H	-2.193015	-2.421998	-1.324533
H	-3.126143	-0.446534	-1.664810
H	-4.120708	-0.947878	-3.037986
H	-3.152028	-2.643958	-4.632556
H	-2.970940	-0.941834	-5.083237
H	-11.983566	-2.561523	3.020833
H	-12.434460	-1.875871	1.450856
H	-10.212553	-3.950768	1.902644
H	-11.844302	-4.367372	1.375246
H	-11.556946	-3.494291	-0.783518
H	-10.425674	-2.264355	-0.267770
H	-9.452383	-5.131155	-0.162660

H	-9.677938	-4.392858	-1.758624
H	-6.923269	-2.527995	0.778346
H	-8.607249	-2.672444	1.276637
H	-6.149302	-3.981922	-0.886560
H	-7.123757	-4.030902	-2.364533
H	-8.590987	-1.424807	-0.596389
H	-7.102832	-1.931601	-1.428858
H	-7.783832	-5.328204	-5.573550
H	-6.088255	-5.087009	-6.125089
H	-6.402439	-5.920560	-4.583071
H	-0.679490	-1.205738	-5.812365
H	-0.563166	-2.846665	-5.155039
H	-2.597427	-2.063421	-7.309937
H	-0.977553	-2.668526	-7.670234
H	-1.475777	-4.808612	-6.447089
H	-3.117963	-4.230706	-6.058602
H	-2.651910	-4.530173	-7.748949
H	-2.368788	1.535975	-2.692033
H	-2.470089	0.874590	-4.331074
H	-4.148345	2.676696	-3.853681
H	-4.911566	1.638932	-2.655627
H	-6.142158	1.487301	-4.816255
H	-4.656922	1.011652	-5.667382
H	-5.400785	-0.090208	-4.479395
H	-1.607829	-4.063065	-3.871795
H	-0.464670	-3.793041	-2.533874
H	-2.018469	-5.142007	-1.050486
H	-3.134854	-5.286296	-2.424237
H	-1.725895	-7.363502	-2.179463
H	-1.392125	-6.546060	-3.722395
H	-0.248764	-6.390932	-2.363752
H	-8.203035	-5.173143	1.713930
H	-6.507173	-4.919326	1.226429
H	-6.243642	-3.204500	3.007693
H	-7.893386	-3.521387	3.560202
H	-6.256890	-4.824127	4.953276
H	-7.316601	-5.925337	4.045930
H	-5.650694	-5.635123	3.493531
H	-7.522633	-6.217005	-0.257726
H	-8.010236	-6.186495	-1.961795
H	-6.063938	-7.546356	-1.937938
H	-5.464383	-5.983105	-2.514619
H	-4.658945	-5.430220	-0.183448
H	-5.270325	-6.999473	0.396623
H	-3.929147	-6.937647	-0.771173
H	-9.957871	-2.195686	-2.552358
H	-8.495017	-2.893491	-3.315073
H	-8.887872	0.081198	-2.702092
H	-7.437538	-0.688550	-3.402524
H	-8.879662	0.297665	-5.206923
H	-8.718287	-1.468312	-5.363503
H	-10.224701	-0.760058	-4.715834
H	-1.422834	-2.268502	1.059059
C	-3.201328	-4.505503	2.407832
H	-6.045313	-0.138425	-1.698887
H	-6.286287	0.160469	0.069159
H	-4.727339	0.533149	-0.683945
H	-2.444043	-2.023289	2.508371
H	-2.754784	-4.118264	0.335770
H	-2.171167	-4.759144	2.707516
H	-3.703373	-4.006805	3.249641

H	-3.732236	-5.441787	2.186783

187

3c-P1-PO1 ΔE=14.08 SI

C	-0.003558	-1.493054	2.409313
C	0.434652	-2.793756	2.817615
C	1.781576	-2.992962	3.196028
C	2.716015	-1.975868	3.127436
C	2.265987	-0.693695	2.744196
C	0.954994	-0.432239	2.398826
C	-0.458749	-3.912012	2.838031
N	-1.630911	-3.944541	2.290926
Co	-2.335333	-2.549699	1.268683
O	-3.253268	-1.131941	0.105710
C	-4.358486	-0.612630	0.301121
O	-5.284683	-0.517714	-0.656337
C	-4.947507	-1.240496	-1.879068
C	4.191010	-2.215781	3.316185
C	5.008918	-1.912083	2.047020
C	4.823464	-2.893674	0.879084
C	3.400594	-2.935843	0.323441
N	3.222419	-3.653865	-1.018196
C	3.330067	-2.676965	-2.181754
C	4.665736	-1.978352	-2.359892
C	4.707914	-1.293091	-3.737031
C	3.557254	-0.312963	-3.971276
C	0.485435	0.921718	1.964358
O	-1.219564	-1.200917	2.065956
N	-3.282227	-4.002996	0.414890
C	-3.140303	-4.373446	-0.814666
C	-2.441500	-3.560218	-1.777830
C	-2.818876	-3.621246	-3.126437
C	-2.238938	-2.790462	-4.077489
C	-1.224084	-1.916097	-3.650593
C	-0.806519	-1.834609	-2.326824
C	-1.400863	-2.681044	-1.352984
C	-2.712750	-2.796980	-5.510578
C	-2.591490	-4.161562	-6.205538
C	-1.144161	-4.685095	-6.223987
C	-0.736425	-5.577602	-5.050451
N	-0.754877	-7.085203	-5.339686
C	-0.319045	-7.773621	-4.061294
C	1.104261	-7.472361	-3.619775
C	1.522018	-8.445208	-2.510267
C	1.798740	-9.867237	-2.999860
C	0.157805	-0.782568	-1.858256
O	-0.957733	-2.632770	-0.116992
C	-3.727957	-4.890558	1.483868
C	-4.438396	-6.184419	1.131294
C	-4.782050	-6.897404	2.449869
C	-3.541763	-7.120873	3.328385
C	-2.770738	-5.817854	3.601814
C	-2.433906	-5.188048	2.257475
C	-2.194654	-7.478997	-5.634269
C	-2.445291	-8.922564	-6.034696
C	-3.953788	-9.188117	-6.062840
C	-4.272012	-10.625164	-6.471887
C	0.188718	-7.377532	-6.498627
C	0.725108	-8.801463	-6.641287
C	1.964503	-8.791891	-7.552735
C	3.219832	-8.282187	-6.844781
O	-3.777564	-2.354781	2.518056

C	-3.797054	-1.210637	3.313372
C	-3.801175	0.080583	2.479583
O	-4.790645	-0.007582	1.395319
C	4.218229	-4.787429	-1.156367
C	4.114714	-5.563991	-2.451608
C	5.100645	-6.733077	-2.463272
C	5.021735	-7.495481	-3.786058
C	1.777123	-4.176808	-1.073342
C	1.520185	-5.412318	-0.232274
C	0.056125	-5.850829	-0.326319
C	-0.191321	-7.116604	0.491206
O	2.215128	-4.890533	-6.546483
C	2.806350	-4.421223	-5.325181
O	1.962040	-4.362442	-4.393250
C	3.082924	-4.833277	-7.685196
O	4.024467	-4.155148	-5.337132
O	-3.000535	-6.844841	-2.576190
C	-3.003155	-7.969811	-2.004259
O	-2.947278	-8.279594	-0.798865
O	-3.050788	-9.040559	-2.956667
C	-3.019789	-10.347629	-2.377229
H	-1.865872	-5.912315	1.655431
H	-4.362825	-4.265305	2.132115
H	-3.811380	-6.838429	0.499044
H	-5.360836	-5.961861	0.568391
H	-5.518816	-6.294164	3.011686
H	-5.256441	-7.867123	2.230401
H	-2.866573	-7.828925	2.815280
H	-3.833462	-7.587328	4.283572
H	-3.377158	-5.103449	4.184802
H	-1.856500	-6.033147	4.179836
H	-0.103516	-4.823544	3.335524
H	2.987519	0.126821	2.696258
H	2.096016	-3.997794	3.495183
H	-0.233587	1.338539	2.688031
H	-0.047320	0.845157	1.004005
H	1.325758	1.622259	1.861704
H	-3.509027	-5.350524	-1.167417
H	-0.766426	-1.246201	-4.384178
H	-3.602897	-4.329319	-3.405956
H	-0.401939	0.077109	-1.451097
H	0.794892	-0.420812	-2.676268
H	0.778523	-1.146103	-1.028633
H	4.575286	-1.580095	4.133951
H	4.363937	-3.260900	3.625486
H	4.777664	-0.890156	1.700693
H	6.081566	-1.914172	2.306697
H	5.532066	-2.600828	0.090268
H	5.118700	-3.900528	1.219519
H	2.735668	-3.443441	1.026674
H	2.992980	-1.925469	0.175547
H	1.562575	-4.353676	-2.136615
H	1.144790	-3.347776	-0.721845
H	2.529650	-1.950632	-1.996583
H	3.049576	-3.249996	-3.080892
H	4.069285	-5.433011	-0.278803
H	5.216022	-4.343163	-1.069052
H	3.302188	-3.789075	-7.959169
H	4.034851	-5.347809	-7.492667
H	2.543570	-5.325597	-8.507058
H	-3.766650	-2.466061	-5.555533

H	-2.129635	-2.055363	-6.082874
H	-3.255895	-4.883136	-5.703800
H	-2.968071	-4.057202	-7.237801
H	-0.931661	-5.187116	-7.179073
H	-0.443895	-3.832968	-6.207019
H	0.294082	-5.325042	-4.760337
H	-1.400406	-5.449441	-4.184872
H	-0.360033	-7.104547	-7.410306
H	1.012989	-6.657762	-6.392422
H	-0.475480	-8.845688	-4.215879
H	-1.047563	-7.440266	-3.309052
H	-2.743185	-7.240122	-4.705580
H	-2.523500	-6.810152	-6.440906
H	-2.081165	-10.521619	-1.827984
H	-3.856427	-10.496912	-1.679773
H	-3.101125	-11.055183	-3.214635
H	4.833606	-1.233528	-1.562096
H	5.494673	-2.703406	-2.323343
H	5.673244	-0.767435	-3.829414
H	4.679535	-2.082832	-4.507260
H	3.703959	0.242222	-4.910156
H	2.597372	-0.843463	-4.055355
H	3.474170	0.422479	-3.153573
H	2.152028	-6.251199	-0.563743
H	1.761925	-5.222544	0.829359
H	-0.584390	-5.024004	0.015882
H	-0.219811	-6.024765	-1.379148
H	-0.057324	-6.924338	1.569386
H	-1.200943	-7.515220	0.308048
H	0.528825	-7.905901	0.222253
H	3.095268	-5.941193	-2.600145
H	4.319795	-4.914859	-3.318826
H	4.895692	-7.417651	-1.619009
H	6.127686	-6.353134	-2.313965
H	5.193269	-6.814060	-4.632958
H	5.758446	-8.311569	-3.831256
H	4.022376	-7.932494	-3.915240
H	1.015366	-9.223937	-5.669771
H	-0.040130	-9.468231	-7.060327
H	2.139286	-9.816400	-7.920489
H	1.758189	-8.176430	-8.447009
H	3.073775	-7.276491	-6.426771
H	3.496042	-8.954902	-6.018493
H	4.075789	-8.230787	-7.534324
H	1.816101	-7.542123	-4.456074
H	1.172437	-6.436437	-3.264344
H	0.736401	-8.468385	-1.738166
H	2.428445	-8.054232	-2.020602
H	2.115152	-10.515432	-2.168784
H	0.905944	-10.329741	-3.448225
H	2.600454	-9.876212	-3.755212
H	-2.021152	-9.140464	-7.027921
H	-1.989578	-9.611867	-5.308678
H	-4.353723	-8.983107	-5.057188
H	-4.439910	-8.484431	-6.762173
H	-5.356610	-10.808759	-6.478469
H	-3.884601	-10.851030	-7.478470
H	-3.815628	-11.339810	-5.769215
C	-5.045333	-1.302674	4.196855
H	-2.817037	0.259591	2.033708
H	-3.937779	-0.982544	-2.214066

H	-5.004871	-2.318083	-1.690041
H	-5.705667	-0.926416	-2.603302
H	-2.895793	-1.130733	3.961903
H	-4.128848	0.950153	3.064685
H	-5.012774	-2.232420	4.780923
H	-5.105297	-0.449335	4.890527
H	-5.945825	-1.317890	3.565542

187

3c-P1C-PO1 ΔE=20.61 SI

O	-3.148283	2.133277	-2.919455
C	-2.119097	1.241713	-3.221379
O	-1.434783	1.722071	-4.352475
C	-1.560377	3.150753	-4.287347
C	-2.974088	3.334195	-3.724712
O	-2.431341	-0.020413	-3.249316
Co	-1.103429	-0.662637	-1.980786
O	-1.003573	1.339623	-2.047949
C	-1.404998	2.036049	-0.855117
N	0.308623	-0.819082	-3.267896
C	1.580007	-0.810409	-3.019155
C	2.184045	-0.572076	-1.749339
C	3.593093	-0.516983	-1.668556
C	4.253589	-0.395162	-0.458876
C	3.462898	-0.250626	0.701353
C	2.079138	-0.296709	0.682067
C	1.399620	-0.548080	-0.554021
C	5.751216	-0.544187	-0.374043
C	6.157642	-2.019200	-0.558423
C	5.592950	-2.922327	0.546378
C	5.553221	-4.412237	0.201719
N	4.182439	-4.940229	-0.199098
C	3.143061	-4.657325	0.881561
C	3.572898	-5.041105	2.290270
C	2.608511	-4.467536	3.335216
C	2.729770	-2.953715	3.507762
C	1.260972	-0.070505	1.915978
O	0.115797	-0.747929	-0.500398
C	-0.224336	-1.582961	-4.395594
C	0.707921	-1.905173	-5.552572
C	-0.054412	-2.757549	-6.577366
C	-0.637360	-4.020814	-5.933362
C	-1.518505	-3.701301	-4.719468
C	-0.711650	-2.877520	-3.718160
N	-1.437481	-2.464312	-2.495147
C	-2.319752	-3.249071	-1.968129
C	-3.163733	-2.939251	-0.861090
C	-4.023443	-3.965397	-0.401547
C	-4.847847	-3.801884	0.696064
C	-4.814021	-2.545294	1.343447
C	-4.018873	-1.496521	0.914641
C	-3.168401	-1.659711	-0.228537
C	-5.765145	-4.898766	1.183270
C	-5.318528	-5.684155	2.437489
C	-4.159888	-6.673135	2.204219
C	-2.836155	-5.928548	2.175495
N	-1.611808	-6.707899	1.746555
C	-1.830583	-7.323234	0.376749
C	-0.633875	-8.069828	-0.202367
C	-1.149616	-9.121679	-1.195147
C	-1.677294	-10.371755	-0.489191
C	-3.987771	-0.173101	1.614469

O	-2.456022	-0.634847	-0.595506
C	3.695128	-4.269283	-1.479208
C	4.582007	-4.533142	-2.687661
C	3.997886	-3.906640	-3.964997
C	2.908605	-4.773056	-4.592632
C	4.335737	-6.449027	-0.382133
C	3.126016	-7.162600	-0.957688
C	3.550495	-8.556829	-1.430893
C	2.341084	-9.389592	-1.844862
C	-0.435350	-5.738654	1.693595
C	-0.597567	-4.593688	0.702183
C	-0.322566	-3.224086	1.323288
C	-1.231878	-2.866721	2.499765
C	-1.266592	-7.826008	2.720095
C	-1.236390	-7.438252	4.192274
C	-0.336090	-8.420921	4.959097
C	-0.742995	-9.886176	4.789847
O	1.732856	-9.752096	1.832697
C	2.525455	-8.569947	1.995197
O	3.757482	-8.657082	1.829903
C	2.489584	-10.938546	1.564349
O	1.805588	-7.574022	2.282363
O	-1.370739	-6.110544	-2.530033
C	-0.135969	-5.990071	-2.708619
O	0.708476	-5.269823	-2.112891
O	0.459531	-6.761381	-3.742383
C	-0.459724	-7.533403	-4.524573
H	0.173317	-3.446139	-3.375605
H	-1.103387	-1.029871	-4.759841
H	1.576620	-2.477164	-5.191370
H	1.077528	-0.972152	-6.010505
H	-0.872291	-2.152927	-7.009146
H	0.616884	-3.027155	-7.409309
H	0.182331	-4.681572	-5.604022
H	-1.222870	-4.585703	-6.677050
H	-2.407373	-3.115746	-5.017194
H	-1.859096	-4.638368	-4.258886
H	-2.390542	-4.266833	-2.370639
H	-5.450450	-2.385768	2.219378
H	-4.010008	-4.916626	-0.940603
H	-4.355670	0.625247	0.949735
H	-2.951818	0.092964	1.878436
H	-4.601625	-0.187774	2.525272
H	2.259649	-1.071098	-3.837614
H	3.963135	-0.094745	1.661776
H	4.166818	-0.597917	-2.596379
H	0.603635	-0.921823	2.122629
H	0.598484	0.799422	1.779728
H	1.902645	0.106886	2.789176
H	-6.751656	-4.454164	1.402495
H	-5.932513	-5.617509	0.362329
H	-5.054134	-4.977262	3.244515
H	-6.187437	-6.257837	2.800457
H	-4.155419	-7.438932	2.998050
H	-4.343144	-7.193069	1.251069
H	-2.910542	-5.084464	1.487301
H	-2.607023	-5.521042	3.169189
H	-0.256562	-8.154197	2.433234
H	-1.988593	-8.631579	2.529582
H	-2.117690	-6.516916	-0.312667
H	-2.678639	-8.006941	0.501201

H	0.446770	-6.366837	1.519418
H	-0.351040	-5.362336	2.719464
H	3.032641	-10.863915	0.611346
H	3.220190	-11.132135	2.363479
H	1.761207	-11.759176	1.512005
H	6.245457	0.066213	-1.148145
H	6.112067	-0.174490	0.601083
H	5.780783	-2.346581	-1.539527
H	7.257291	-2.113668	-0.595566
H	6.190721	-2.792088	1.463980
H	4.583542	-2.576083	0.810224
H	6.221605	-4.647489	-0.636746
H	5.867180	-5.037783	1.044698
H	5.213994	-6.577305	-1.031791
H	4.564670	-6.873656	0.607763
H	2.254328	-5.225567	0.580917
H	2.897179	-3.590945	0.788395
H	2.666586	-4.622831	-1.650655
H	3.646257	-3.192418	-1.256194
H	-1.194763	-6.889193	-5.029381
H	0.149886	-8.057375	-5.272690
H	-1.003256	-8.260184	-3.906242
H	0.027200	-7.352220	-0.703680
H	-0.037873	-8.575061	0.570822
H	-1.925082	-8.666280	-1.833184
H	-0.329756	-9.407645	-1.867781
H	-2.044576	-11.118150	-1.210357
H	-0.880705	-10.836126	0.112601
H	-2.512157	-10.141650	0.193129
H	-0.814116	-6.429384	4.318975
H	-2.252054	-7.427454	4.626111
H	0.694373	-8.284855	4.590499
H	-0.349214	-8.146752	6.027342
H	-0.572413	-10.225956	3.757817
H	-0.141435	-10.533721	5.445124
H	-1.804642	-10.043336	5.043170
H	0.035574	-4.755727	-0.184977
H	-1.616328	-4.568459	0.296272
H	-0.433399	-2.465430	0.538402
H	0.730613	-3.167098	1.642269
H	-2.291169	-2.944895	2.217226
H	-1.062802	-3.510221	3.378953
H	-1.066437	-1.830081	2.828871
H	2.360597	-7.264282	-0.174278
H	2.658919	-6.617806	-1.791425
H	4.247737	-8.452916	-2.282986
H	4.093286	-9.060195	-0.613293
H	2.641019	-10.364151	-2.260414
H	1.700097	-9.574757	-0.971075
H	1.744964	-8.853174	-2.600130
H	3.575767	-6.138412	2.380692
H	4.577814	-4.651187	2.518350
H	2.809802	-4.972792	4.293796
H	1.580657	-4.745029	3.065072
H	1.986037	-2.568290	4.221766
H	3.731083	-2.681106	3.879861
H	2.574510	-2.417047	2.560819
H	5.592253	-4.130443	-2.519056
H	4.690230	-5.616522	-2.856853
H	3.602336	-2.903903	-3.726102
H	4.820137	-3.751607	-4.683801

H	2.542303	-4.334989	-5.532236
H	3.300859	-5.775442	-4.828451
H	2.058498	-4.914885	-3.911214
H	-0.787830	3.561545	-3.615969
C	-4.061270	3.376225	-4.781183
H	-1.472887	3.111196	-1.073125
H	-2.360962	1.661248	-0.473193
H	-0.611385	1.852716	-0.120352
H	-1.435816	3.555849	-5.299619
H	-3.038914	4.197759	-3.043617
H	-5.049055	3.296188	-4.306382
H	-4.016793	4.319321	-5.347810
H	-3.938556	2.533888	-5.476091
